# Dysregulated Cell Signaling Pathways in Prostate Tumoral Plasticity—Checkpoints

**DOI:** 10.32604/or.2026.072421

**Published:** 2026-05-21

**Authors:** Elena Matei, Ionuț Ciprian Iorga, Mariana Deacu, Georgeta Camelia Cozaru, Gabriela Isabela Băltățescu, Manuela Enciu

**Affiliations:** 1 Center for Research and Development of the Morphological and Genetic Studies of Malignant Pathology, “Ovidius” University of Constanta, 145 Tomis Blvd., Constanta, Romania; 2 Medicine Faculty, “Ovidius” University of Constanta, 1 Universitatii Street, Constanta, Romania; 3 Urology Department, “Sf. Apostol Andrei” Emergency County Hospital, 145 Tomis Blvd., Constanta, Romania; 4 Clinical Service of Pathology, “Sf. Apostol Andrei” Emergency County Hospital, 145 Tomis Blvd., Constanta, Romania

**Keywords:** Epithelial-mesenchymal transition (EMT), necrosis-apoptosis continuum, DNA damage, transcription factor p53, autophagy, microenvironment

## Abstract

***Objectives:*** Deregulated plasticity is involved in initiation, progression, metastasis, and resistance to therapy of various cancers. Our study aimed to present new checkpoints involved in complex biological processes that sustain epithelial-mesenchymal transition (EMT) variability and heterogeneity in prostate tumor cell plasticity. ***Methods:*** Dysregulated cell signaling pathways involved in prostate EMT heterogeneity were analyzed by intrinsic and extrinsic factors such as cell cycle phases by propidium iodide (PI) stain, apoptosis by caspase-3/7 biochemical cascade DEVDase enzyme activity by Magic Red stain (DEVD-MR)/propidium iodide stain, autophagy and nuclear shrinkage by Hoechst/acridine orange stain, evasion of immune surveillance by GPIba platelet glycoprotein conjugated with phycoerythrin (CD42b-PE) stain, oxidative stress by total reactive oxygen species (ROS) count by flow cytometry. Adaptation of the microenvironment involved in prostate EMT heterogeneity was analyzed by immunohistochemistry (IHC). ***Results:*** In our study, in benign prostatic hyperplasia (BPH) tissue samples, the low S-proliferative phase category of the cell cycle (SH: <7%) represents an independent predictor and a favorable prognostic biomarker for patient survival, as it is reported to dysregulate cell signaling pathways that characterize EMT heterogeneity. In prostate cancer tissue samples (PCa), the high S-proliferative phase category of the cell cycle (SCA, >12%) had an unfavorable prognostic role in patient survival rate, characteristically for EMT heterogeneity and aggressive phenotype involved in prostate tumoral cell plasticity, serving as a dependent predictor for the molecular mechanisms network, including late apoptosis, necrosis, autophagy, evasion of immune surveillance, cell cycle arrest in G0/G1 or G2/M phases, and oxidative stress. ***Conclusion:*** Low and high S-proliferative phase categories of the cell cycle, dysregulated early, late apoptosis via caspase-3/7 signaling pathway represent important checkpoints involved in EMT heterogeneity, and serve as independent or dependent predictor biomarkers for BPH and PCa patient prognostic survival rates, targeting personalized cancer therapy development.

## 1 Introduction

Prostate lineage plasticity is a key driver of tumor progression, and epithelial-mesenchymal transition (EMT) heterogeneity plays an important role in regulating tumoral cellular plasticity [1,2,3]. Epithelial cell–cell adhesion and apico-basal polarity loss, and the adoption of mesenchymal aspects, such as invasion and migration, represent specific EMT heterogeneity characteristics. EMT heterogeneity variability includes one or more hybrid epithelial/mesenchymal states [4,5], which represent the base of the dysregulated molecular signaling pathways involved in the complex biological processes.

In benign prostate hyperplasia (BPH) with EMT heterogeneity, aggressive phenotype, dysfunction of complex biological processes, such as apoptosis, cell cycle, and autophagy, is driven by deregulated molecular signaling pathways, leading to lineage plasticity and a modified and variable microenvironment with evasion of immune surveillance [6]. Apoptosis represents an essential biological process to study because its suppression in carcinogenesis plays a vital role in cancer progression [7,8,9]. Apoptosis is initiated when DNA damage is unrepaired, and the transcription factor p53, a tumor suppressor, activates DNA repair proteins, leading to cell cycle arrest in G0/G1 or S phases [10]. Deregulated molecular signaling pathway involved in cell cycle and apoptosis leads to tumorigenesis. Apoptosis, programmed cell death, is characterized by morphological changes and enzyme-dependent biochemical processes and plays a crucial role in clearance, causing minimal damage to surrounding tissues. Necrosis, uncontrolled cell death, occurs following severe injury, resulting from the spillage of cell content into surrounding tissues and subsequent damage. Cell death is influenced by various factors, including microenvironment, developmental stage, tissue type, and molecular signaling pathways [11]. In prostate adenocarcinomas, the apoptosis-necrosis continuum represents a specific adaptation to EMT heterogeneity, an aggressive phenotype involved in cell plasticity. The necrosis-apoptosis continuum suggests that apoptosis and necrosis have morphological expressions that are part of a biochemical network [12]. In tumor cells, apoptosis depletion is determined by activating tumor-infiltrating lymphocytes through the type-II transmembrane protein in the tumor necrosis factor (FasL)-mediated pathway [13]. Genetic errors involved in cell proliferation and dysregulated apoptosis-signaling pathways lead to cancer development [14], influencing tumoral cells’ susceptibility to the action of chemotherapeutic drugs [15,16].

Autophagy is a protective mechanism that limits uncontrolled cell proliferation and prevents apoptosis. Apoptosis and autophagy exhibit distinct morphological characteristics, biochemical features, and molecular signaling pathways [17]. In a dysregulated microenvironment, integrins bind to the extracellular matrix (ECM), determining cell proliferation. When integrins are dissociated from the ECM in response to various factors, they trigger apoptosis and maintain homeostasis [18]. β1 integrin-ECM binding inhibits cytochrome c release from mitochondria and activates the phosphatidylinositol 3-kinase—Protein Kinase B (PI3K-AKT) signaling pathway, decreasing the apoptosis [19]. Additionally, cell death signaling pathways are dependent on intracellular ROS levels. Prostate tumor cells exhibit apoptosis at low reactive oxygen species (ROS) levels and necrosis when exposed to high ROS concentrations [20,21,22].

Our objectives highlight new checkpoints involved in biological processes dysfunction that sustain EMT variability and heterogeneity in tumoral cellular plasticity. Laboratory analyses in scientific work serve as the basis for clinical findings. Adapting microenvironment, dysregulated biological processes and molecular signaling pathways, in particular escaping from cell apoptosis, evasion of immune surveillance involved in benign prostatic hyperplasia (BPH) and prostate cancer (PCa), representing a study base in EMT heterogeneity that determines tumoral cell plasticity. Prostate tissue samples, after mechanical homogenization, contain epithelial cells, mesenchymal cells, immune cells (lymphocytes, platelets, etc.), and tissue fragments, and a modified and variable microenvironment sustains EMT heterogeneity. All flow cytometry and immunohistochemistry analyses are performed on complex prostate tissue samples, with various components that bind to specific fluorochromes to reveal deregulated biological processes in cell plasticity.

## 2 Materials and Methods

### 2.1 Materials

Various prostate tissue samples from transurethral prostate resections, prostate biopsy points, as well as prostatectomy pieces from diagnosed patients with benign prostatic hyperplasia (BPH) and prostate cancer (PCa), were usually received from the Urology Department, “Sf. Apostol Andrei” Emergency County Hospital to the Clinical Pathology Service, “Sf. Apostol Andrei” Emergency County Hospital, Constanta, Romania to assure pathology diagnosis.

Beginning with 15 May 2023 to 11 February 2025, informed consents were obtained from patients involved in the research activity, with a final approval by the Ethics Committee of the Research-Development Department from “Ovidius” University of Constanta, Romania, approval number 12/July 16th, 2024, being selected and divided specifically various prostate tissue samples (*n* = 72): (1) For microenvironment characterization by Hematoxylin-Eosin (H&E kit, NC1470670, Thermo Fisher Scientific, Waltham, MA, USA) stain, Cytokeratin HMW (CK34E12 clone, ZTA Z2019MT, Zeta Corporation, Mercedes Scientific, Lakewood Ranch, Florida, USA) for basal cell layer, Alpha methylacyl CoA racemase (AMACR, 13H4 clone, GA060, Dako Omnis, Agilent, Santa Clara, USA) for malignant tumor cells, and transcription factor p53 (DO-7 clone, Mouse Monoclonal Antibody, 0.1 mL Concentrate, ZTA Z2029ML, Zeta Corporation, Mercedes Scientific, Lakewood Ranch, Florida, USA) for nuclear expression by immunohistochemistry (IHC) methods at the Clinical Service of Pathology, “Sf. Apostol Andrei”, Clinical Emergency County Hospital, Constanta, Romania; (2) Mechanically homogenizing with TissueRuptor II (120 V, 60 Hz, 9002755, Qiagen, Germantown Rd, USA) to study cell cycle, caspases 3/7 activity, nuclear shrinkage, autophagy, cell adhesion, and oxidative stress by flow cytometry analysis at the Cell Biology Department, CEDMOG, Ovidius University from Constanta, Romania.

### 2.2 Morphological Evaluation of Prostate Tissue Samples

#### 2.2.1 Hematoxylin-Eosin Stain

After prostate tissue specimens were fixed in 10% formaldehyde, paraffin-embedded, 4 μm-thick sections were evaluated under a Primo Star microscope (model 415500-0057-000, Zeiss, Gottingen, Germany with Axiocam 105 color camera) using a standard laboratory stain (H&E kit, NC1470670, Thermo Fisher Scientific, Waltham, MA, USA).

#### 2.2.2 Immunohistochemistry Analysis

Cytokeratin HMW (CK34E12 clone, ZTA Z2019MT, Zeta Corporation, Mercedes Scientific, Lakewood Ranch, Florida, USA) is a specific and valuable biomarker for differential identification of squamous carcinomas and adenocarcinomas, distinguishing between BPH and malignant tumors. AMACR (Anti-Human P504S Monoclonal Rabbit, 13H4 clone, GA060, Dako Omnis, Agilent, Santa Clara, USA) is an antibody against alpha-methylacyl-CoA racemase, a tumor marker expressed in prostate cancer, confirming the heterogeneity of prostate carcinomas. Transcription factor p53 (DO-7 clone, Mouse Monoclonal Antibody, 0.1 mL Concentrate, ZTA Z2029ML, Zeta Corporation, Mercedes Scientific, Lakewood Ranch, Florida, USA) is a tumor suppressor gene expressed in various tissue types, with important roles in cell cycle and apoptosis.

Immunohistochemical staining was performed using the HRP-DAB methodology (ZD15 Zeta MAX HRP Polymer Detection Kit with DAB Chromogen, Zeta Corporation, ZC20210101A, Arcadia, CA, USA). Formalin-fixed, paraffin-embedded tissue samples were used, sectioned at a thickness of 2–3 μm. The staining protocol was carried out according to the manufacturer’s recommendations.

The protocol included deparaffinization in Histoalcol 99 (A0146, Diapath S.p.A., Martinengo, Italy)/xylene (1330-20-7, Lach:ner, Tovární, Czech Republic)/Ottix shaper (X0096, Diapath S.p.A., Martinengo, Italy), followed by successive alcohol baths (100%, 80%, 70%, ethanol solution, >99.7% volume denatured with 1% methyl ethyl ketone (MEK), 1% isopropanol and 10 ppm denatonium benzoate, 180722B100, Tunic, Romania) and distilled water, each for 7–10 min. Antigen retrieval was performed by heating at 110°C for 40 min, followed by gradual cooling in distilled water. Sections were then drained and washed with wash buffer.

Endogenous peroxidase activity was blocked using Hydrogen Peroxide for 5 min (room temperature), followed by washing with wash buffer. 200 μL of the HMWCK primary antibody (CK34E12 clone, ZTA Z2019MT, dilution range 1:100, Zeta Corporation, Mercedes Scientific, Lakewood Ranch, Florida, USA) or AMACR (200 μL, Anti-Human P504S Monoclonal Rabbit, 13H4 clone, GA060, Ready-to-Use, Dako Omnis, Agilent, Santa Clara, USA), or p53 (200 μL, DO-7 clone, Mouse Monoclonal Antibody, 0.1 mL Concentrate, dilution range 1:100, ZTA Z2029ML, Zeta Corporation, Mercedes Scientific, Lakewood Ranch, Florida, USA) was applied for 30 min, at room temperature, after which the sections were washed again.

Detection was performed using the ZD15 Zeta MAX HRP Polymer Detection Kit with DAB Chromogen (Zeta Corporation, ZC20210101A, Arcadia, CA, USA). An amplification step was applied for 10 min, followed by washing, then incubation at room temperature with 100 μL Zeta HRP Anti-Mouse/Anti-Rabbit (Ready-to-use, Zeta Corporation, ZC20210101A, Arcadia, CA, USA) for 10 min. After washing, DAB chromogen was applied for 5 min, at room temperature. The sections were rinsed with distilled water, counterstained with hematoxylin for 5 min, washed with running water, dehydrated through alcohols, cleared, and mounted in Bio Mount HM, 05-BMHM100, lot 25142, Bio-Optica, Milano, Italy).

Prostate adenocarcinomas, by Gleason classification, were divided: (1) Well-differentiated—Gleason 6 score; (2) Moderately differentiated—Gleason score 7; (3) Poorly differentiated—Gleason scores 8–10. In function of patient survival prognostic grade, PCa cases were divided: (1) Gleason score ≤ 6; (2) Gleason score 7 (Gleason pattern 3 + 4); (3) Gleason score 7 (Gleason pattern 4 + 3); (4) Gleason score 8 (Gleason pattern 4 + 4); (5) Gleason score 9–10 [23,24,25,26,27].

### 2.3 Dysfunction of Complex Biological Processes by Flow Cytometry Analysis

#### 2.3.1 Samples and Controls

In the Cell Biology Department, CEDMOG, Ovidius University from Constanta, Romania, mechanically homogenizing prostate tissue samples (*n* = 72) by flow cytometry analyses were divided: (1) Prostate benign hyperplasia (H), with low S-phase category, G2/M phase arrest (*n* = 24); (2) Adenocarcinomas (AC) with high S-phase category (>12%), G1/G2 or G2/M phases arrest, apoptosis-necrosis continuum phenotype (*n* = 30); (3) Carcinoma (CA) tissue samples, uncontrolled S-phase proliferation (*n* = 6); (4) The negative control is represented by prostate healthy tissue samples (*n* = 12). Cut-offs for S-proliferation phase categories of the cell cycle (<7% and >12%) used in prognostic classification were established by flow cytometry analyses [28].

#### 2.3.2 Equipment

A calibrated Attune Acoustic focusing cytometer (model: 4445280R, Applied Biosystems, Waltham, MA, USA) by Attune performance tracking beads, labeling, and detection (four intensity levels of beads population, Life Technologies, Europe BV, Bleiswijk, The Netherlands) [29] was used to analyze prostate tissue samples. Forward Scatter (FSC) and Side Scatter (SSC) flow cytometer analyses were performed on 10,000–20,000 cells per sample, and the obtained data were interpreted using Attune Cytometric Software v.1.2.5, 2010.

#### 2.3.3 Methods

##### Cell Cycle

Homogenized prostate tissue samples (200 μL) were fixed in absolute ethanol, in darkness, for 30 min. 10 μL of propidium iodide (PI, 00-6990, Invitrogen, Thermo Fisher Scientific, Waltham, MA, USA) were added to tubes with prostate samples (200 μL of cells at 10^6^ cells/mL) and kept in the darkness at room temperature, for 30 min. 1 mL of flow cytometry staining buffer (FCB, 00-4222-26, eBioscience™, Invitrogen, Life Technologies Carlsbad, CA, USA) was added to the tubes. Cell cycle phases were analyzed using the BL2 channel (PI, Emission: 533/617 nm) [30].

##### Caspase-3/7 Activity

The cell death mechanism was highlighted using the DEVD-MR/PI methodology (DEVD-MR, FAM Caspase-3/7 Assay Kit, ab270771, Abcam, Waltham, MA, USA) in prostate tissue samples. 20 μL of DEVD-MR solution and 20 μL of PI (00-6990, Invitrogen, Thermo Fisher Scientific, Waltham, MA, USA) were added to tubes containing homogenized prostate tissue samples (200 μL of cells at 10^6^ cells/mL), mixed and incubated, for 30 min, at room temperature, in the darkness. After the addition of 1 mL FCB (00-4222-26, Invitrogen, Life Technologies Carlsbad, CA, USA) in tubes, viability, early, late apoptosis, and necrosis were assessed using the BL3 channel (Magic Red, Emission: 592 nm/628 nm) and the BL2 channel (PI, Emission: 533/617 nm) [31].

##### Nuclear Shrinkage and Lysosomal Activity

In tubes, homogenized prostate tissue samples (200 μL of cells at 10^6^ cells/mL) were stained with 2 μL of Hoechst 33342 (200 μg/mL, ab270771, Abcam, Waltham, MA, USA) and 50 μL of acridine orange (1.0 μM, AO, ab270771, Abcam, Waltham, MA, USA). Samples were mixed and incubated at room temperature in the dark for 30 min. After the addition of 0.5 mL FCB (00-4222-26, Invitrogen, Life Technologies Carlsbad, CA, USA), prostate cells were analyzed by flow cytometry using the VL2 channel (Hoechst, UV-filter with excitation at 365 nm and emission at 480 nm) and BL1 channel (AO, blue light 480 nm excitation filter) [6].

##### Cell Adhesion

Anti-CD42b-PE (HIP1, 12-0429-42, 0.5 μg/test, Invitrogen, eBioscience^TM^, Life Technologies, Carlsbad, CA, USA) monoclonal antibodies conjugated with phycoerythrin (PE) were used to assess GPIba platelet glycoprotein. In tubes with homogenized prostate tissue samples (200 μL of cells at 10^6^ cells/mL), 5 μL of CD42b-PE was added, mixed, and incubated in the dark, for 25 min, at 37°C. One milliliter of FCB (00-4222-26, Invitrogen, Life Technologies Carlsbad, CA, USA) was added to the tubes before analysis and the samples were analyzed on the BL2 channel (PE, Excitation/Emission:565/576 nm) [6].

##### Total Reactive Oxygen Species (ROS)

Oxidative stress in homogenized prostate tissue samples was quantified using the Total Reactive Oxygen Species (ROS) Assay Kit (520 nm, 88-5930-74, 500× ROS stock solution, Invitrogen, Life Technologies Carlsbad, CA, USA) methodology. After 60 min of incubation at 37°C and 5% CO_2_, homogenized prostate samples (500 μL of cells at 10^6^ cells/mL) with 50 μL of 1× ROS work solution were analyzed using the BL-1 channel [30].

### 2.4 Data Analysis

Cell cycle (%), caspase-3/7 activity (%), nuclear shrinkage (%), autophagy (%), cell adhesion (%), and oxidative stress count (×10^6^) were reported as mean± standard deviation (SD). The Mann-Whitey test by MedCalc v20.111 Software Ltd., 2010 (Ostend, Belgium) was used to compare the means between parametric results from the control and three experimental groups (PCs tissue samples), with normal distributions. Differences between control and PCs tissue samples with *p* < 0.05, were considered statistically significant.

The predictor factors, low and high S-proliferative phases of the cell cycle, early and late apoptosis, reported to dysregulate biological processes in BPH and PCa tissue samples were assessed by Least squares multiple regression (R^2^) by MedCalc v20.111 Software Ltd., 2010, Ostend, Belgium. Differences between variables with *p* < 0.05 were considered statistically significant.

**Figure 1:**
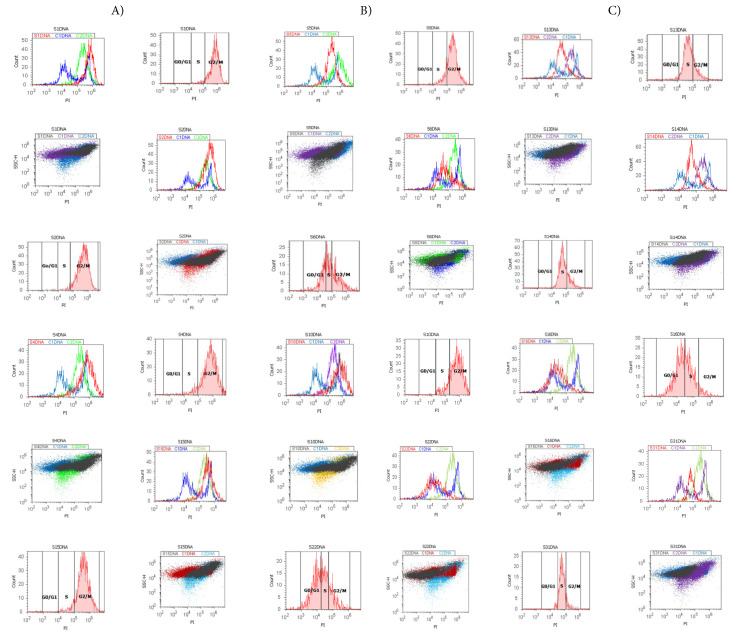
Cell cycle signaling pathway by propidium iodide (PI) stain. (**A**) Benign prostate hyperplasia (BPH) tissue samples, low S-phase category (<7%), G2/M phase arrest: G0/G1: S1 = 0.00%; S2 = 0.23%; S4 = 0.45%; S15 = 0.06%; S: S1 = 2.92%; S2 = 1.85%; S4 = 5.73%; S15 = 7.22%; G2/M: S1 = 95.83%; S2 = 96.98%; S4 = 90.82%; S15 = 91.93%. (**B**) Adenocarcinoma (AC) tissue samples, high S-phase category (>12%), G0/G1 or G2/M phases arrests, apoptosis-necrosis continuum phenotype: G0/G1: S5 = 0.37%; S6 = 30.10%; S10 = 0.21%; S22 = 47.80%; S: S5 = 13.79%; S6 = 33.02%; S10 = 15.12%; S22 = 31.04%; G2/M: S5 = 85.71%; S6 = 36.07%; S10 = 84.12%; S22 = 19.60%. (**C**) Carcinoma (CA) tissue samples, uncontrolled S-phase proliferation: G0/G1: S13 = 1.92%; S14: 0.21%; S16 = 52.51%; S31 = 4.34%; S: S13 = 73.71%; S14: 77.28%; S16 = 41.54%; S31 = 85.09%; G2/M: S13 = 22.94%; S14: 22.18%; S16 = 5.72%; S31 = 9.81%. Legend: C1DNA-negative control represented by healthy tissue sample; C2DNA-positive control represented by adenocarcinoma or carcinoma tissue samples. Negative and positive controls were extrapolated to graphs to interpret the experimental samples.

**Figure 2:**
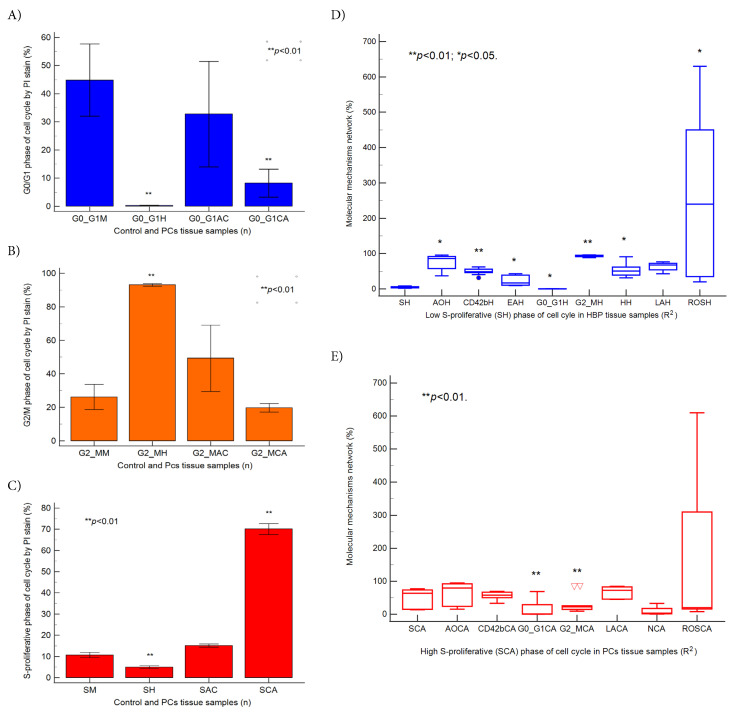
Cell cycle phase statistics in BPH and PCa (**A**–**C**), **p < 0.01 represents significant statistical differences between control and PCs tissue samples by Mann-Whitney test by MedCalc software, Ostend, Belgium; (**D**,**E**) Predictor factors represented by coefficient of multiple regression (R^2^) in BPH and PCa tissue samples, **p < 0.01 and *p < 0.05 represent significant statistical differences between variables made by Least squares multiple regression (R2) by MedCalc v20.111 Software Ltd., Ostend, Belgium. BPH: Low S-proliferative phase of cell cycle (SH): AOH: R^2^ = 0.045, *p = 0.014; CD42bH: R^2^ = −0.174, **p = 0.006; EAH: R^2^ = 0.128, *p = 0.025; G0/G1H: R^2^ = −3.828, *p = 0.011; G2/MH: R^2^ = −0.082, **p = 0.005; HH: R^2^ = −0.018, *p = 0.019; LAH: R^2^ = 0.028, p = 0.130; ROSH: R^2^ = −0.302, *p = 0.014. PCs: High S-proliferative phase of cell cycle (SCA): AOCA: R^2^ = 0.243, *p* = 0.120; CD42bCA: R^2^ = 0.136, *p* = 0.553; G0/G1CA: R^2^ = −1.357, **p = 0.008; G2/MCA: R^2^ = −1.126, **p = 0.008; LACA: R^2^ = 0.618, *p* = 0.170; NCA: R^2^ = 0.707, *p* = 0.099; ROSCA: R^2^ = 1.541, *p* = 0.255. Legend: M—healthy tissue samples; H—Benign prostate hyperplasia (BPH) tissue samples; AC—Adenocarcinoma tissue samples; CA—Carcinoma tissue samples; (∇) represents variability of samples reported to mean.

**Figure 3:**
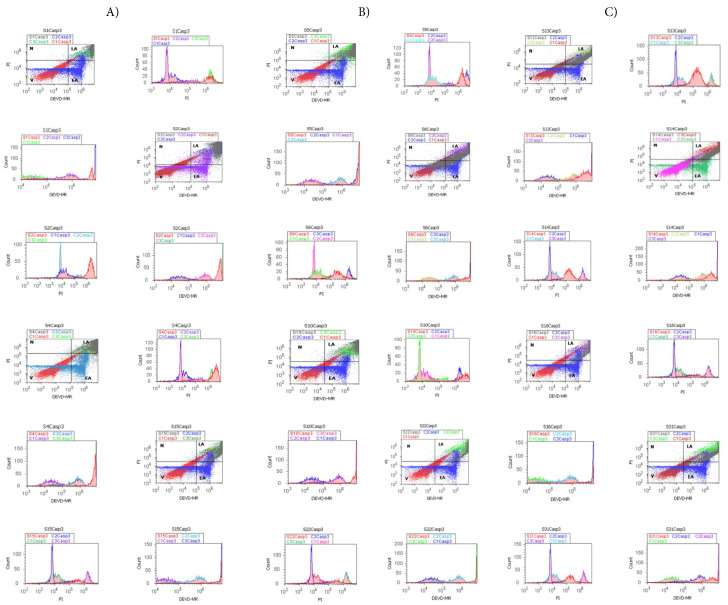
Caspases-3/7 activity (Casp3) pathway highlighted by DEVD-MR/PI stain, apoptotic signal by DEVD-MR stain (EA), and cell permeability by PI stain (LA) (**A**–**C**). (**A**) Benign prostate hyperplasia (BPH) tissue samples, low S-phase category (<7%), G2/M phase arrest: V: S1 = 5.66%; S2 = 2.64%; S4 = 4.93%; S15 = 8.81%; EA: S1 = 7.82%; S2 = 5.46%; S4 = 8.34%; S15 = 7.33%; LA: S1 = 86.32%; S2 = 91.47%; S4 = 86.05%; S15 = 83.62%; N: S1 = 0.17%; S2 = 0.41%; S4 = 0.66%; S15 = 0.23%. (**B**) Adenocarcinoma (AC) tissue samples, high S-phase category (>12%), G0/G1 or G2/M phases arrests, apoptosis-necrosis continuum phenotype: V: S5 = 1.17%; S6 = 26.97%; S10 = 6.99%; S22 = 6.43%; EA: S5 = 1.64%; S6 = 14.77%; S10 = 4.60%; S22 = 11.16%; LA: S5 = 96.35%; S6 = 57.23%; S10 = 87.28%; S22 = 82.30%; N: S5 = 0.82%; S6 = 1.01%; S10 = 1.11%; S22 = 0.10%. (**C**) Carcinoma (CA) tissue samples, uncontrolled S-phase proliferation: V: S13 = 7.54%; S14: 5.71%; S16 = 8.13%; S31 = 2.43%; EA: S13 = 4.31%; S14: 6.83%; S16 = 11.63%; S31 = 6.20%; LA: S13 = 86.91%; S14: 87.17%; S16 = 80.00%; S31 = 91.22%; N: S13 = 1.22%; S14: 5.71%; S16 = 0.22%; S31 = 0.13%. Legend: V-viability; EA-early apoptosis; LA-late apoptosis; N-necrosis; C1Casp3, C2Casp3-negative controls represented by healthy tissue samples; C3Casp3-positive control represented by adenocarcinomas or carcinomas tissue samples. Negative and positive controls were extrapolated to graphs to interpret the experimental samples.

**Figure 4:**
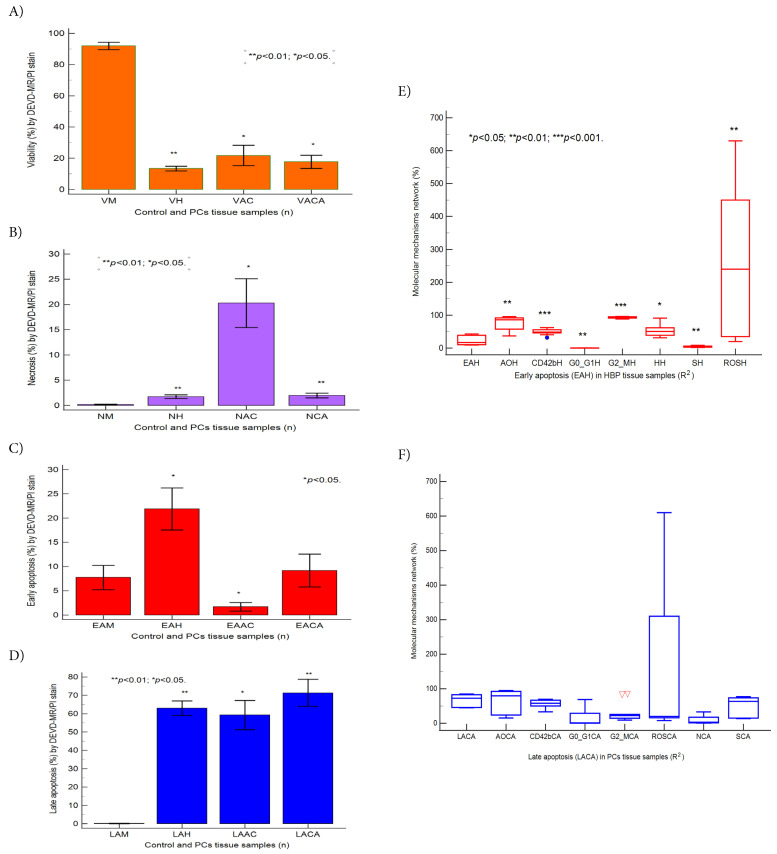
Caspases-3/7 pattern statistics in BPH and PCa (**A**–**D**), **p < 0.01 and *p < 0.05 represent significant statistical differences between control and PCs tissue samples by Mann-Whitney test by MedCalc software, Ostend, Belgium. Predictor factors represented by coefficient of multiple regression (R^2^) in BPH and PCa tissue samples (**E**,**F**), ***p < 0.001, **p < 0.01, and *p < 0.05 represent significant statistical differences between variables made by Least squares multiple regression (R^2^) by MedCalc v20.111 Software Ltd., Ostend, Belgium. BPH: Early apoptosis (EAH): AOH: R^2^ = −0.389, **p = 0.001; CD42bH: R^2^ = 1.645, ***p = 0.0005; G0/G1H: R^2^ = 33.889, **p = 0.0012; G2/MH: R^2^ = 8.851, ***p = 0.0007; HH: R^2^ = 0.118, *p = 0.010; SH: R^2^ = 9.549, **p = 0.0012; ROSH: R^2^ = 3.082, **p = 0.0021. PCs: Late apoptosis (LACA): AOCA: R^2^ = −0.347, *p* = 0.066; CD42bCA: R^2^ = −0.235, *p* = 0.426; G0/G1CA: R^2^ = 1.189, *p* = 0.218; G2/MCA: R^2^ = 1.621, *p* = 0.116; ROSCA: R^2^ = −2.530, *p* = 0.096; NCA: R^2^ = −0.988, *p* = 0.061; SCA: R^2^ = 1.112, *p* = 0.170. Legend: V-viability; EA-early apoptosis; LA-late apoptosis; N-necrosis; M-healthy tissue samples; H-Benign prostate hyperplasia (BPH) tissue samples; AC-Adenocarcinoma tissue samples; CA-Carcinoma tissue samples; (∇) represents variability of samples reported to mean.

**Figure 5:**
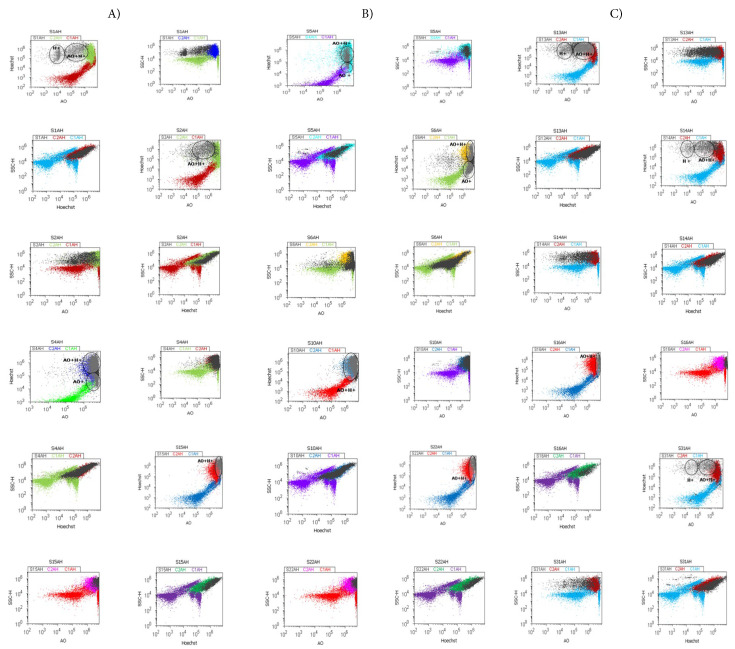

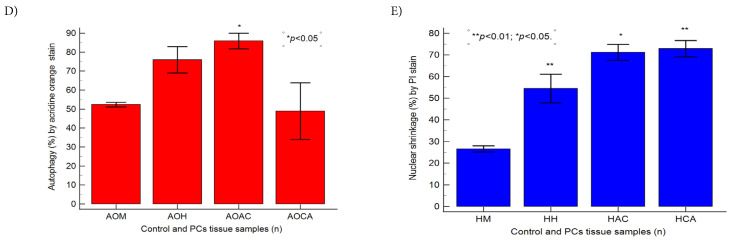
Autophagy and nuclear apoptosis (AO^+^H^+^) expressions by acridine orange/Hoechst stain to highlight nuclear shrinkage (H^+^) and lysosomal activity (AO^+^). (**A**) Benign prostate hyperplasia (BPH) tissue samples, low S-phase category (<7%), G2/M phase arrest: AO^+^H^+^: S1 = 37.74%; S2 = 48.34%; S4 = 59.96%; S15 = 85.68%; H^+^: S1 = 33.40%; S2 = 61.79%; S4 = 31.34%; S15 = 87.19%; AO^+^: S1 = 42.08%; S2 = 37.01%; S4 = 50.46%; S15 = 94.28%. (**B**) Adenocarcinoma (AC) tissue samples, high S-phase category (>12%), G0/G1 or G2/M phases arrests, apoptosis-necrosis continuum phenotype: AO^+^H^+^: S5 = 76.00%; S6 = 33.46%; S10 = 86.47%; S22 = 89.73%; H^+^: S5 = 1.47%; S6 = 63.37%; S10 = 33.05%; S22 = 91.11%; AO^+^: S5 = 17.50%; S6 = 30.76%; S10 = 86.12%%; S22 = 91.96%. (**C**) Carcinoma (CA) tissue samples, uncontrolled S-phase proliferation: AO^+^H^+^: S13 = 37.91%; S14:32.53%; S16 = 89.22%; S31 = 20.85%; *H^+^:* S13 = 85.17%; S14: 69.80%; S16 = 59.84%; S31 = 6.56%; *AO^+^:* S13 = 40.66%; S14:24.03%; S16 = 82.25%; S31 = 32.08%. (**D**,**E**) Autophagy and nuclear apoptosis pattern statistics, **p < 0.01 and *p < 0.05 represent significant statistical differences between control and PCs tissue samples by Mann-Whitney test by MedCalc software, Ostend, Belgium. Legend: C1AH-negative control represented by healthy tissue sample; C2AH-positive control represented by adenocarcinoma or carcinoma tissue samples; Negative and positive controls were extrapolated to graphs to interpret the experimental samples; PCs—prostate cancer tissue samples; M—healthy tissue samples; H—Benign prostate hyperplasia (BPH) tissue samples; AC—Adenocarcinoma tissue samples; CA—Carcinoma tissue samples.

**Figure 6:**
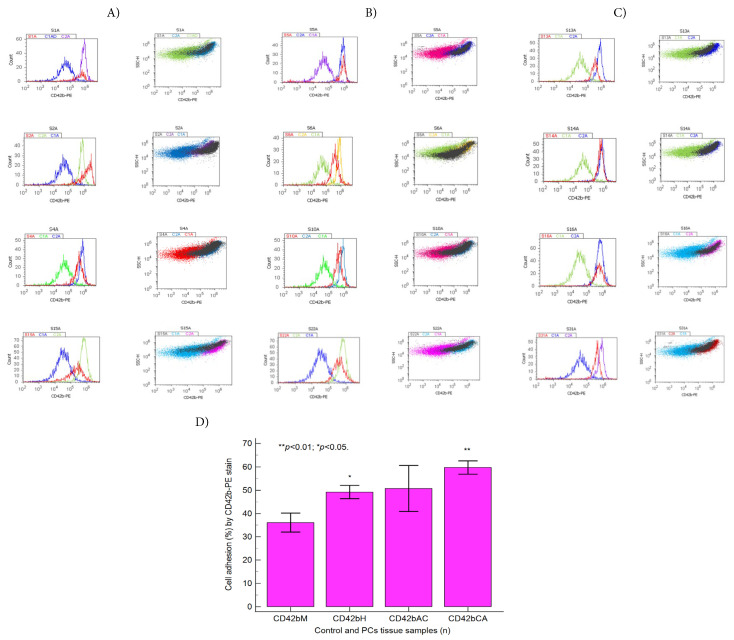
Cell adhesion pathway highlighted by integrin surface glycoprotein conjugated with phycoerythrin (CD42b-PE) stain. (**A**) Benign prostate hyperplasia (BPH) tissue samples, low S-phase category (<7%), G2/M phase arrest: CD42b: S1 = 40.60%; S2 = 55.82%; S4 = 60.02%; S15 = 49.64%. (**B**) Adenocarcinoma (AC) tissue samples, high S-phase category (>12%), G0/G1 or G2/M phases arrests, apoptosis-necrosis continuum phenotype: CD42b: S5 = 65.88%; S6 = 31.63%; S10 = 47.91%; S22 = 46.29%. (**C**) Carcinoma (CA) tissue samples, uncontrolled S-phase proliferation: CD42b: S13 = 56.83%; S14:66.95%; S16 = 56.14%; S31 = 75.65%. (**D**) Cell adhesion by integrin surface glycoprotein pattern statistics, **p < 0.01 and *p < 0.05 represent significant statistical differences between control and PCs tissue samples by Mann-Whitney test by MedCalc software, Ostend, Belgium. Legend: C1CD42b-negative control represented by healthy tissue sample; C2CD42b-positive control represented by adenocarcinoma or carcinoma tissue samples; Negative and positive controls were extrapolated to graphs to interpret the experimental samples; PCs—prostate cancer tissue samples; M—healthy tissue samples; H—Benign prostate hyperplasia (BPH) tissue samples; AC—Adenocarcinoma tissue samples; CA—Carcinoma tissue samples.

**Figure 7:**
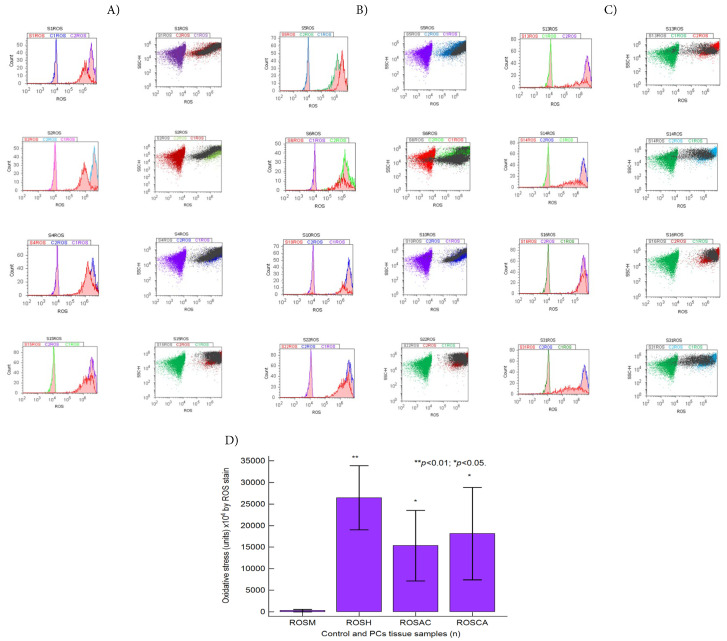
Oxidative stress pathway highlighted by reactive oxygen species (ROS) stain. (**A**) Benign prostate hyperplasia (BPH) tissue samples, low S-phase category (<7%), G2/M phase arrest: ROS: S1 = 30 × 10^6^; S2 = 35 × 10^6^; S4 = 50 × 10^6^; S15 = 40 × 10^7^. (**B**) Adenocarcinoma (AC) tissue samples, high S-phase category (>12%), G0/G1 or G2/M phases arrests, apoptosis-necrosis continuum phenotype: ROS: S5 = 52 × 10^7^; S6 = 20 × 10^6^; S10 = 20 × 10^6^; S22 = 45 × 10^7^. (**C**) Carcinoma (CA) tissue samples, uncontrolled S-phase proliferation: ROS: S13 = 20 × 10^6^; S14: 20 × 10^6^; S16 = 41 × 10^7^; S31 = 20 × 10^6^. (**D**) Oxidative stress by ROS statistics, **p < 0.01 and *p < 0.05 represent significant statistical differences between control and PCs tissue samples by Mann-Whitney test by MedCalc software, Ostend, Belgium. Legend: C1ROS-negative control represented by healthy tissue samples, C2ROS-positive control represented by adenocarcinoma or carcinoma tissue samples; Negative and positive controls were extrapolated to graphs to interpret the experimental samples. PCs—prostate cancer tissue samples; M—healthy tissue samples; H—Benign prostate hyperplasia (BPH) tissue samples; AC—Adenocarcinoma tissue samples; CA—Carcinoma tissue samples.

**Figure 8:**
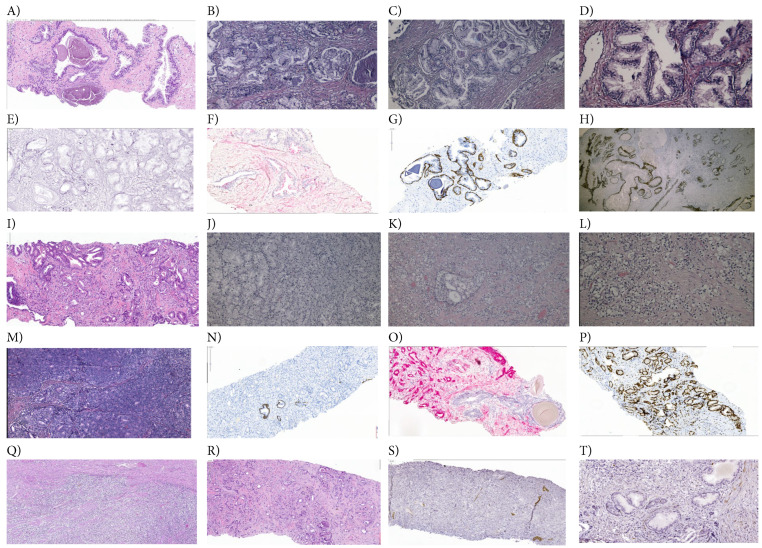
Deregulated cellular-phenotypic plasticity in BPH and PCa tissue sections. (**A**) BPH—large acini, some cystically dilated, corpora amylacea presence in lumen, H&E (×40), TURP-BP; (**B**) Prostatic glands with benign histological structure, arranged in fibromuscular stroma, H&E (×100), TURP; (**C**) BPH-characterized by proliferated glands with lobulated architecture, H&E (×100), TURP; (**D**) BPH-Large and medium-sized prostate glands, delimited by epithelial cells with papillary formation, basal cell layer is present, H&E (×400), TURP; (**E**) BPH—AMACR negative expression to highlights lesion benignity (×100); (**F**) BPH—AMACR negative expression in benign-appearing acini, focally positive in atrophic acini (×40); (**G**) BPH—HMWCK positive expression in the basal cell layer (×40); (**H**) benign prostatic tissue (HBP), which highlights HMWCK immunoexpression in basal cells, ×100; (**I**) Prostate adenocarcinoma, Gleason score sum 7 (4 + 3), represented by small proliferated glands, with an infiltrative appearance among the benign-looking glands, H&E (×40), TURP-BP; (**J**) Prostate adenocarcinoma, Gleason score sum 7 (3 + 4), moderately differentiated, primary Gleason grade 3 in 60% of neoplastic proliferation, excised by transurethral prostatic resection, H&E (×100), TURP; (**K**) Prostate adenocarcinoma, Gleason score sum 8 (4 + 4), represented by fused glands and cribriform nodules, taken by transurethral prostatic resection, H&E (×100), TURP; (**L**) Prostate adenocarcinoma, Gleason score sum 8 (4 + 4), with the presence of cells with clear cytoplasm arranged in nests, and glandular structures with poorly formed lumens, transurethral prostatic resection, H&E (×200), TURP; (**M**) Poorly differentiated adenocarcinoma, Gleason score sum 9 (5 + 4), primary Gleason grade 5 in 80% of neoplastic proliferation, with the absence of glandular differentiation and diffuse tumor architecture, with rare cribriform nodules, transurethral resection of the prostate, H&E (x200), TURP; (**N**) Prostate adenocarcinoma, Gleason score sum 7 (4 + 3), HMWCK negative expression in malignant neoplastic proliferation, positive in the basal layer within benign-appearing acini (positive control, ×40); (**O**) Moderately differentiated prostate adenocarcinoma, Gleason score sum 7 (3 + 4), AMACR positive expression in epithelial cells (×100); (**P**) Prostate adenocarcinoma, Gleason score sum 7 (3 + 4), p53 overexpressed at the nuclear level, mutational pattern, (×100); (**Q**) Poorly differentiated prostate carcinoma, Gleason score 8 (4 + 4), represented by clear cells and coalescent acini clusters, with a stromal infiltrative appearance, H&E (×100), TURP; (**R**) Poorly differentiated prostate carcinoma, Gleason score 9 (4 + 5), represented by small acini with poorly visible lumens with an infiltrative appearance and isolated or trabecular arranged neoplastic cells, H&E (×100), TURP; (**S**) Poorly differentiated prostate carcinoma, Gleason score 9 (4 + 5), CKHMW negative expression, which attests lesion malignancy, positive control, (×40); (**T**) Poorly differentiated prostate carcinoma, Gleason score 9 (4 + 5), p53 null type, mutational pattern—top of the image; negative in benign-appearing acini (×100). Legend: BPH—Benign prostatic hyperplasia; PCa—prostate cancer; TURP—transurethral prostatic resection; TURP-BP—transurethral prostatic resection and biopsy points.

Fig. 1, Fig. 3, Fig. 5, Fig. 6 and Fig. 7A–C were made by Attune Cytometric Software v.1.2.5, Applied Biosystems, 2010 (Bedford, MA, USA). Fig. 2, Fig. 4, Fig. 5D,E, Fig. 6D and Fig. 7D were generated using MedCalc v20.111 Software Ltd., 2010 (Ostend, Belgium). Fig. 8 was obtained using an Axiocam 105 color camera attached to an optical microscope (Zeiss, Gottingen, Germany).

## 3 Results

### 3.1 Cell Intrinsic Factors Involved in EMT Heterogeneity in Benign Prostate Hyperplasia and Prostate Cancer Plasticity

#### 3.1.1 Cell Cycle

Fig. 1A–C presents the cell cycle phases in BPH and PCa tissue samples by propidium iodide stain to highlight deregulated molecular signaling pathways involved in cell cycle phases, beginning with EMT heterogeneity in BPH to aggressive heterogeneity in adenocarcinomas and carcinomas. BPH tissue samples showed characteristics of cell growth inhibition through cell cycle arrest in the G2/M phase (G2/MH: 93.06 ± 2.51) compared to the negative control (G2/MM: 26.25 ± 16.80, *p* < 0.01, Fig. 1A and Fig. 2B).

The S-proliferative phase is characterized by significantly lower values in BPH tissue samples compared to healthy tissue samples (SH: 4.95 ± 2.08 vs. SM: 10.67 ± 2.70, *p* < 0.01, Fig. 1A and Fig. 2C), indicating a low S-proliferative phase category (<7%).

Cell cycle changes involved in heterogeneity phenotype of adenocarcinomas, were represented by G2/M phase blockage (G2/MAC: 49.32 ± 39.69 vs. G2/MM: 26.25 ± 16.80, *p* ≥ 0.05, Fig. 1B and Fig. 2B) or G0/G1 phase arrest reported to healthy tissue (G0/G1AC: 32.76 ± 37.48 vs. G0/G1M: 26.250 ± 16.80, *p* ≥ 0.05, Fig. 1B and Fig. 2A). The S-proliferative phase showed significantly increased expression in experimental samples compared to the negative control (SAC: 15.13 ± 1.52 vs. SM: 10.67 ± 2.70, *p* ≥ 0.05, Fig. 1B and Fig. 2C) and was included in the high S-phase category (>12%).

Heterogeneity of the EMT phenotype induced by deregulated cell signaling pathways increases tumoral aggressiveness through an apoptosis-necrosis continuum (Fig. 1B). Advanced prostate cancer tissue samples (carcinomas), with higher tumoral aggressivity, were characterized by uncontrolled S-phase proliferation, as reported in healthy tissue samples (SCA: 70.11 ± 6.30 vs. SM: 10.67 ± 2.70, *p* < 0.01, Fig. 1C and Fig. 2C).

In BPH tissue samples, the low S-proliferative phase of the cell cycle (SH: <7%) reported to molecular signaling pathways that characterize the EMT heterogeneity phenotype, serves as an independent predictor factor (*p* < 0.01; *p* < 0.05), acting as a favorable prognostic biomarker in patient survival rates (Fig. 2D). Dysregulated autophagy (AOH), cell adhesion (CD42bH), early apoptosis (EAH), late apoptosis (LAH), cell cycle (G0/G1H; G2/MH), nuclear shrinkage (NH), and oxidative stress (ROSH) maintain EMT heterogeneity phenotype, highlighting the importance of low S-proliferative phase (SH) category as favorable prognostic biomarker in patient survival rate (Fig. 2D).

In PCa samples, the high S-proliferative phase of the cell cycle (SCA, >12%) was associated with an unfavorable prognostic role in patient survival, reflecting tumoral heterogeneity and an aggressive phenotype. High S-phase category has more than 50% higher risk of death or recurrence rates, being a dependent predictor factor for molecular mechanisms network represented by dysregulated autophagy (AOCA), cell adhesion (CD42bCA), cell cycle blockage in G0/G1 or G2/M phases (G0/G1CA; G2/MCA), late apoptosis (LACA), necrosis (NCA), and oxidative stress (ROSCA) that characterize a tumoral heterogeneity aggressive phenotype (Fig. 2E, Figs. S1 and S2).

#### 3.1.2 Apoptosis and Necrosis-Caspase-3/7 Activity

Effector caspase-3/7 activity, in BPH and PCa tissue samples, reported to non-malignant adjacent prostate tissue samples (C1, C2Casp3 negative control), was analyzed by flow cytometry methods, as represented in Fig. 3A–C. The deregulated signaling pathway involved in the caspase-3/7 activation mechanism induces changes in cell viability, apoptosis, and necrosis in BPH and PCa tissue samples, with significant differences between experimental samples and controls (Fig. 3A–C and Fig. 4A–D).

Cell viability values showed significant differences in BPH, PCa tissue samples compared with controls (VH: 13.38 ± 4.92; VAC: 21.79 ± 13.00; VCA: 17.67 ± 10.43 vs. VM: 91.92 ± 5.38, *p* < 0.01; *p* < 0.05; *p* < 0.05, Fig. 3A–C and Fig. 4A).

By DEVD-MR stain, the biochemical cascade involved in pro-apoptotic signaling (EA) were showed significant changes in experimental samples compared to controls (EAH: 21.88 ± 13.71; EAAC: 1.70 ± 1.72; EACA: 9.18 ± 8.27 vs. EAM: 7.76 ± 5.61, *p* < 0.05; *p* < 0.05; *p* ≥ 0.05, Fig. 3A–C and Fig. 4C).

An inflammatory response to injury induction and high oxidative stress leads to late apoptosis (LA). Increased significant values of late apoptosis were observed in BPH and PCa tissue samples compared to healthy tissue samples (LAH: 62.99 ± 12.36; LAC: 59.23 ± 15.88; LACA:71.34 ± 18.02 vs. LAM: 0.10 ± 0.24, *p* < 0.01; *p* < 0.05; *p* < 0.01, Fig. 3A–C and Fig. 4D). As a particular characteristic observed in deregulated cellular-phenotypic plasticity in adenocarcinoma tissue samples was apoptosis-necrosis continuum (LAC: 59.23 ± 15.88 vs. LAM: 0.10 ± 0.24, *p* < 0.05; NAC: 20.28 ± 9.66 vs. NM: 0.186 ± 0.20, *p* < 0.05, Fig. 3B).

Dysregulated innate and adaptive immune system that sustains EMT heterogeneity in BPH and PCa induces significant changes in necrosis process, with higher values in adenocarcinomas (NAC: 20.28 ± 9.66, *p* < 0.05), BPH (NH: 1.73 ± 1.19, *p* < 0.01), and PCa (NCA: 1.99 ± 1.165, *p* < 0.01) than controls (NM: 0.18 ± 0.20, Fig. 3A–C and Fig. 4B).

Furthermore, multiple regression was applied to determine predictor biomarkers, including early (EAH) and late apoptosis (LACA) checkpoints, with prognostic roles in patient survival in BPH and PCa tissue samples (Fig. 4E,F). After multiple regression applied analysis, was observed that early apoptosis (EAH) in BPH tissue samples remains an independent significant predictor biomarker with a favorable prognostic value in patient survival rate (*p* < 0.01), reported to dysregulated biological mechanisms involved in cellular-phenotypic plasticity represented by autophagy (AOH), cell adhesion (CD42bH), cell cycle (G0/G1H; SH; G2/MH), nuclear shrinkage (NH), and oxidative stress (ROSH, Fig. 4E).

In PCa tissue samples, the late apoptosis (LACA) checkpoint remains a dependent predictor biomarker with unfavorable prognostic in patient survival rate, being characteristically for aggressive cellular-phenotypic tumoral plasticity that implied dysregulated biological mechanisms such as autophagy (AOCA), cell adhesion (CD42bCA), cell cycle (G0/G1CA; SCA; G2/MCA), oxidative stress (ROSCA), and necrosis (NCA) network (Fig. 4F, Figs. S3 and S4).

#### 3.1.3 Autophagy and Nuclear Apoptosis

Fig. 5A–E showed nuclear shrinkage by Hoechst 33342 stain and autophagy by acridine orange (AO) stain in BPH and PCa tissue samples.

Benign prostate hyperplasia, adenocarcinomas, and carcinomas tissue samples presented significantly increased pyknotic nuclear expressions than negative control as healthy tissue samples (HH: 54.49 ± 21.04, *p* < 0.01; HAC: 71.19 ± 7.38, *p* < 0.05; HCA: 72.91 ± 9.25 vs. HM: 26.57 ± 3.23, *p* < 0.01; Fig. 5A–C,E). In prostate adenocarcinoma tissue samples (AC), a significantly increased autophagy pattern was observed compared to prostate healthy samples (AOAC: 85.85 ± 8.20 vs. AOM: 52.36 ± 2.72, *p* < 0.05; Fig. 5B,D and Fig. S5).

### 3.2 Cell Extrinsic Factors Involved in EMT Heterogeneity in Benign Prostate Hyperplasia and Prostate Cancer Plasticity

#### 3.2.1 Cell Adhesion

CD42b adhesion glycoprotein (HIP1) levels were presented in Fig. 6A–D using a phycoerythrin (PE) stain, used to assess GPIba expressions in a pro-inflammatory microenvironment. In BPH tissue samples, significantly increased integrin surface glycoprotein levels were observed compared to the negative control (CD42bH: 49.19 ± 8.98 vs. CD42bM: 36.08 ± 9.04, *p* < 0.05, Fig. 6A,D and Fig. S6), sustaining a pro-inflammatory microenvironment as support of the EMT heterogeneity. Increasing CD42b transmembrane glycoprotein expressions were observed in PCa tissue samples compared to healthy tissue samples (CD42bAC: 50.69 ± 19.73, CD42bCA: 9.70 ± 7.00 vs. CD42bM: 36.08 ± 9.04, *p* ≥ 0.05, *p* < 0.01, Fig. 6B–D and Fig. S6), which play a role in acquiring and maintaining the aggressive phenotype of tumoral heterogeneity.

#### 3.2.2 Oxidative Stress

Oxidative stress changes in BPH and PCa tissue samples analyzed by total reactive oxygen species (ROS) flow cytometry methods were presented in Fig. 7A–D.

EMT heterogeneity phenotype is maintained in BPH tissue samples by significantly higher oxidative stress values than in healthy tissue samples (ROSH: 26,460.00 ± 23,449.57 × 10^4^ vs. ROSM: 298.600 ± 612.29 × 10^4^, *p* < 0.01, Fig. 7A,D, and Fig. S7).

Tumoral heterogeneity, an aggressive phenotype, is expressed by deregulated signaling pathways implied in oxidative stress, being highlighted by significantly increased ROS levels in PCa tissue samples compared to controls (ROSAC: 15,350.00 ± 16,388.10 × 10^4^, ROSCA: 18,116.66 ± 26,247.31 × 10^4^ vs. ROSM: 298.600 ± 612.29 × 10^4^, all *p* < 0.05, Fig. 7B–D and Fig. S7).

#### 3.2.3 Microenvironment

The microscopy images were captured from specimens obtained via TURP and biopsy point, as well as from TURP or prostatectomy pieces, being interpreted by two experienced pathologists. From a microscopic evaluation, normal prostate tissue is represented by fibro-muscular stroma, within which acinar structures with lobulated, round-oval architecture are noted, delimited by two rows of cells, cylindrical epithelial cells along the glandular lumen, and a layer of basal cells. The interacinar distance is present and preserved. At the cellular level, the nuclei are small, and the cytoplasm is abundant and eosinophilic. The prostate tissue with benign hyperplasia was microscopically characterized by a glandular and stromal proliferation, with nodular architecture, composed of medium and large glands, with preservation of the two cell rows and aspects of digitiform epithelial papillary extensions or branches. The basal cell layer is present and the interglandular distance is preserved. The nuclei are centrally located, and the nucleoli are not visible. Cases diagnosed with malignant neoplastic proliferation exhibit morphological features in accordance with the degree of differentiation, as reflected in the Gleason score classification.

Benign prostatic hyperplasia (BPH) tissue sections were characterized by a proliferation of both the epithelial component and the fibro-muscular stroma, in varying proportions. The epithelial component consists of large and medium-sized acini arranged in a lobular pattern, with preserved interglandular spacing. The acini are cystically dilated, often containing corpora amylacea within the lumen. An inner layer of columnar to cuboidal epithelial cells, with centrally located nuclei and no visible nucleoli, and an outer layer of flattened, elongated basal cells were observed for each prostate acinus. Stomal proliferation is composed of spindle-shaped cells with round nuclei (Fig. 8A–D). In Fig. 8E–H, BPH sections stained by IHC methods sustain a modified EMT phenotype based on dysregulated cell signaling pathways. Lower S-phase (<7%), arrest in G2/M phase of the cell cycle, and early apoptosis represent independent and predictable factors that sustain EMT heterogeneity in BPH, with good prognostic role in patient survival (Fig. 2D and Fig. 4E).

Well-differentiated adenocarcinomas (Gleason score 6) are constituted by a proliferation of small and medium-sized, crowded, tightly grouped glands, with minimal or absent interglandular distance, absent basal cell layer, and hyperchromic nuclei at the level of epithelial cells. Moderately differentiated adenocarcinomas (Gleason score 7) are represented by fused glandular structures, some with poorly formed lumens, absent interglandular distance. Cribriform nodules, and even round-oval glands with minimal preserved interglandular distance may be present. Moderately differentiated adenocarcinomas, are represented by Gleason score sum 7 (4 + 3), with small proliferated glands, with an infiltrative appearance among the benign-looking glands; Gleason score sum 7 (3 + 4), primary Gleason grade 3 in 60% of neoplastic proliferation, Gleason score sum 8 (4 + 4), with fused glands and cribriform nodules, and Gleason score sum 8 (4 + 4), with the presence of cells with clear cytoplasm arranged in nests, and glandular structures with poorly formed lumens. Poorly differentiated adenocarcinomas (Gleason score 8–10), especially cases with Gleason score 9 or 10, are characterized by the absence of glandular differentiation, with fused acini, cords of tumor cells, isolated malignant neoplastic cells, in signet-ring configuration, and even cribriform nodules centered by comedonecrosis. Gleason score 8 is composed of cribriform nodules or fusion of the glands, with an infiltrative appearance in the prostatic fibro-muscular stroma (Fig. 8I–M).

Poorly differentiated carcinomas, with a Gleason score sum of 8, 9, or 10, display various patterns, ranging from fused small glands without intervening stroma to cribriform glomeruloid patterns and nests of clear cells seen in Gleason pattern 4. Neoplastic proliferation showed no glandular differentiation (Gleason pattern 5), including features such as comedo necrosis, isolated stromal-infiltrative cells, cord-like arrangements, and even signet ring cell morphology (Fig. 8Q–T).

The malignancy diagnosis was confirmed by HMWCK (34BE12 clone) expression to evaluate the basal cell layer, and by AMACR (13H4 clone), a specific biomarker for malignant tumor cells. HMWCK (34BE12 clone) is positive in benign prostatic hyperplasia, in basal cells (Fig. 8G,H), negative in adenocarcinomas (Fig. 8N) and poorly differentiated prostate carcinoma which attests lesion malignancy (Fig. 8S). AMACR is a marker with intense positive expression, with a granular pattern, at the cytoplasmic level, in prostatic epithelial cells from malignant tumor proliferations. Although it is not specific only for prostate cancer, when used together with basal cell markers (high molecular weight cytokeratin-34BE12, p63, CK5/6), it is extremely useful in establishing the malignancy of the examined tissue. It also becomes positive in prostatic atrophy (in which basal cell markers maintain their positive expression) or other benign lesions that mimic malignancy. AMACR (Alpha methylacyl CoA racemase) is a biomarker characterized by granular cytoplasmic expression in acinar epithelial cells, which is positive in adenocarcinoma (Fig. 8O) and prostate atrophy (Fig. 8F), and negative in benign prostatic hyperplasia (Fig. 8E,F) highlighting lesion benignity. The correlation between immunohistochemical aspect and the histopathological aspects on the usual staining, namely the presence of basal cells in the prostatic acini, the interglandular distance, the glandular content (corpora amylacea favor benignity or crystalloids, predominantly in mimicker or malignant lesions) and the nuclear morphology (presence of nuclear atypia, hyperchromasia), are critical in the elaboration of a specific anatomopathological diagnosis. Corpora amylacea are violet or brown eosinophilic material, with a concentric lamellar structure, located in the lumen of the prostatic ducts or acini, present mainly in benign prostatic tissue or on non-cancerous or low-grade cancer prostate specimens. Their presence is less frequently seen in TURP specimens. At the same time, the presence of amylaceous bodies cannot exclude a diagnosis of malignancy. It has also been found that, in adenocarcinomas, crystalloids (eosinophilic material of variable shape and size) appear more frequently in the lumen of proliferating glands, but without absolute specificity. The microscopic diagnosis of prostatic lesions and their classification into a lesional group (benign, malignancy, pseudo neoplastic lesions) is a complex one, which is based on the correlation of morphological parameters, starting with the architecture of the prostatic acini, the interglandular distance, the presence of two rows of acinar-epithelial and basal cells, the intraluminal content (amylose bodies/crystalloids), the appearance of the nuclei and the presence of nuclear atypia. This is complemented, in some cases, by immunohistochemical examination, to confirm the histopathological diagnosis and to perform the differential diagnosis.

Transcription factor p53 (DO-7 clone) is a marker that presents three types of expression: wild type, considered normal (random nuclear positivity), and mutational pattern, either through over-expressed expression in adenocarcinomas (intense nuclear positivity in a proportion of 80%, Fig. 8P) or negative null type, mutational pattern in poorly differentiated prostate carcinoma (Fig. 8T).

Beginning with Fig. 8I, prostate moderately differentiated adenocarcinoma, Gleason score 7 (4 + 3) to Fig. 8T, poorly differentiated prostate carcinoma, Gleason score 9 (4 + 5), deregulated biological mechanisms in PCa samples characterizations sustain an EMT aggressive heterogeneity tumoral phenotype with worse prognostic role of high S-proliferative phase (>12%) and late apoptosis (Fig. 2E and Fig. 4F), being dependent predictive biomarkers of molecular mechanisms network (Supplementary Fig. S8).

## 4 Discussion

### 4.1 Cell Extrinsic Factors Involved in EMT Heterogeneity in Benign Prostate Hyperplasia and Prostate Cancer Plasticity

#### 4.1.1 Cell Cycle

Establishing the prognostic roles of new checkpoints as predictive biomarkers in the context of deregulated cell signaling pathways and EMT heterogeneity in BPH and PCa represents a novel step toward the discovery of therapeutic resources. In our previous report, we observed that EMT aggressive heterogeneity limits treatment response. The high S-proliferative phase is an unfavorable prognostic biomarker and a dependent predictor of dysregulated molecular mechanisms, including late apoptosis and necrosis (caspase-3/7 activity) and cell growth inhibition (G2/M phase blockage) in BPH cell cultures with a heterogeneous aggressive phenotype [6]. In the present study, cell growth inhibition, achieved through G2/M phase cell cycle arrest, with a low S-phase category (<7%), was observed in BPH tissue samples. The heterogeneous aggressive phenotype of adenocarcinomas was characterized by two distinct deregulated cell cycle patterns, either G0/G1 or G2/M phase blockages, a high S-phase category (>12%), and an apoptosis-necrosis continuum, contributing to tumoral aggressiveness. Advanced prostate cancer tissue samples (carcinomas) present an uncontrolled S-phase proliferation with higher tumoral aggressiveness. Furthermore, in BPH tissue samples, the low S-proliferative phase category (SH: <7%) is an independent predictor, serving as a favorable prognostic biomarker for patient survival, and is associated with dysregulated molecular signaling pathways that characterize EMT heterogeneity phenotype. Dysregulated cell cycle, early, late apoptosis, nuclear shrinkage, autophagy, cell adhesion, and oxidative stress contribute to maintaining the EMT heterogeneity phenotype, highlighting the importance of the low S-phase category as a favorable prognostic biomarker. In PCa samples, the high S-proliferative phase category (SCA, >12%) is an unfavorable prognostic biomarker for patient survival, characteristically associated with an aggressive tumoral heterogeneity phenotype. The high S-phase category has more than 50% higher risk of death or recurrence (worse prognostic), being a dependent predictor factor for dysregulated molecular mechanisms network represented by cell cycle blockage in G0/G1 or G2/M phases, late apoptosis, necrosis, autophagy, cell adhesion, and oxidative stress, characterizing the aggressive phenotype of tumoral heterogeneity.

EMT heterogeneity is involved in initiation, progression, metastasis, and resistance to therapy in various cancer types [32]. Cancer stem cells (CSCs) are responsible for tumor development, leading to intratumor heterogeneity, metastasis, and therapeutic resistance [1,33]. The plasticity of cancer stem cells represents a dynamic state of gain or loss [34,35]. Breast [34,36], glioblastoma [37,38], and melanoma [39,40] exhibit cancer stem cell plasticity that is regulated by cell-intrinsic factors, such as genetic/epigenetic changes, and cell-extrinsic factors, including oxidative stress and the microenvironment [34,41]. EMT heterogeneity involves changes in the molecular, morphological, and functional aspects of epithelial cells, transforming them into mesenchymal cells, which can occur during metastasis or drug resistance [42]. EMT aggressive heterogeneity sustains cell plasticity in carcinomas [43].

Lineage plasticity in different cancer types is regulated by cell-intrinsic factors, including loss of the retinoblastoma protein (RB1), phosphatase and tensin homolog (PTEN), and TP53 tumor suppressor genes [44]. Trp53 and Rb1 determine plasticity and transdifferentiation in prostate and lung cancers [1,45]. DNA methylation and chromatin remodeling are epigenetic changes that play essential roles in lineage plasticity, induced during tumor progression [32,41,46,47,48]. In cell cycle regulation, changes in cyclin expression determine cyclin-dependent kinase (CDK) activation. In the G0/G1 phase, cyclin D activates CDK4/6, leading to the phosphorylation of the retinoblastoma tumor suppressor protein (Rb) [49,50,51]. Entry into mitosis is marked by cyclin B-CDK1 interactions, which play a role in RB inhibition via phosphorylation during the S to G2 and G2 to M transitions. Cyclin B expression during the G2 phase activates CDK1 for chromosome condensation and nuclear envelope breakdown [52,53]. Cell cycle regulators represent targets in cancer therapies. Rb, p53, and p21 proteins are involved in cell cycle regulation [54,55,56,57,58]. Rb controls G1 to S phase transition. Rb phosphorylation is induced by cyclin D-CDK4/6 and cyclin E-CDK2 complexes [59]. The p53 protein, a tumor suppressor and pleotropic transcription factor, represents a checkpoint resulting from TP53 gene expression. It receives signals from oxidative stress and DNA damage pathways, activating genes with representative functions in cell cycle blockage. In cells, if DNA damage repair pathways fail, p53 induces apoptosis, mediated by the Bcl-2 family [60]. In our study, transcription factor p53 is a marker that presents three types of expression: wild type, considered normal (random nuclear positivity), and mutational pattern in PCa tissue samples, either through over-expression (intense nuclear positivity in a proportion of 80%, adenocarcinomas) or negative null type (carcinomas). TP53 mutations are late events in malignancy progression; they are associated with metastatic spread and the development of castration resistance. As a response to DNA damage, p21, induced by the p53 tumor suppressor protein-deregulated pathway, determines cell cycle arrest in the G0/G1 and G2/M phases [61]. By binding to cyclin D-CDK4/6 and E-CDK2 complexes, p21 inhibits Rb phosphorylation in the G0/G1 phase [62]. In the S-proliferative phase, p21 plays an essential role by inhibiting the cyclin A-CDK1 complex and halting the cell cycle at the G2/M transition [63]. Upregulated P21 expression is correlated with prostate cancer aggressiveness, but decreased P21 values act against prostate tumorigenesis [64].

#### 4.1.2 Apoptosis and Necrosis-Caspase-3/7 Activity

Cell death is induced by necrosis, mitotic catastrophe, and autophagy [65]. Apoptosis regulators represent targets for cancer therapeutic strategies. Apoptosis is characterized by morphological changes, including plasma membrane blebbing, nuclear shrinkage, and appearance of apoptotic bodies [66]. In our present study, caspases 3/7 activity analysis highlighted biochemical events involved in the pro-apoptotic signal (EA) in BPH and PCa tissue samples. An inflammatory response was observed as a response following injury induction and oxidative stress, leading to dysregulated late apoptosis (LA) patterns in BPH and PCa, and apoptosis—necrosis continuum in adenocarcinomas. Furthermore, in BPH tissue samples, increased early apoptosis as measured by caspase 3/7 activity, represents an independent significant predictor biomarker for dysregulated biological mechanisms involved in EMT heterogeneity, including autophagy, cell cycle, nuclear shrinkage, and oxidative stress, with a favorable prognostic role in patient survival. In PCa tissue samples, increased late apoptosis levels as measured by caspase 3/7 analysis represent a checkpoint and serve as a dependent predictor biomarker with a worse prognosis for patient survival rate, characterized by aggressive tumoral plasticity that involve dysregulated biological mechanisms such as autophagy, cell adhesion, cell cycle, oxidative stress, and necrosis. In a previous study, in heterogeneous aggressive phenotype BPH cell cultures, increased late apoptosis status via caspases-3/7 biochemical cascade represents an unfavorable prognostic biomarker and a dependent predictor factor of dysregulated molecular mechanisms network including cell adhesion, G2/M phase cell cycle blockage, and oxidative stress [6].

Extrinsic and intrinsic signaling pathways induce apoptosis. By the death-receptor-mediated extrinsic pathway, ligands bind to plasma membrane death receptors, activating initiator caspase 8 [67]. In cells, the extrinsic pathway activates effector caspase-3/7, whereas in cancer cells, caspase-8 amplifies the intrinsic death-signaling pathway. Pro- and anti-apoptotic Bcl-2 family proteins induce changes in protein mitochondrial surface interactions, determine cytochrome c release, and activate the apoptosome complex, formed by initiator caspase-9 and effector caspases-3/7. Cleaved cytokeratins (CK) by caspases-3/7 activate endonucleases that generate nucleosome DNA (nDNA), apoptotic bodies formation, and phosphatidylserine exposure on the external surface of the plasma membrane [68]. The apoptosis hallmark is DNA fragmentation whereas necrotic cells exhibit less DNA fragmentation. Apoptotic cells display a DNA content below the G0/G1 phase of the cell cycle, as indicated by a 2N DNA peak in viable cells by flow cytometry. A DNA-intercalating dye, propidium iodide (PI), binds to nucleic acids, producing a red fluorescence emission proportional to DNA content, which appears below the G0/G1 peak on the DNA histogram, as the sub-G0/G1 phase [69]. Caspase-3/7 activity is a reliable method for distinguishing between apoptosis and most forms of necrosis, as determined by DEVD-MR and propidium iodide (PI) using flow cytometry to distinguish viable from dead cells. Phagocytes remove damage-associated molecular patterns (DAMPs) that provoke inflammation after changes in the phosphatidylserine signal on the surface of apoptotic cells [69].

High concentrations of toxins, cytotoxic drugs, reactive oxygen species, extreme temperatures, and physical injury initiate necrosis. Necrosis involves plasma membrane damage under severe conditions, leading to its permeabilization. Water influx and intracellular potassium efflux trigger plasma membrane rupture, releasing intracellular contents into the extracellular space. Necrosis occurs within 2–3 h after exposure to a necrosis-inducing stimulus, whereas apoptosis occurs asynchronously within 8–20 h. Necrosis is an inflammatory cell death pathway, as it involves pro-inflammatory molecules [69,70,71]. Apoptosis initiated by extrinsic signaling pathways involves the tumor necrosis factor (TNF) receptor gene superfamily [72].

Ligands and their death receptors, FasL/FasR, TNF-α/TNFR1, Apo3L/DR3, Apo2L/DR4, and Apo2L/DR5 [73,74], play roles in transmitting apoptosis signals from the cell surface to the intracellular by signaling pathways. Mitochondrial events initiate the apoptotic intrinsic signaling pathways. Radiation, toxins, hypoxia, viral infections, and free radicals, all contribute to apoptosis. Apoptotic stimuli induce changes in the inner mitochondrial membrane and loss of the mitochondrial transmembrane potential. Pro-apoptotic proteins, cytochrome c, second mitochondria-derived activator of caspase/direct inhibitor of apoptosis-binding protein with low pI (Smac/DIABLO), and serine protease HtrA2/Omi, are released from the intermembranar space into the cytosol [75,76,77]. Pro-apoptotic proteins bind cytochrome c, activate Apoptotic Peptidase Activating Factor 1 (Apaf-1) as a pro-caspase-9 and form an apoptosome [78], thereby determining caspase-9 activation. Smac/DIABLO and HtrA2/Omi inhibit IAP (inhibitors of apoptosis proteins) activity [79], promoting apoptosis. Endonuclease G participates in chromatin cleavage and produces oligonucleosomal DNA fragments [80]. Cleaved CAD by caspase-3 determines oligonucleosomal DNA fragmentation and chromatin condensation. The Bcl-2 protein family regulates mitochondrial apoptotic events [81]. The transcription factor p53 plays a crucial role in regulating the expression of the Bcl-2 protein family [82]. The Bcl-2 protein family regulates mitochondrial membrane permeability, inducing apoptosis or preventing it.

Effector caspases-3/7 activation initiates apoptosis. Caspases-3/7 activate cytoplasmic endonucleases, which play a role in the degradation of nuclear and cytoskeletal proteins. Caspase-3, caspase-6, and caspase-7 cleave cytokeratins, leading to morphological and biochemical changes in apoptotic cells [83]. Caspase-3 is activated by initiator caspases such as caspase-8, caspase-9, or caspase-10. Caspase-3/7 determines endonuclease CAD activation. In tumoral cells, CAD is complexed with its inhibitor, ICAD. In apoptotic cells, caspase-3/7 cleaves ICAD, releasing CAD, which plays roles in DNA degradation, chromatin condensation, and cell disintegration into apoptotic bodies [15]. Caspase-3/7 expression changes drive cancer progression by altering the homeostatic balance between cell death and proliferation. The initiator caspase cleaves and activates the executioner caspases-3/7. Caspase-3 expression loss contributes to tumoral cell evasion by apoptosis. Reduced caspase-3 expression is correlated with poor prognosis and therapy resistance in human tumors, including esophageal [84], non–small cell lung carcinomas [85], and childhood medulloblastoma [86]. Caspase-3 activity inhibitors, such as Bcl-2 and Bax proteins [87], are observed in prostatic diseases. Caspase-3/7 expression in different cancer types was correlated with clinical features [88,89,90,91,92,93,94]. Reduced caspase-3 expression was reported in prostate cancer [95,96]. Cancerous epithelium exhibits deregulated apoptotic signals from death receptor-mediated and intrinsic apoptotic pathways, compromising apoptosis. Caspase activity loss is a valuable marker for PCa diagnosis, as it triggers apoptosis, and serves as a target for PCa treatment [97].

#### 4.1.3 Autophagy and Nuclear Apoptosis

Apoptosis and autophagy are associated with biological processes because mitochondria serve as central organelles for both. Common signaling pathways involved in apoptosis and autophagy, include protein kinase B, p70S6 kinase, Death-associated protein kinase 1 (DAPk), Beclin 1, BNIP3, HSpin1, and protymosin-α [98]. Beclin-1 cleavage activates mitochondrial apoptosis pathway by inhibiting autophagy and inducing apoptosis [99]. Additionally, in BPH cell cultures, Beclin-1 cleavage increases apoptosis and decreases autophagy levels after 20 h of incubation [100].

Autophagy is involved in adaptive and innate immune systems, degrades intracellular pathogens, recycles cellular components, and destroys neoplastic lesions [101,102]. In macroautophagy, autophagosomes fuse with lysosomes to form autophagolysosomes which exhibit protease activity. In microautophagy, small parts of organelles or the cytosol fuse with lysosomes [103]. Autophagy protects healthy and cancer cells from chemotherapy aggression [104]. In the present study, in the prostate benign hyperplasia, adenocarcinomas, and carcinomas were observed increased pyknotic nuclear expressions. An increased autophagy pattern was observed in prostate adenocarcinoma tissue samples. Our previous report in heterogeneous aggressive BPH cell cultures showed an apoptosis program, characterized by increasing nuclear shrinkage and lysosomal activity, reported to healthy cell cultures, positively correlated with necrosis and the S phase of the cell cycle [6]. *In vivo*, cells that are undergoing autophagy are phagocytized by neighboring cells. Apoptotic cells secrete chemotactic factors, determining a local accumulation of macrophages [105,106]. In tumor cells, the activated necrosis signaling pathway drives the release of pro-inflammatory molecules at the inflammatory site [107,108]. In contrast, the apoptosis signaling pathway, through the presence of by apoptotic bodies suppresses the production of pro-inflammatory mediators by activated macrophages. A critical component of the apoptosis and autophagy signaling pathways network is their anti-inflammatory outcome. DNA fragmentation and apoptotic body formation occur via distinct biological processes depending on the caspase-3/7 biochemical cascade [109].

### 4.2 Cell Extrinsic Factors Involved in EMT Heterogeneity in Benign Prostate Hyperplasia and Prostate Cancer Plasticity

#### 4.2.1 Cell Adhesion

Cell-extrinsic factors, including inflammation, microenvironment, and therapeutic stress, induce cell plasticity [110]. Chronic inflammation is a hallmark of cancer [111]. Lineage plasticity caused by inflammation is represented by metaplasia [112]. Injury and chronic inflammation in healthy tissues induce metaplasia by transdifferentiation mechanisms [113,114]. Pro-inflammatory cytokines and inflammation-associated myeloid cells are correlated with EMT heterogeneity in breast cancer plasticity [115]. The tumor microenvironment (TME), fibroblasts, macrophages, endothelial cells, and infiltrating immune cells interact with tumor cells, promoting tumor plasticity [116]. Tumor-associated macrophages (TAMs) facilitate tumor progression and metastases. Stromal cells and extracellular matrix components (ECM) are involved in cellular plasticity [117]. BPH is characterized by stromal and epithelial hyperplasia, and nodular formation. Prostate intraepithelial neoplasia (PIN) and prostate cancer (PCa) primarily arise from epithelial cells localized in the prostate gland [118,119]. Growth factors and cytokines deregulate the proliferation/apoptosis ratio deregulation in prostate epithelial and stromal cells [120].

In the present study, increased integrin surface glycoprotein levels in BPH tissue samples support the conversion from a pro-inflammatory microenvironment to an epithelial-mesenchymal transition phenotype. Increased CD42b transmembrane glycoprotein expression in PCa tissue samples plays an essential role in the acquisition and maintenance of tumoral heterogeneity and the aggressive phenotype. In a previous study, increased CD42b+ transmembrane glycoprotein levels in prostate hyperplasia heterogeneous primary cell cultures were associated with platelet and lymphocyte recruitment to the inflammatory site. Cell adhesion, as measured by CD42b+ glycoprotein expression, was negatively correlated with the G0/G1 phase of the cell cycle and with viability [6].

EMT represents the loss of epithelial cell characteristics and the acquisition of mesenchymal phenotype, and is associated with metastasis and tumor invasion, in prostate cancer [121], and breast cancer [122]. EMT heterogeneity is present in BPH [123]. Dysregulated proliferation, apoptosis, and oxidative stress [120,124,125] indicate EMT involvement in BPH development. Inflammation contributes to BPH development. When epithelial and stromal cells are damaged by external stimuli, T lymphocytes, macrophages, B lymphocytes, and cytokines promote the fibromuscular remodeling process [126]. Integrins regulate apoptosis through Fas-Fas-L signaling pathways: 1. Directly decreasing Fas expression or reducing FLIP expression to facilitate ECM binding; 2. Integrin detachment from ECM initiates cell death by the Fas-Fas-L pathway [127]. Integrin mediates cell adhesion to ECM, regulates CyclinD1, CyclinE-cdk2, and Rb protein activities, determining mitosis. In cells, integrin detachment from the ECM determines cell cycle blockage in the G0/G1 phase and apoptosis. *In vitro* prostate cell cultures, integrin α2β1 and collagen-I activate the MAPK kinase 7 (MAPKK7) pathway, determining cell proliferation and EMT. BTT-3033 inhibits MAPKK7 phosphorylation, leading to apoptosis and G0/G1 phase cell cycle arrest [128,129].

#### 4.2.2 Oxidative Stress

A dysregulated pro-oxidant/antioxidant ratio, inactivation of antioxidant enzymes, and excessive antioxidant consumption represent key factors in oxidative stress [130]. In cancer cells, elevated reactive oxygen species (ROS) levels lead to DNA damage, which in turn blocks cell cycle [131].

In the present study, EMT heterogeneity phenotype in BPH tissue samples is maintained by increased oxidative stress. In PCa tissue samples, tumoral aggressive heterogeneity phenotypes show deregulated signaling pathway expression by significantly increased ROS levels. Reactive oxygen species induce carcinogenesis by hydroxyl radical attack on DNA, forming 8-hydroxydeoxyguanosine (8-OH-dG) [132] with a highly mutagenic effect due to GC to TA transversions [133]. In cancer cells, increased oxidative stress leads to DNA damage, followed by cell cycle arrest [134]. DNA damage represents a valuable biomarker in treatment monitoring [135]. Higher ROS levels inhibit tumor cell growth by activating caspases and kinases [136]. Decreased caspase-3 levels were observed in prostate cancer patients reported to benign prostate hyperplasia patients. Dysregulated caspase-3/7 expression represents a biomarker in prostate cancer diagnosis [131]. Necrosis predicts poor prognoses in metastatic breast cancer, non-small cell lung cancer [137], malignant mesothelioma [138], clear cell renal cell carcinoma [139], malignant gastrointestinal stromal tumors [140], and endometrial cancer [141]. Necrosis correlated with tumor size, stage, and aggressiveness [142,143]. Necrosis is characterized by compromised plasma membrane integrity, cellular organelle swelling, DNA degradation, and the uncontrolled release of pro-inflammatory molecules in cells [21].

Metabolic stress alters mitochondrial membrane potential, triggering apoptosis [20]. Glucose deprivation induces metabolic stress, leading to a metabolic catastrophe, apoptosis, or necrosis in cancer cells with TP53 mutations [144]. Intracellular ROS accumulation determines apoptosis by metabolic stress. Low hydrogen peroxide (H_2_O_2_) and super-oxide (O_2_^−^) levels promote tumoral proliferation, whereas higher ROS concentrations induce apoptosis [145]. In chronic inflammation, hypoxia-inducible factor 1α (HIF1α) favors cancer progression [146,147].

#### 4.2.3 Microenvironment

Tumoral microenvironment induces apoptosis. Necrosis triggers local and systemic pro-inflammatory responses, thereby shortening the patient’s survival. In dysregulated chronic inflammation, exposure to pro-inflammatory cytokines (IL-1, IL-6, and TNF-α) in a continuous way enhances the recruitment of pro-inflammatory immune cells, leading to cachexia and multi-organ deterioration [148].

Tumor-infiltrating immune cells, macrophages, regulatory T cells, and neutrophils determine ROS release in the tumoral microenvironment. Lower ROS levels are required for T lymphocyte activation. Increased ROS levels inhibit T cells with antitumoral function [149]. Increased tumor-associated glycolysis reduces cytotoxic T cells ability to kill tumors. Tumoral cells recruit immune cells, including macrophages, neutrophils, and regulatory T cells to create an immunosuppressive microenvironment [150].

By flow cytometry and IHC analysis, new checkpoints with prognostic roles, involved in dysregulated cell signaling pathways, sustain an adaptive microenvironment, characteristically associated with EMT heterogeneity and immune evasion. Prostate adenocarcinomas present an apoptosis-necrosis continuum as a specific adaptation to the EMT-aggressive heterogeneity phenotype involved in cell plasticity. Low and high S-proliferative categories, G0/G1 or G2/M-arresting phases, and early and late apoptosis represent predictable biomarkers with personalized therapeutic importance in prostate EMT heterogeneity. New possibilities for adapting therapeutic findings by targeting tumoral cells based on epigenetics, microenvironmental heterogeneity, and metabolic switches on-off to utilize preferentially substrate resources, primarily glycolysis, when cell signaling pathways are completely deregulated, are required.

An essential limitation of this study was variability of the EMT-aggressive heterogeneity phenotype that sustains an imuno-adaptive microenvironment involved in cell plasticity. To extend our laboratory findings, future research directions will focus on peripheral immune tolerance mediated by CD25^+^FOXP3^+^CD4^+^ regulatory lymphocytes (Tregs), and FOXP3^+^/CD8a^+^ involved in prostate cancer progression.

## 5 Conclusion

Epithelial-mesenchymal transition (EMT) heterogeneity represents a regulator of cell plasticity. Low and high S-proliferative phases of the cell cycle, early, and late apoptosis as new checkpoints involved in EMT heterogeneity, are reported to dysregulate cell signaling pathways, serving as predictor biomarkers for BPH and PCa patient survival, and are useful in targeting personalized cancer therapy development.

## Data Availability

Data and their interpretation are contained in the research article.

## Supplementary Materials

The supplementary material is available online at https://www.techscience.com/doi/10.32604/or.2026.072421/s1. Supplementary Figure S1: Cell cycle signaling pathway by propidium iodide (PI) stain: S1A1–3; S2A1–3; S4A1–3; S15A1–3; S5B1–3; S6B1–3; S10B1–3; S22B1–3; S13C1–3; S14C1–3; S16C1–3; S31C1–3. Supplementary Figure S2: Cell cycle phase statistics in BPH and PCa: A–C; Predictor factors represented by coefficient of multiple regression (R2) in BPH and PCa tissue samples: D,E. Supplementary Figure S3: Caspases-3/7 activity pathway highlighted by DEVD-MR/PI stain, apoptotic signal by DEVD-MR stain, and cell permeability by PI stain: S1A1–3; S2A1–3; S4A1–3; S15A1–3; S5B1–3; S6B1–3; S10B1–3; S22B1–3; S13C1–3; S14C1–3; S16C1–3; S31C1–3. Supplementary Figure S4: Caspases-3/7 pattern statistics in BPH and PCa: A–D; Predictor factors represented by coefficient of multiple regression (R2) in BPH and PCa tissue samples: E,F. Supplementary Figure S5: Autophagy and nuclear apoptosis (H^+^AO^+^) expressions by acridine orange/Hoechst stain to highlight nuclear shrinkage (H^+^) and lysosomal activity (AO^+^): S1A1–3; S2A1–3; S4A1–3; S15A1–3; S5B1–3; S6B1–3; S10B1–3; S22B1–3; S13C1–3; S14C1–3; S16C1–3; S31C1–3. Autophagy and nuclear apoptosis pattern statistics: D,E. Supplementary Figure S6: Cell adhesion (A) pathway highlighted by integrin surface glycoprotein conjugated with phycoerythrin (CD42b-PE) stain: S1A1; S1A2; S2A1; S2A2; S4A1; S4A2; S15A1; S15A2; S5B1; S5B2; S6B1; S6B2; S10B1; S10B2; S22B1; S22B2; S13C1; S13C2; S14C1; S14C2; S16C1; S16C2; S31C1; S31C2. Cell adhesion by integrin surface glycoprotein pattern statistics: 6D. Supplementary Figure S7: Oxidative stress pathway highlighted by reactive oxygen species (ROS) stain: S1A1; S1A2; S2A1; S2A2; S4A1; S4A2; S15A1; S15A2; S5B1; S5B2; S6B1; S6B2; S10B1; S10B2; S22B1; S22B2; S13C1; S13C2; S14C1; S14C2; S16C1; S16C2; S31C1; S31C2. Oxidative stress by ROS statistics: D. Supplementary Figure S8: Deregulated cellular-phenotypic plasticity in BPH and PCa tissue sections. Microenvironment characterization by Hematoxylin-eosin (H&E) stain, HMWCK (34BE12 clone) biomarker to highlight the tumoral basal cell layer, AMACR (13H4 clone) biomarker to express tumoral cells, and p53 (DO-7 clone) to evaluate nuclear expression: A–T.

## Abbreviations

EMT	epithelial-mesenchymal transition
BPH	benign prostate hyperplasia
PCa	prostate cancer
PI	propidium iodide
DEVD-MR	DEVDase enzyme activity by Magic Red stain
CD42b-PE	GPIba platelet glycoprotein conjugated with phycoerythrin
ROS	total reactive oxygen species
IHC	immunohistochemistry
FasL	type-II transmembrane protein in the tumor necrosis factor
ECM	extracellular matrix
PI3K	phosphatidylinositol 3-kinase
AKT	Protein Kinase B
DHT	Dihydrotestosterone
TURP	transurethral resection of the prostate
H&E	Hematoxylin-eosin
HMWCK	Cytokeratin HMW
AMACR	Alpha methylacyl CoA racemase
RB	retinoblastoma protein
PTEN	phosphatase and tensin homolog
CDK	cyclin-dependent kinase
Rb	retinoblastoma tumor suppressor protein
CKs	Cleaved cytokeratins
nDNA	nucleosome DNA
PD	pharmacodynamics
CAD	Caspase-activated endonucleases
DAMPs	damage-associated molecular patterns
TNFR1	Tumor necrosis factor receptor 1
DR	Death Receptor
Apo3L	ligand for the death-domain-containing receptor Apo3
Smac/DIABLO	Second mitochondria-derived activator of caspase/direct inhibitor of apoptosis-binding protein with low pI
HtrA2/Omi	serine protease HTRA2, mitochondrial
Apaf-1	Apoptotic Peptidase Activating Factor 1
IAP	inhibitors of apoptosis proteins
AIF	Apoptosis-inducing factor
CAD	carbamoyl-phosphate synthetase 2, aspartate transcarbamylase, and dihydroorotase; DAPk-Death-associated protein kinase 1
BNIP3	BCL2/adenovirus E1B 19 kDa protein-interacting protein 3
LNCaP	androgen-sensitive human prostate adenocarcinoma cells
PWR-1E	cells derived from non-neoplastic adult human prostate cells that were infected with the Ad12-SV40 virus
TME	tumor microenvironment
TAMs	Tumor-associated macrophages
PIN	Prostate intraepithelial neoplasia
MAPKK7	MAPK kinase 7
8-OH-dG	8-hydroxyguanine
HIF1α	hypoxia-inducible factor 1α
