# Supplementary figures and images for: Dysregulated Cell Signaling Pathways in Prostate Tumoral Plasticity—Checkpoints (part 2 of 2)
Source: Oncol Res. 2026 May 21;34(6):16. doi: 10.32604/or.2026.072421 (PMC13227601; doi:10.32604/or.2026.072421)

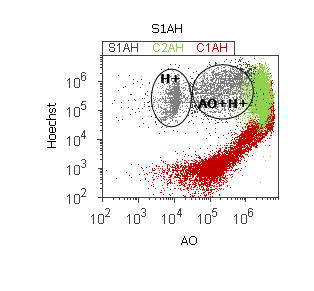

Supplement: Supplementary file 1 [file OncolRes-34-72421-s001.zip › Figure_S5/Figure_S5_S1A1.tiff]

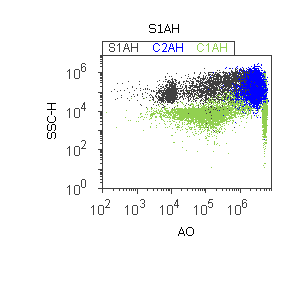

Supplement: Supplementary file 1 [file OncolRes-34-72421-s001.zip › Figure_S5/Figure_S5_S1A2.tiff]

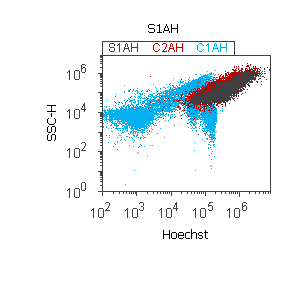

Supplement: Supplementary file 1 [file OncolRes-34-72421-s001.zip › Figure_S5/Figure_S5_S1A3.tiff]

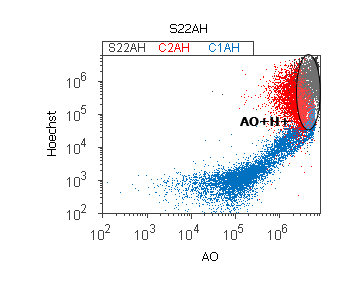

Supplement: Supplementary file 1 [file OncolRes-34-72421-s001.zip › Figure_S5/Figure_S5_S22B1.tiff]

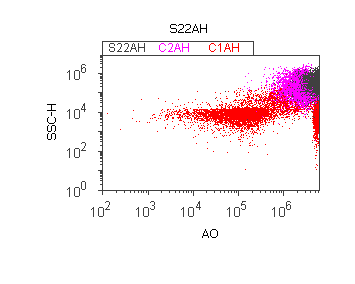

Supplement: Supplementary file 1 [file OncolRes-34-72421-s001.zip › Figure_S5/Figure_S5_S22B2.tiff]

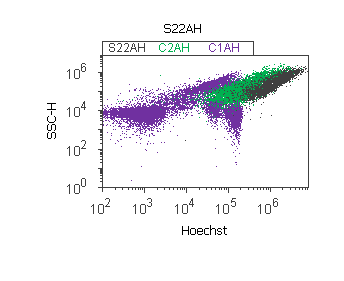

Supplement: Supplementary file 1 [file OncolRes-34-72421-s001.zip › Figure_S5/Figure_S5_S22B3.tiff]

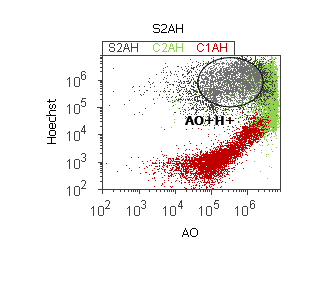

Supplement: Supplementary file 1 [file OncolRes-34-72421-s001.zip › Figure_S5/Figure_S5_S2A1.tiff]

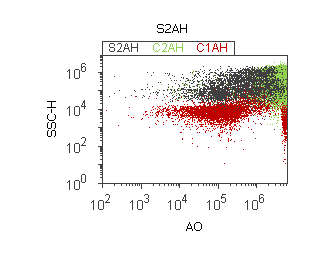

Supplement: Supplementary file 1 [file OncolRes-34-72421-s001.zip › Figure_S5/Figure_S5_S2A2.tiff]

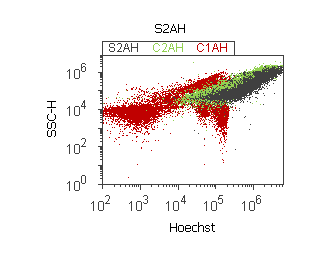

Supplement: Supplementary file 1 [file OncolRes-34-72421-s001.zip › Figure_S5/Figure_S5_S2A3.tiff]

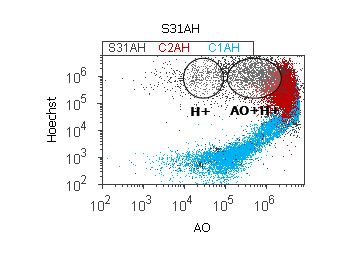

Supplement: Supplementary file 1 [file OncolRes-34-72421-s001.zip › Figure_S5/Figure_S5_S31C1.tiff]

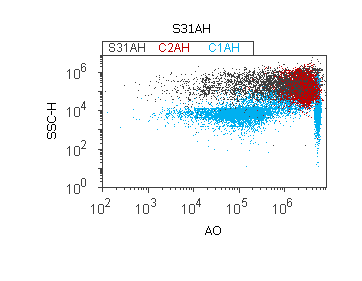

Supplement: Supplementary file 1 [file OncolRes-34-72421-s001.zip › Figure_S5/Figure_S5_S31C2.tiff]

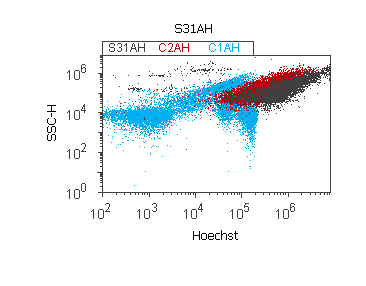

Supplement: Supplementary file 1 [file OncolRes-34-72421-s001.zip › Figure_S5/Figure_S5_S31C3.tiff]

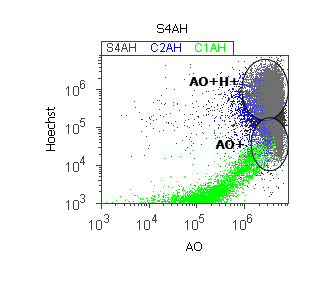

Supplement: Supplementary file 1 [file OncolRes-34-72421-s001.zip › Figure_S5/Figure_S5_S4A1.tiff]

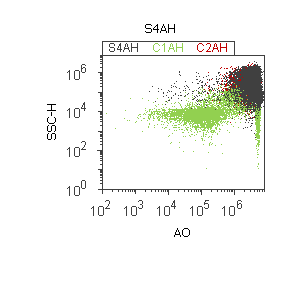

Supplement: Supplementary file 1 [file OncolRes-34-72421-s001.zip › Figure_S5/Figure_S5_S4A2.tiff]

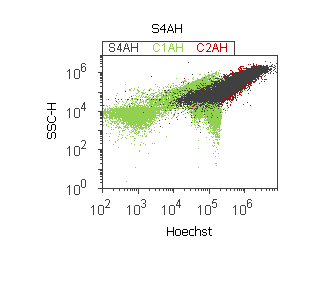

Supplement: Supplementary file 1 [file OncolRes-34-72421-s001.zip › Figure_S5/Figure_S5_S4A3.tiff]

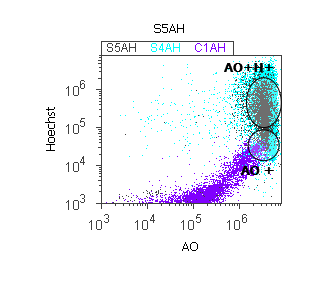

Supplement: Supplementary file 1 [file OncolRes-34-72421-s001.zip › Figure_S5/Figure_S5_S5B1.tiff]

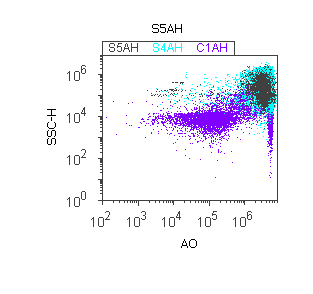

Supplement: Supplementary file 1 [file OncolRes-34-72421-s001.zip › Figure_S5/Figure_S5_S5B2.tiff]

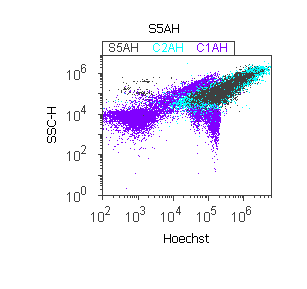

Supplement: Supplementary file 1 [file OncolRes-34-72421-s001.zip › Figure_S5/Figure_S5_S5B3.tiff]

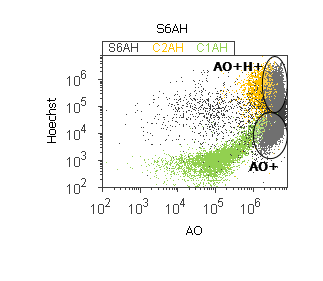

Supplement: Supplementary file 1 [file OncolRes-34-72421-s001.zip › Figure_S5/Figure_S5_S6B1.tiff]

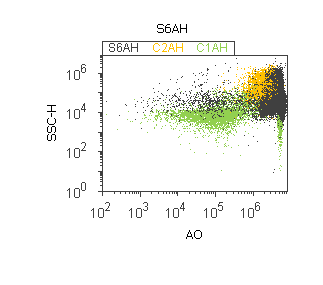

Supplement: Supplementary file 1 [file OncolRes-34-72421-s001.zip › Figure_S5/Figure_S5_S6B2.tiff]

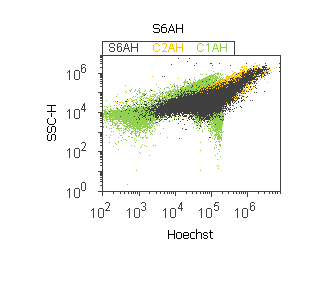

Supplement: Supplementary file 1 [file OncolRes-34-72421-s001.zip › Figure_S5/Figure_S5_S6B3.tiff]

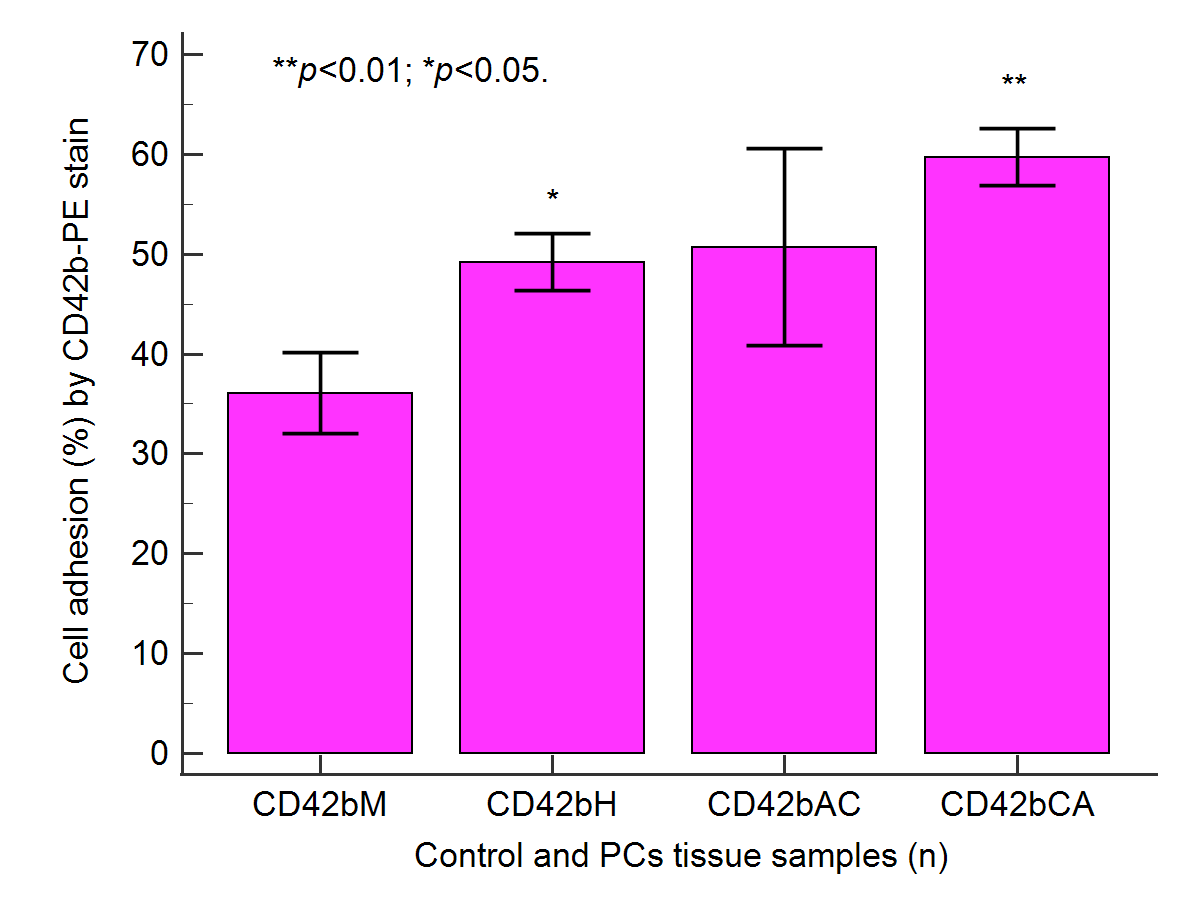

Supplement: Supplementary file 1 [file OncolRes-34-72421-s001.zip › Figure_S6/Figure_S6D.tif]

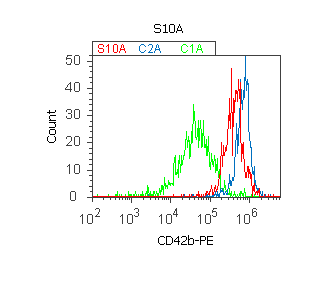

Supplement: Supplementary file 1 [file OncolRes-34-72421-s001.zip › Figure_S6/Figure_S6_S10B1.tiff]

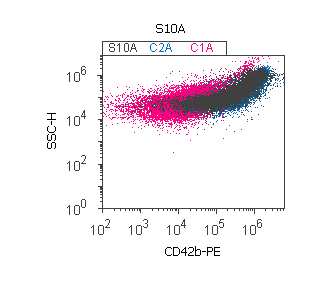

Supplement: Supplementary file 1 [file OncolRes-34-72421-s001.zip › Figure_S6/Figure_S6_S10B2.tiff]

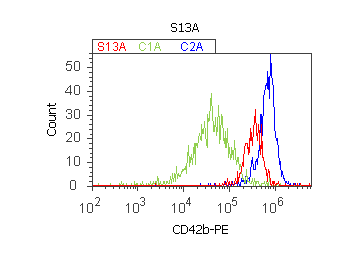

Supplement: Supplementary file 1 [file OncolRes-34-72421-s001.zip › Figure_S6/Figure_S6_S13C1.tiff]

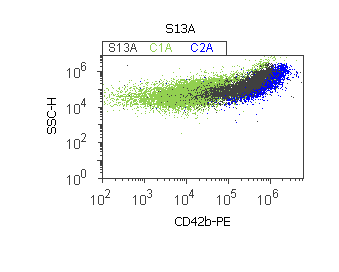

Supplement: Supplementary file 1 [file OncolRes-34-72421-s001.zip › Figure_S6/Figure_S6_S13C2.tiff]

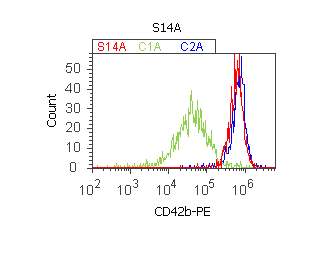

Supplement: Supplementary file 1 [file OncolRes-34-72421-s001.zip › Figure_S6/Figure_S6_S14C1.tiff]

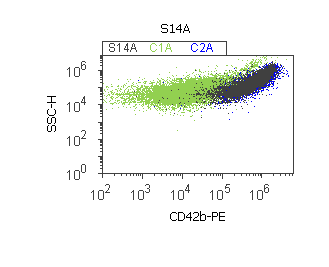

Supplement: Supplementary file 1 [file OncolRes-34-72421-s001.zip › Figure_S6/Figure_S6_S14C2.tiff]

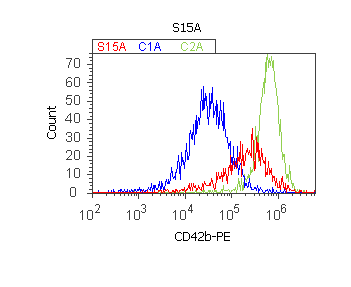

Supplement: Supplementary file 1 [file OncolRes-34-72421-s001.zip › Figure_S6/Figure_S6_S15A1.tiff]

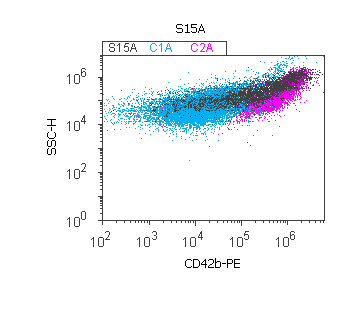

Supplement: Supplementary file 1 [file OncolRes-34-72421-s001.zip › Figure_S6/Figure_S6_S15A2.tiff]

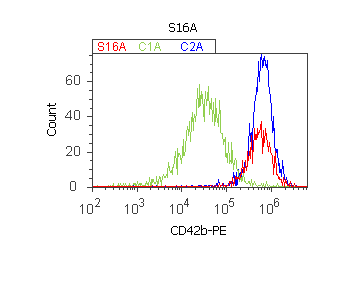

Supplement: Supplementary file 1 [file OncolRes-34-72421-s001.zip › Figure_S6/Figure_S6_S16C1.tiff]

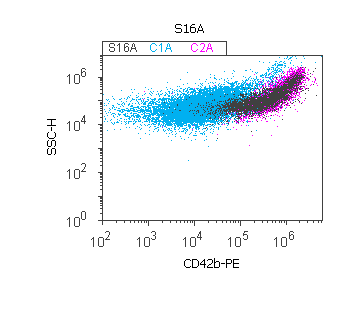

Supplement: Supplementary file 1 [file OncolRes-34-72421-s001.zip › Figure_S6/Figure_S6_S16C2.tiff]

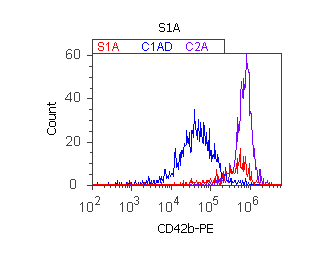

Supplement: Supplementary file 1 [file OncolRes-34-72421-s001.zip › Figure_S6/Figure_S6_S1A1.tiff]

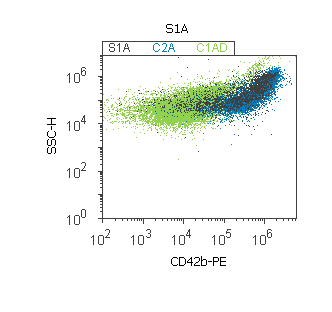

Supplement: Supplementary file 1 [file OncolRes-34-72421-s001.zip › Figure_S6/Figure_S6_S1A2.tiff]

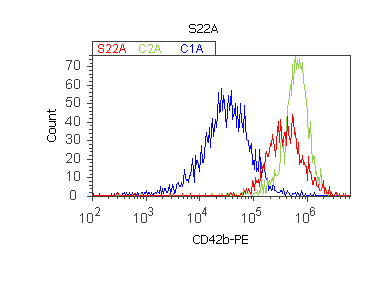

Supplement: Supplementary file 1 [file OncolRes-34-72421-s001.zip › Figure_S6/Figure_S6_S22B1.tiff]

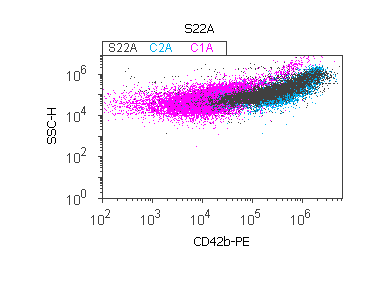

Supplement: Supplementary file 1 [file OncolRes-34-72421-s001.zip › Figure_S6/Figure_S6_S22B2.tiff]

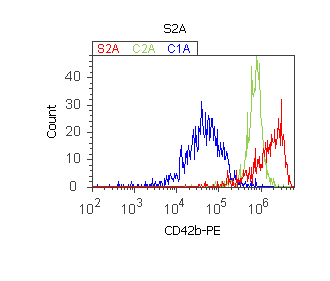

Supplement: Supplementary file 1 [file OncolRes-34-72421-s001.zip › Figure_S6/Figure_S6_S2A1.tiff]

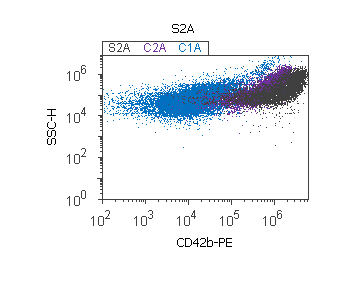

Supplement: Supplementary file 1 [file OncolRes-34-72421-s001.zip › Figure_S6/Figure_S6_S2A2.tiff]

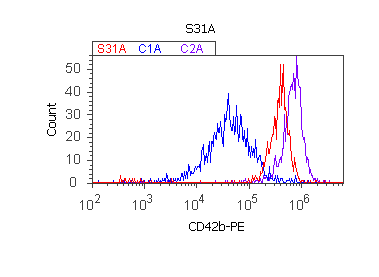

Supplement: Supplementary file 1 [file OncolRes-34-72421-s001.zip › Figure_S6/Figure_S6_S31C1.tiff]

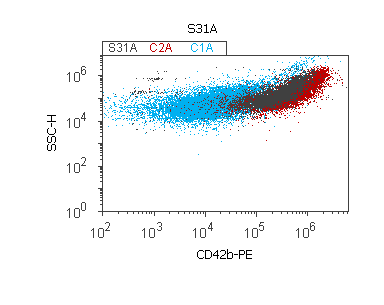

Supplement: Supplementary file 1 [file OncolRes-34-72421-s001.zip › Figure_S6/Figure_S6_S31C2.tiff]

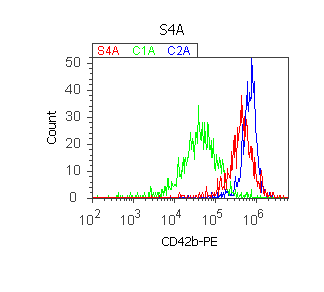

Supplement: Supplementary file 1 [file OncolRes-34-72421-s001.zip › Figure_S6/Figure_S6_S4A1.tiff]

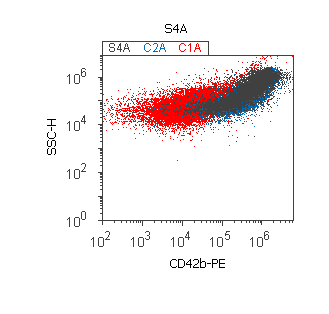

Supplement: Supplementary file 1 [file OncolRes-34-72421-s001.zip › Figure_S6/Figure_S6_S4A2.tiff]

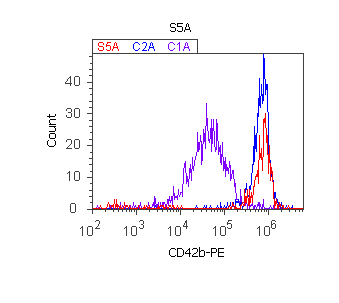

Supplement: Supplementary file 1 [file OncolRes-34-72421-s001.zip › Figure_S6/Figure_S6_S5B1.tiff]

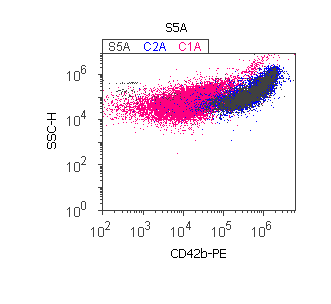

Supplement: Supplementary file 1 [file OncolRes-34-72421-s001.zip › Figure_S6/Figure_S6_S5B2.tiff]

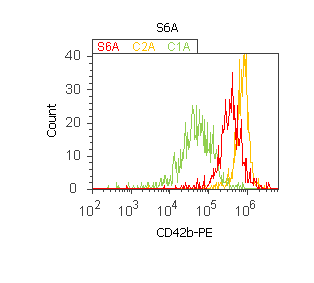

Supplement: Supplementary file 1 [file OncolRes-34-72421-s001.zip › Figure_S6/Figure_S6_S6B1.tiff]

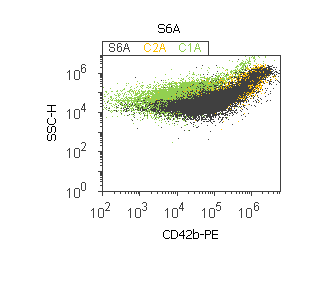

Supplement: Supplementary file 1 [file OncolRes-34-72421-s001.zip › Figure_S6/Figure_S6_S6B2.tiff]

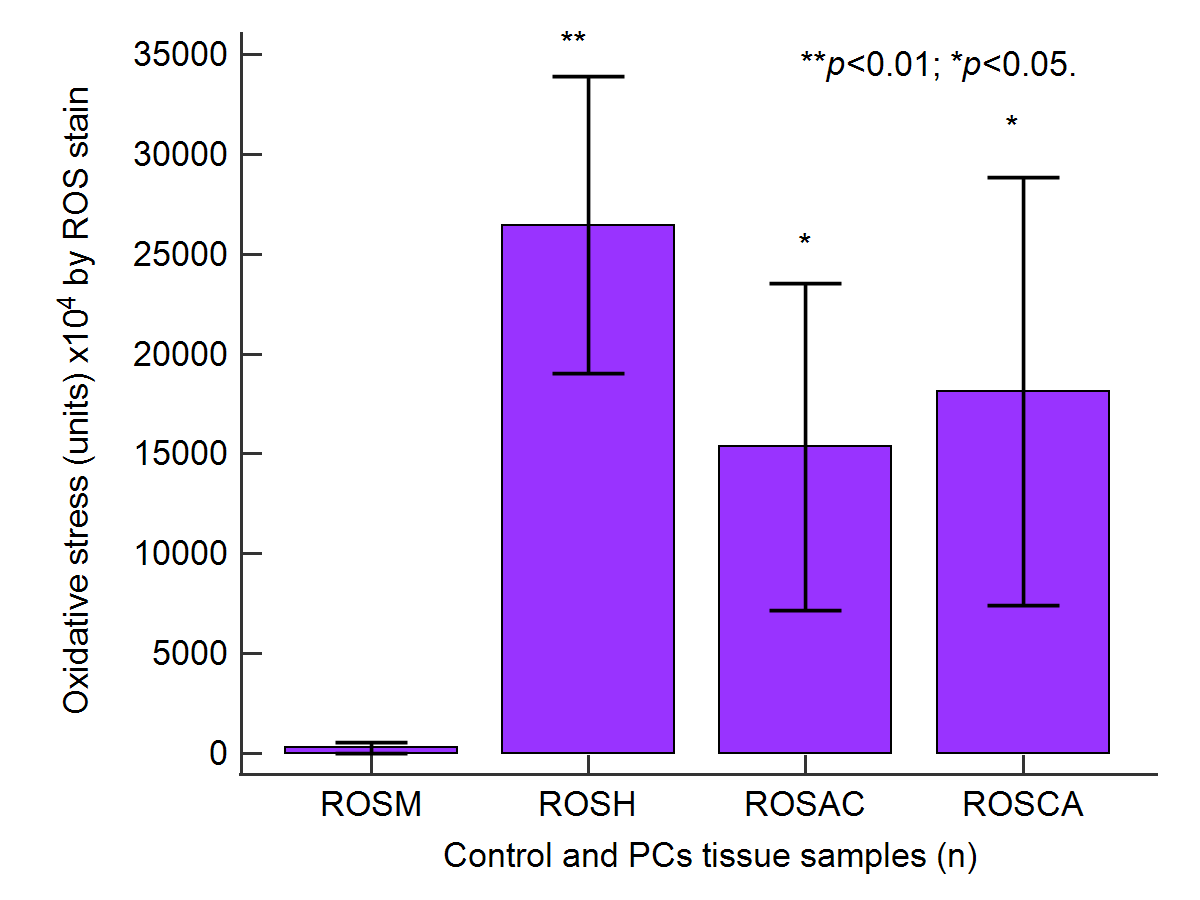

Supplement: Supplementary file 1 [file OncolRes-34-72421-s001.zip › Figure_S7/Figure_S7D.tif]

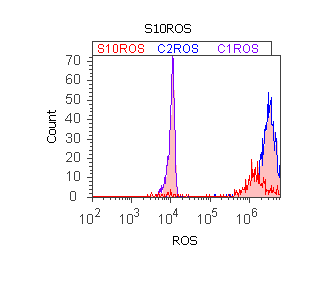

Supplement: Supplementary file 1 [file OncolRes-34-72421-s001.zip › Figure_S7/Figure_S7_S10B1.tiff]

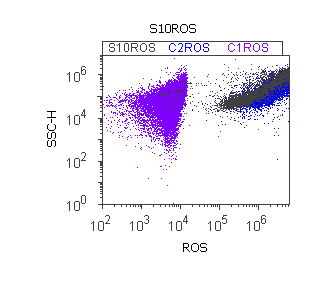

Supplement: Supplementary file 1 [file OncolRes-34-72421-s001.zip › Figure_S7/Figure_S7_S10B2.tiff]

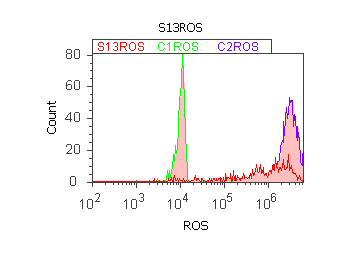

Supplement: Supplementary file 1 [file OncolRes-34-72421-s001.zip › Figure_S7/Figure_S7_S13C1.tiff]

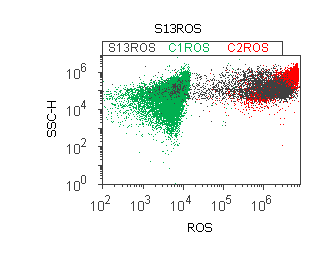

Supplement: Supplementary file 1 [file OncolRes-34-72421-s001.zip › Figure_S7/Figure_S7_S13C2.tiff]

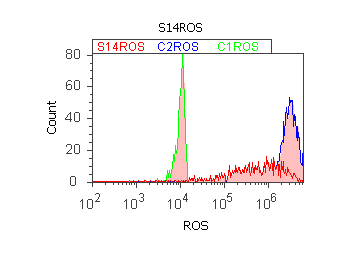

Supplement: Supplementary file 1 [file OncolRes-34-72421-s001.zip › Figure_S7/Figure_S7_S14C1.tiff]

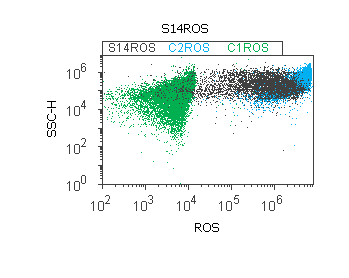

Supplement: Supplementary file 1 [file OncolRes-34-72421-s001.zip › Figure_S7/Figure_S7_S14C2.tiff]

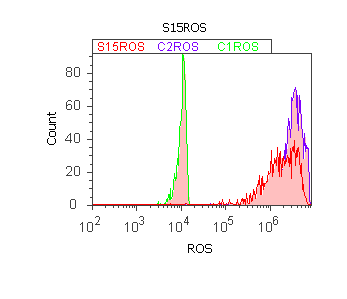

Supplement: Supplementary file 1 [file OncolRes-34-72421-s001.zip › Figure_S7/Figure_S7_S15A1.tiff]

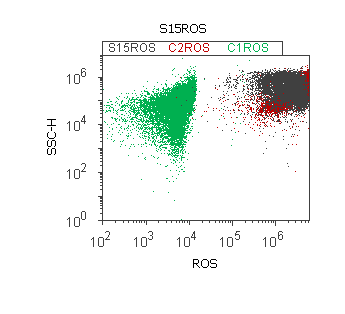

Supplement: Supplementary file 1 [file OncolRes-34-72421-s001.zip › Figure_S7/Figure_S7_S15A2.tiff]

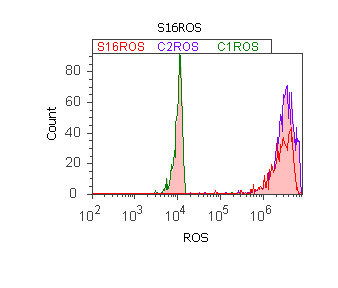

Supplement: Supplementary file 1 [file OncolRes-34-72421-s001.zip › Figure_S7/Figure_S7_S16C1.tiff]

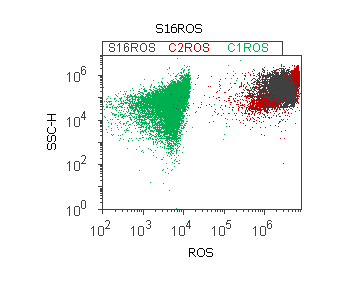

Supplement: Supplementary file 1 [file OncolRes-34-72421-s001.zip › Figure_S7/Figure_S7_S16C2.tiff]

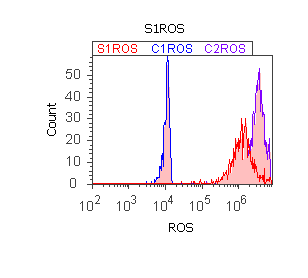

Supplement: Supplementary file 1 [file OncolRes-34-72421-s001.zip › Figure_S7/Figure_S7_S1A1.tiff]

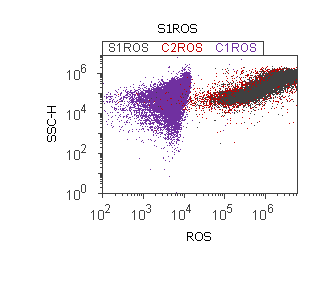

Supplement: Supplementary file 1 [file OncolRes-34-72421-s001.zip › Figure_S7/Figure_S7_S1A2.tiff]

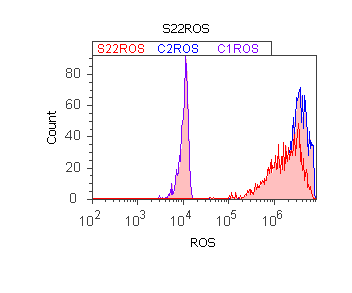

Supplement: Supplementary file 1 [file OncolRes-34-72421-s001.zip › Figure_S7/Figure_S7_S22B1.tiff]

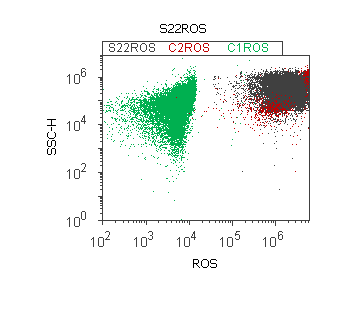

Supplement: Supplementary file 1 [file OncolRes-34-72421-s001.zip › Figure_S7/Figure_S7_S22B2.tiff]

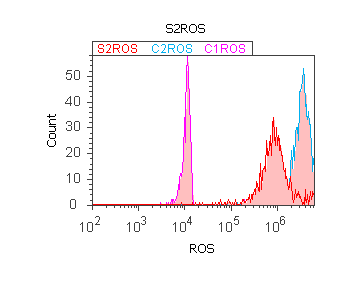

Supplement: Supplementary file 1 [file OncolRes-34-72421-s001.zip › Figure_S7/Figure_S7_S2A1.tiff]

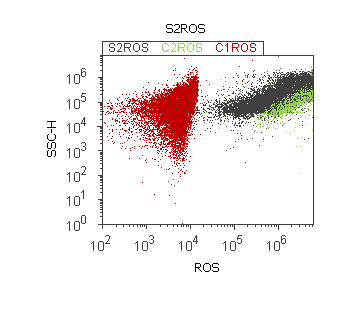

Supplement: Supplementary file 1 [file OncolRes-34-72421-s001.zip › Figure_S7/Figure_S7_S2A2.tiff]

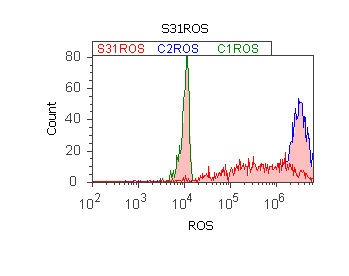

Supplement: Supplementary file 1 [file OncolRes-34-72421-s001.zip › Figure_S7/Figure_S7_S31C1.tiff]

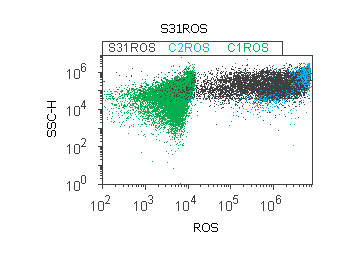

Supplement: Supplementary file 1 [file OncolRes-34-72421-s001.zip › Figure_S7/Figure_S7_S31C2.tiff]

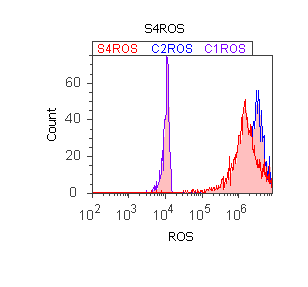

Supplement: Supplementary file 1 [file OncolRes-34-72421-s001.zip › Figure_S7/Figure_S7_S4A1.tiff]

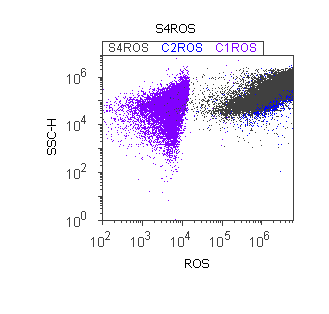

Supplement: Supplementary file 1 [file OncolRes-34-72421-s001.zip › Figure_S7/Figure_S7_S4A2.tiff]

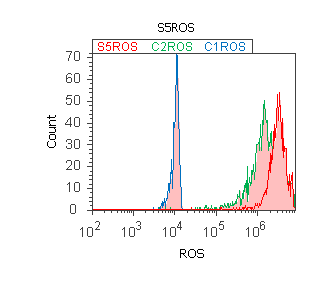

Supplement: Supplementary file 1 [file OncolRes-34-72421-s001.zip › Figure_S7/Figure_S7_S5B1.tiff]

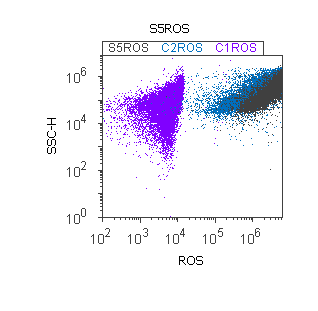

Supplement: Supplementary file 1 [file OncolRes-34-72421-s001.zip › Figure_S7/Figure_S7_S5B2.tiff]

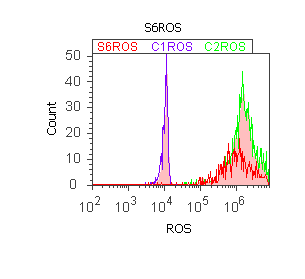

Supplement: Supplementary file 1 [file OncolRes-34-72421-s001.zip › Figure_S7/Figure_S7_S6B1.tiff]

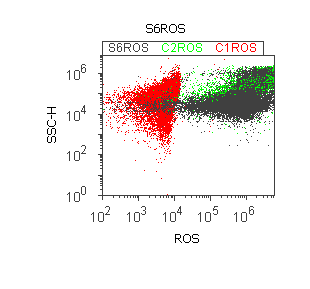

Supplement: Supplementary file 1 [file OncolRes-34-72421-s001.zip › Figure_S7/Figure_S7_S6B2.tiff]

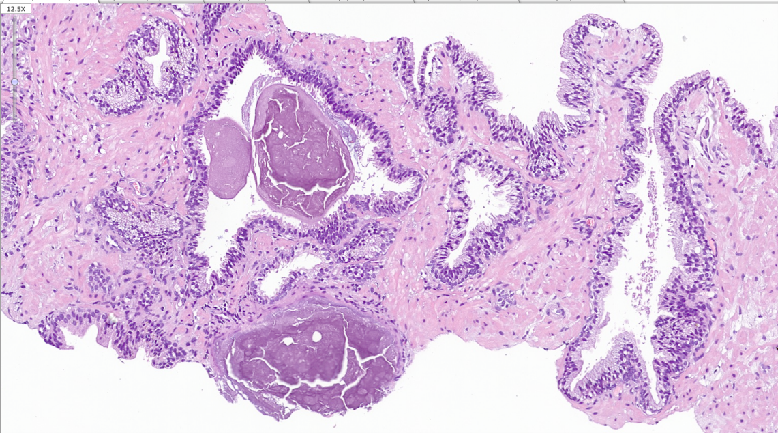

Supplement: Supplementary file 1 [file OncolRes-34-72421-s001.zip › Figure_S8/Figure_S8A.tif]

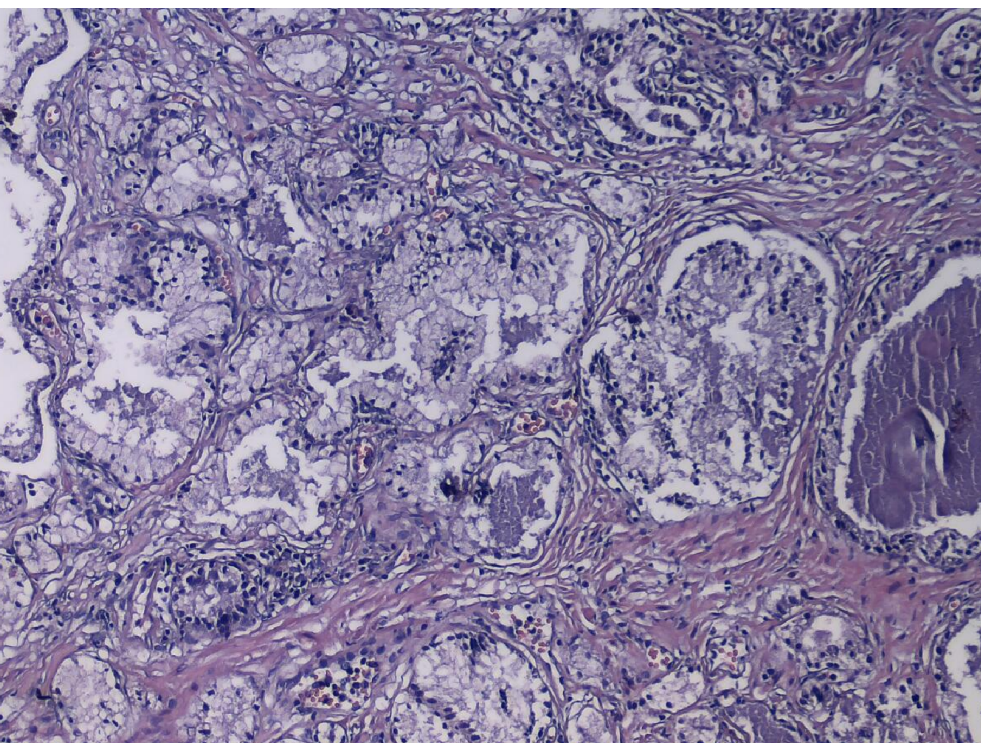

Supplement: Supplementary file 1 [file OncolRes-34-72421-s001.zip › Figure_S8/Figure_S8B.tif]

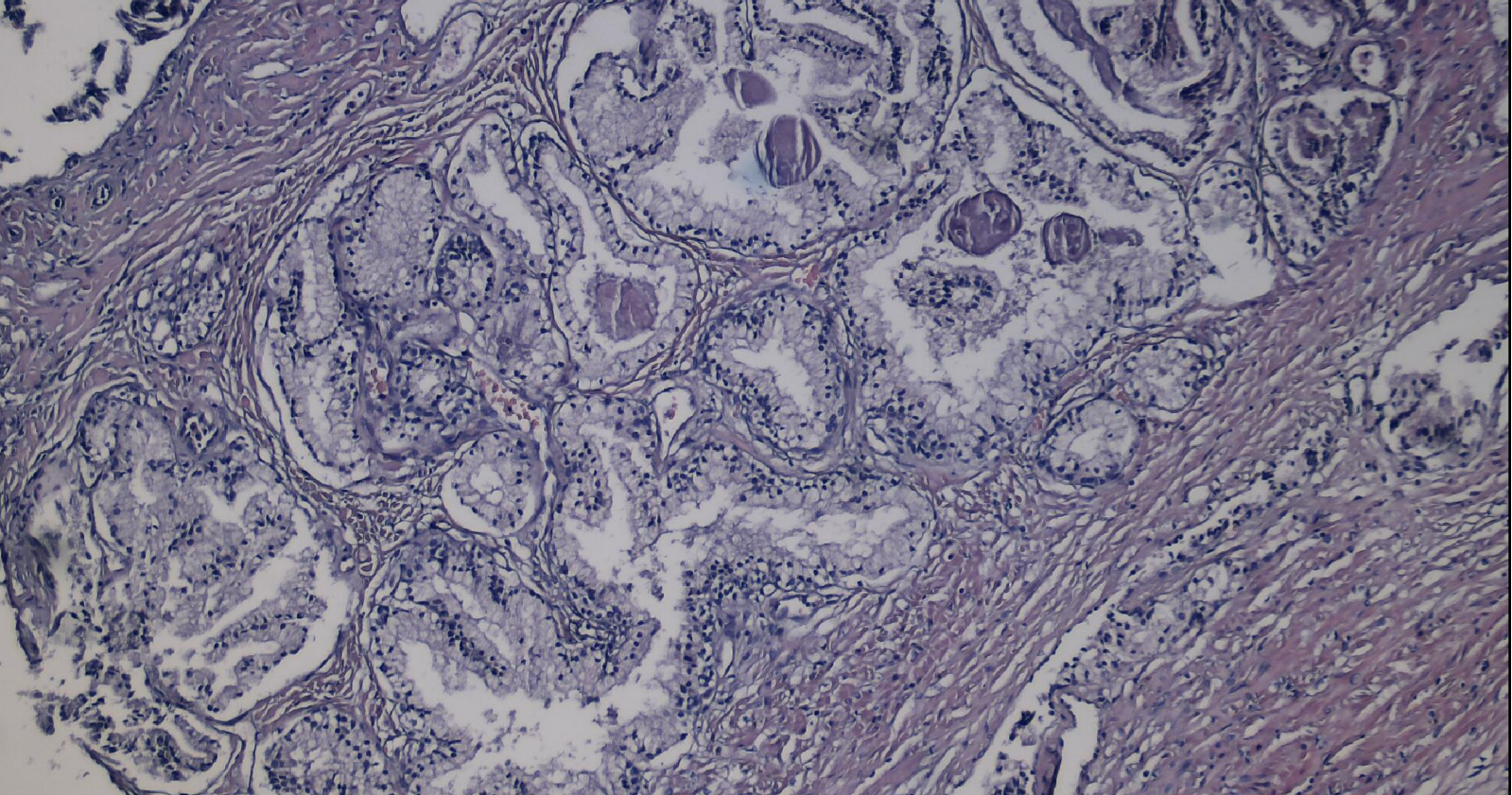

Supplement: Supplementary file 1 [file OncolRes-34-72421-s001.zip › Figure_S8/Figure_S8C.tif]

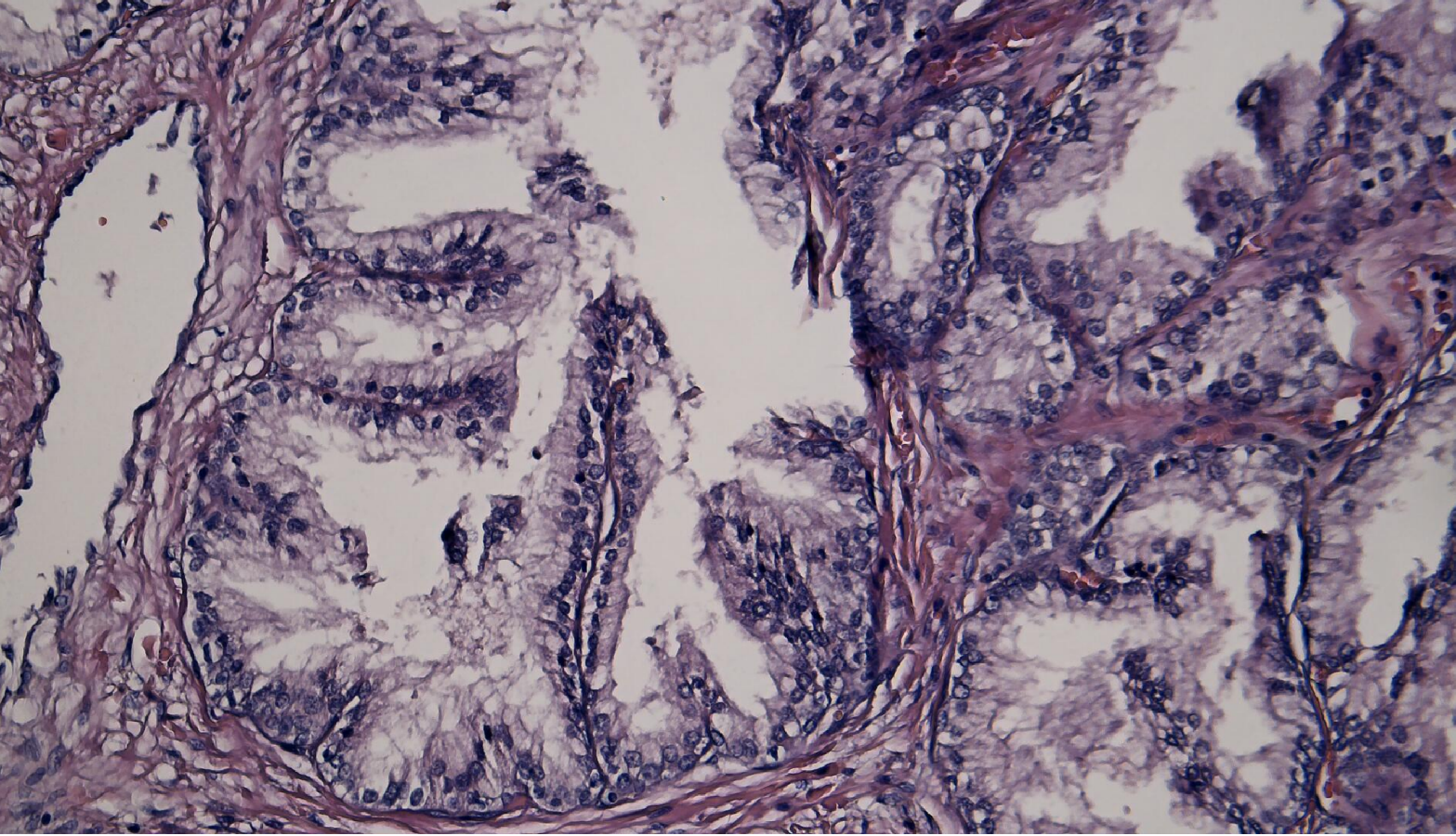

Supplement: Supplementary file 1 [file OncolRes-34-72421-s001.zip › Figure_S8/Figure_S8D.tif]

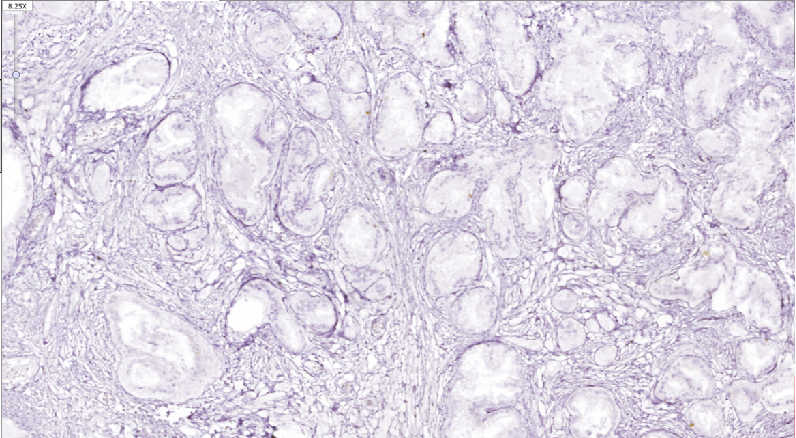

Supplement: Supplementary file 1 [file OncolRes-34-72421-s001.zip › Figure_S8/Figure_S8E.tif]

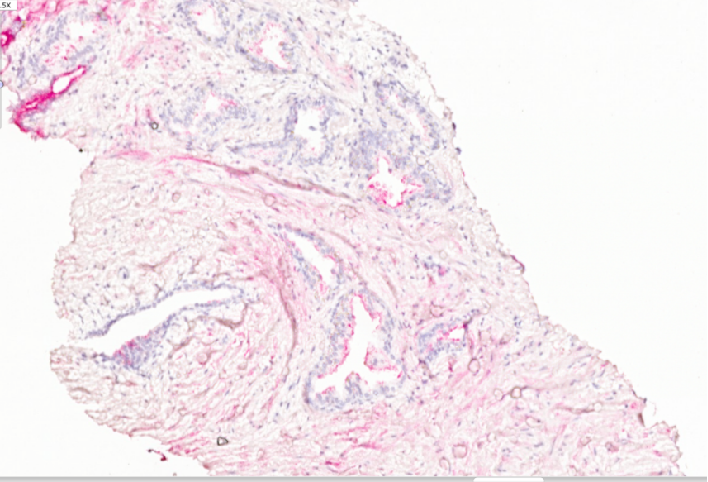

Supplement: Supplementary file 1 [file OncolRes-34-72421-s001.zip › Figure_S8/Figure_S8F.tif]

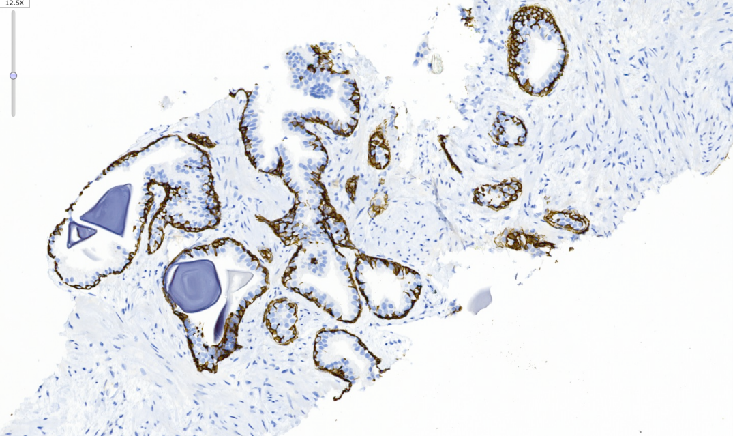

Supplement: Supplementary file 1 [file OncolRes-34-72421-s001.zip › Figure_S8/Figure_S8G.tif]

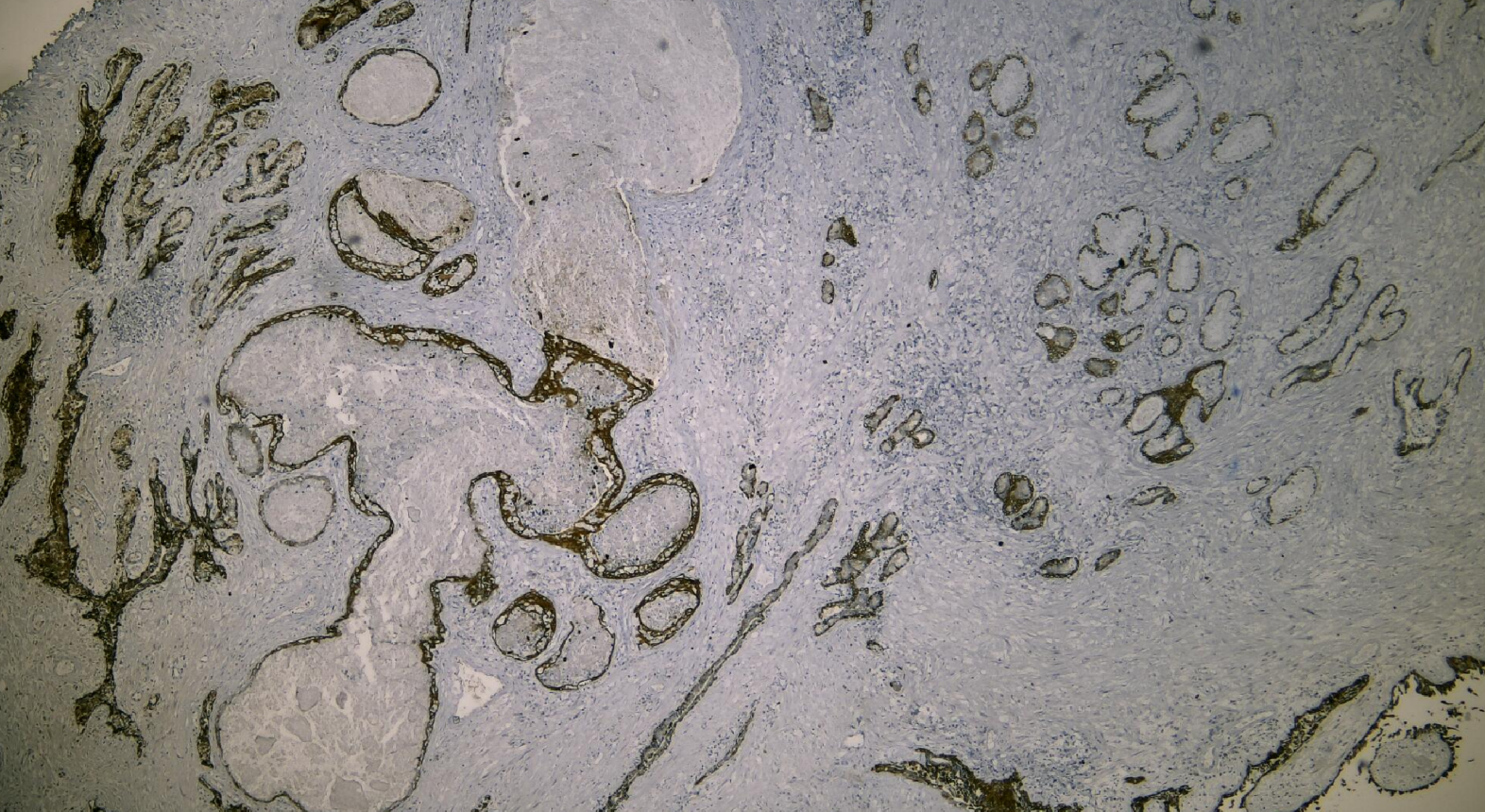

Supplement: Supplementary file 1 [file OncolRes-34-72421-s001.zip › Figure_S8/Figure_S8H.tif]

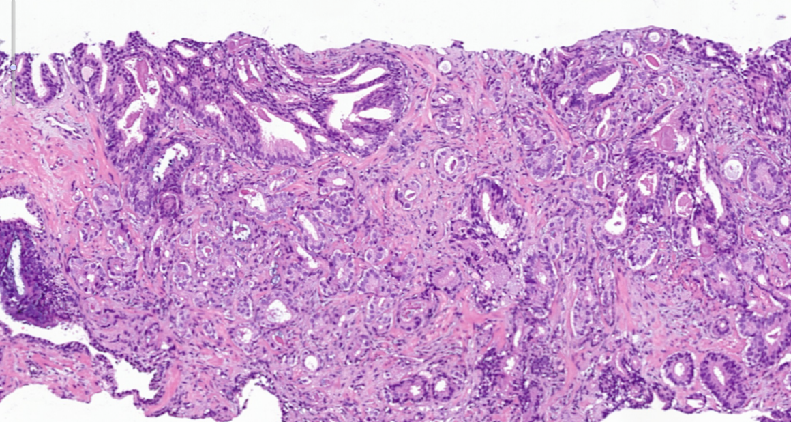

Supplement: Supplementary file 1 [file OncolRes-34-72421-s001.zip › Figure_S8/Figure_S8I.tif]

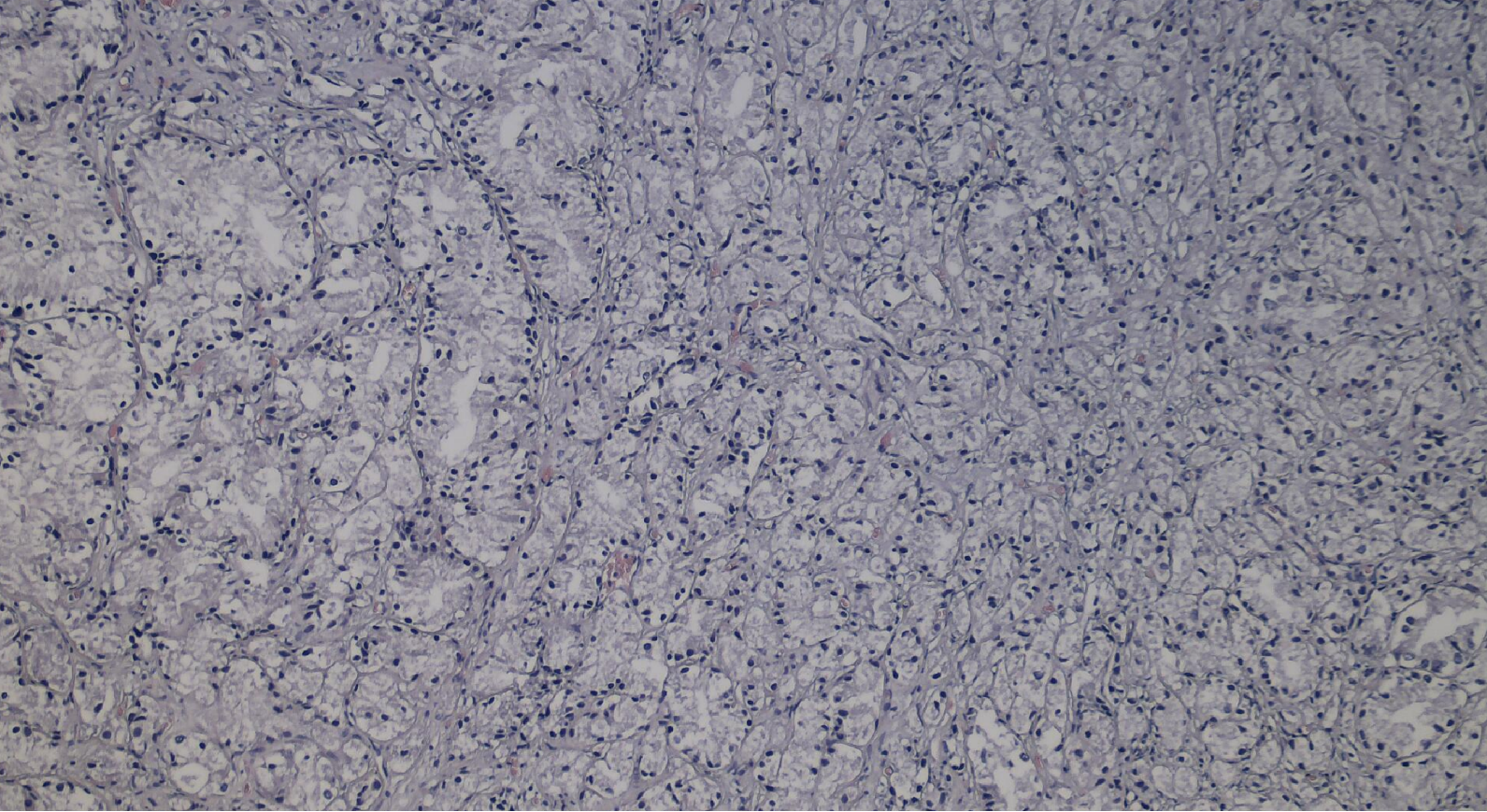

Supplement: Supplementary file 1 [file OncolRes-34-72421-s001.zip › Figure_S8/Figure_S8J.tif]

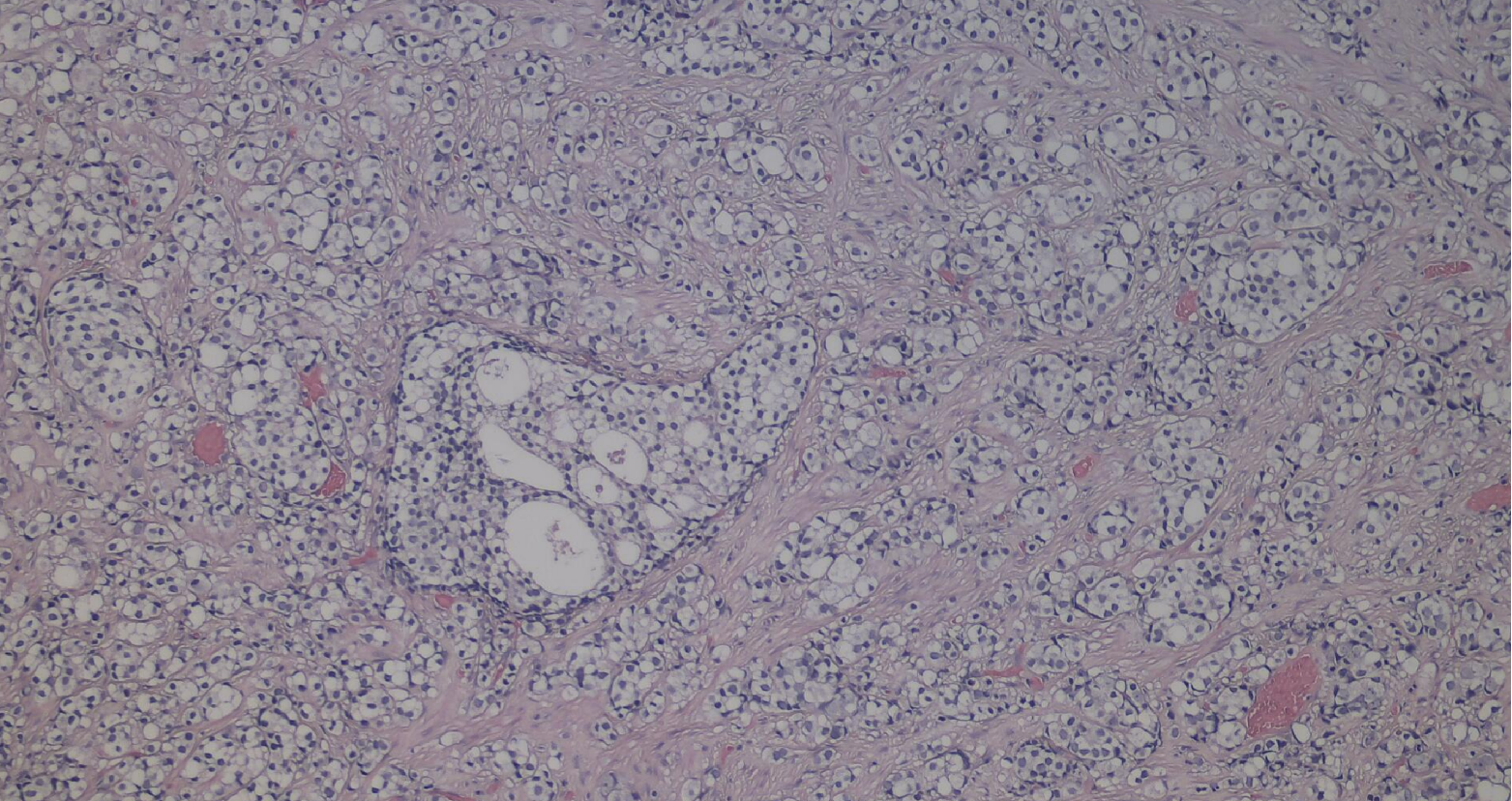

Supplement: Supplementary file 1 [file OncolRes-34-72421-s001.zip › Figure_S8/Figure_S8K.tif]

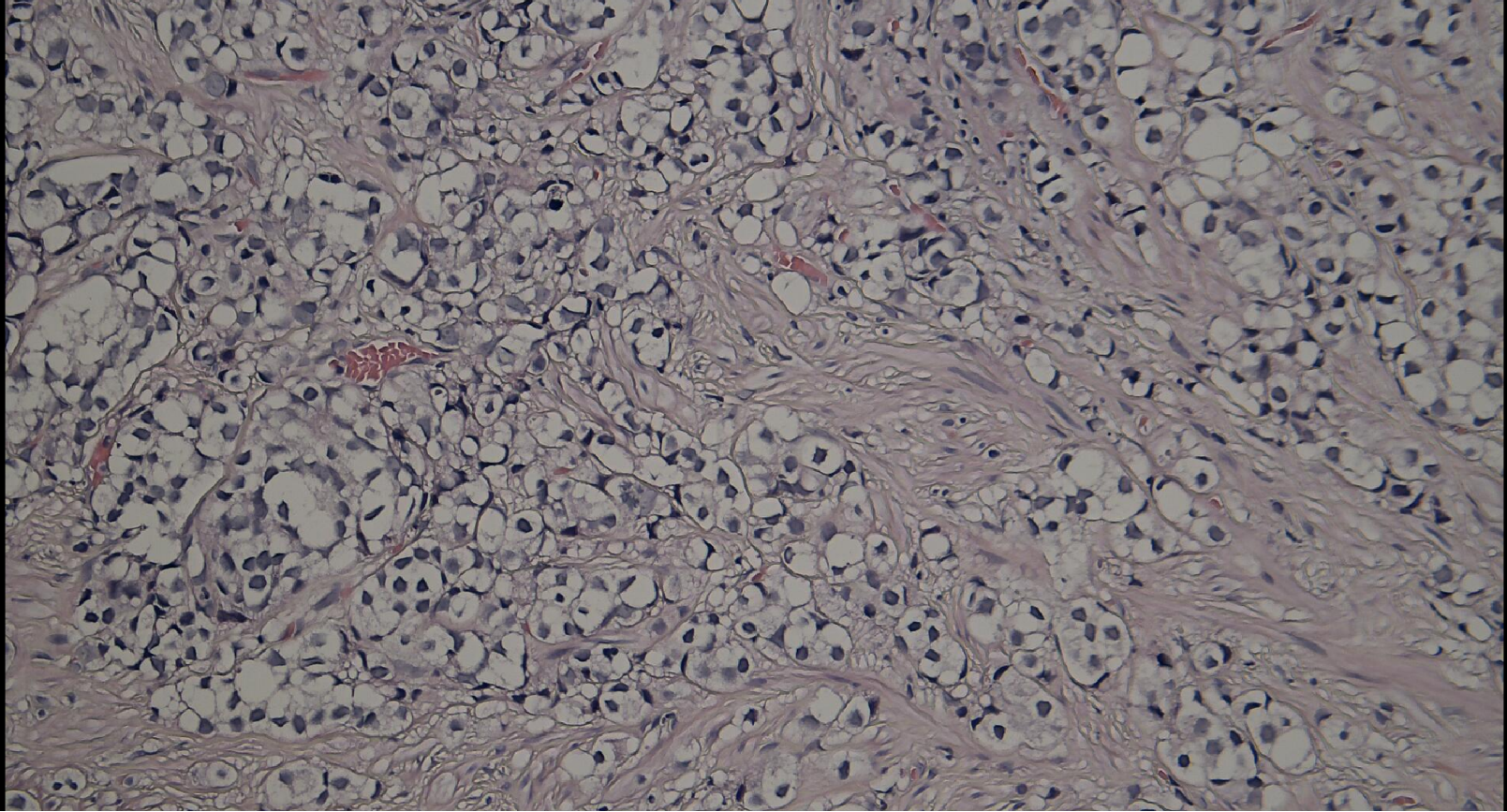

Supplement: Supplementary file 1 [file OncolRes-34-72421-s001.zip › Figure_S8/Figure_S8L.tif]

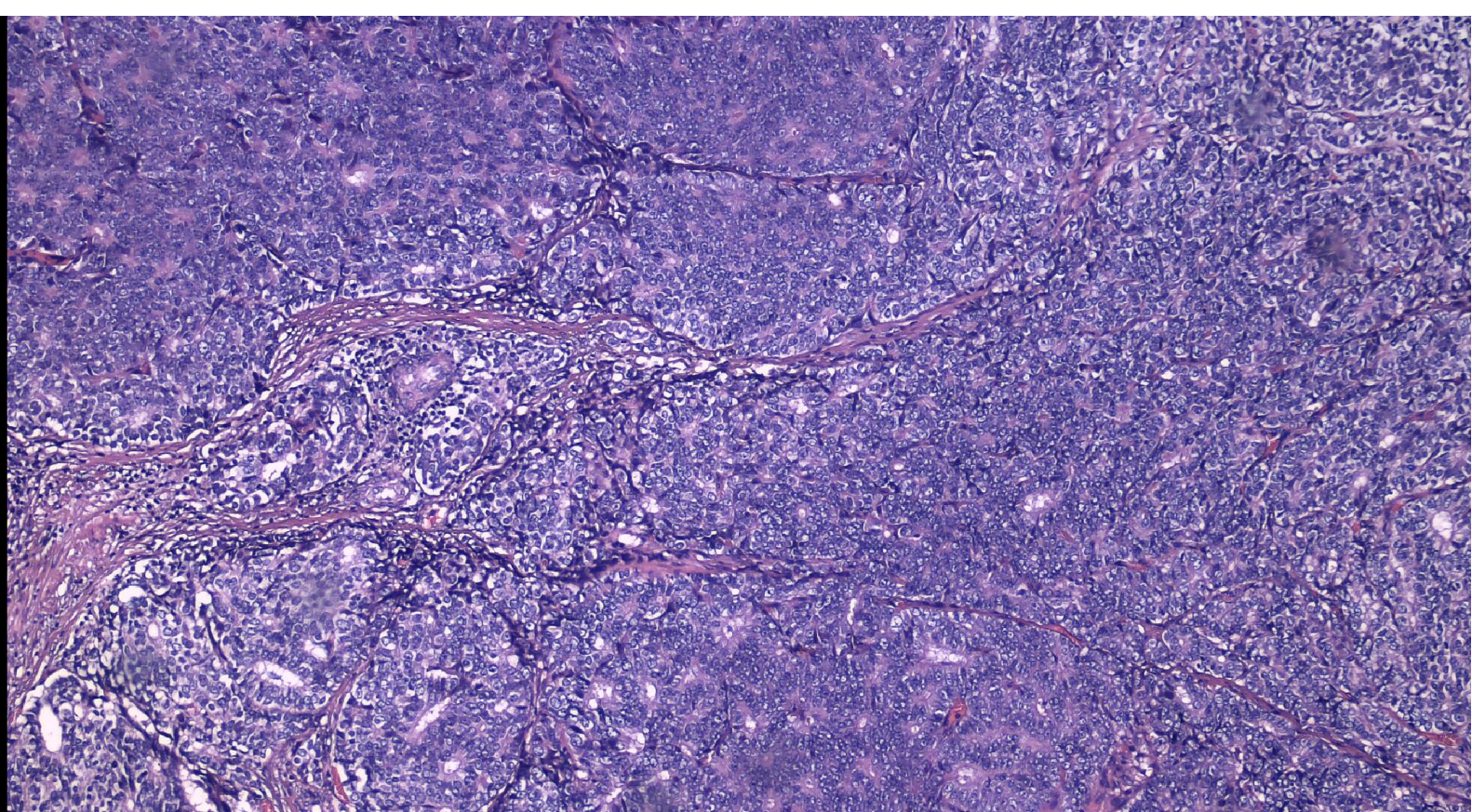

Supplement: Supplementary file 1 [file OncolRes-34-72421-s001.zip › Figure_S8/Figure_S8M.tif]

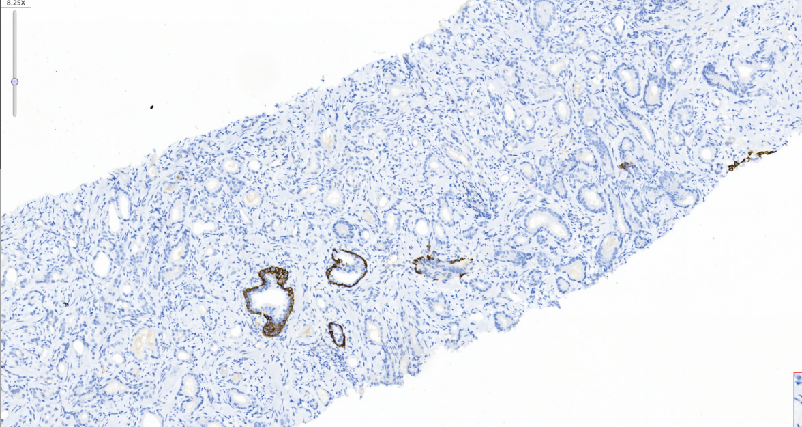

Supplement: Supplementary file 1 [file OncolRes-34-72421-s001.zip › Figure_S8/Figure_S8N.tif]

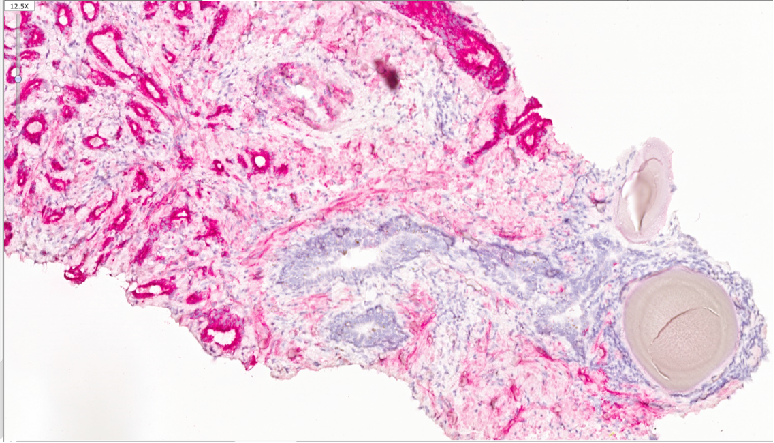

Supplement: Supplementary file 1 [file OncolRes-34-72421-s001.zip › Figure_S8/Figure_S8O.tif]

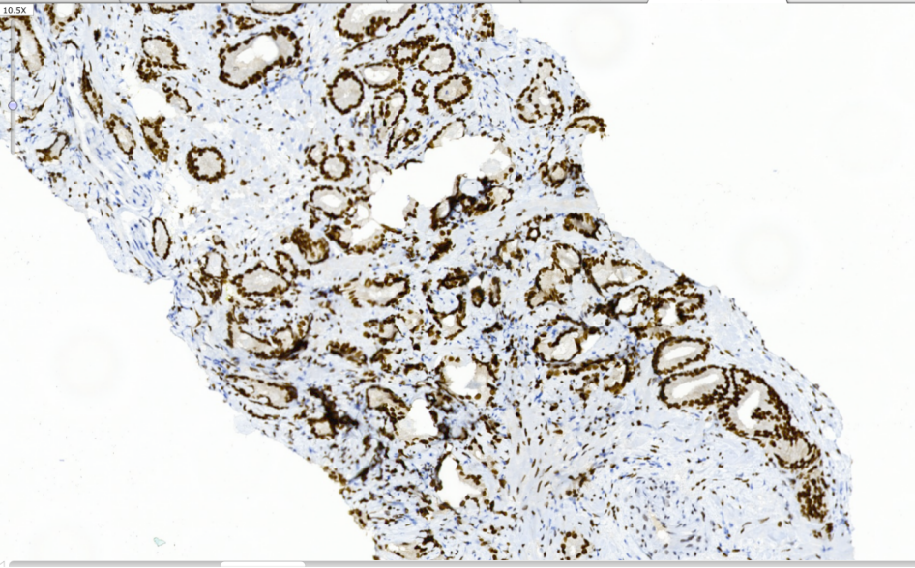

Supplement: Supplementary file 1 [file OncolRes-34-72421-s001.zip › Figure_S8/Figure_S8P.tif]

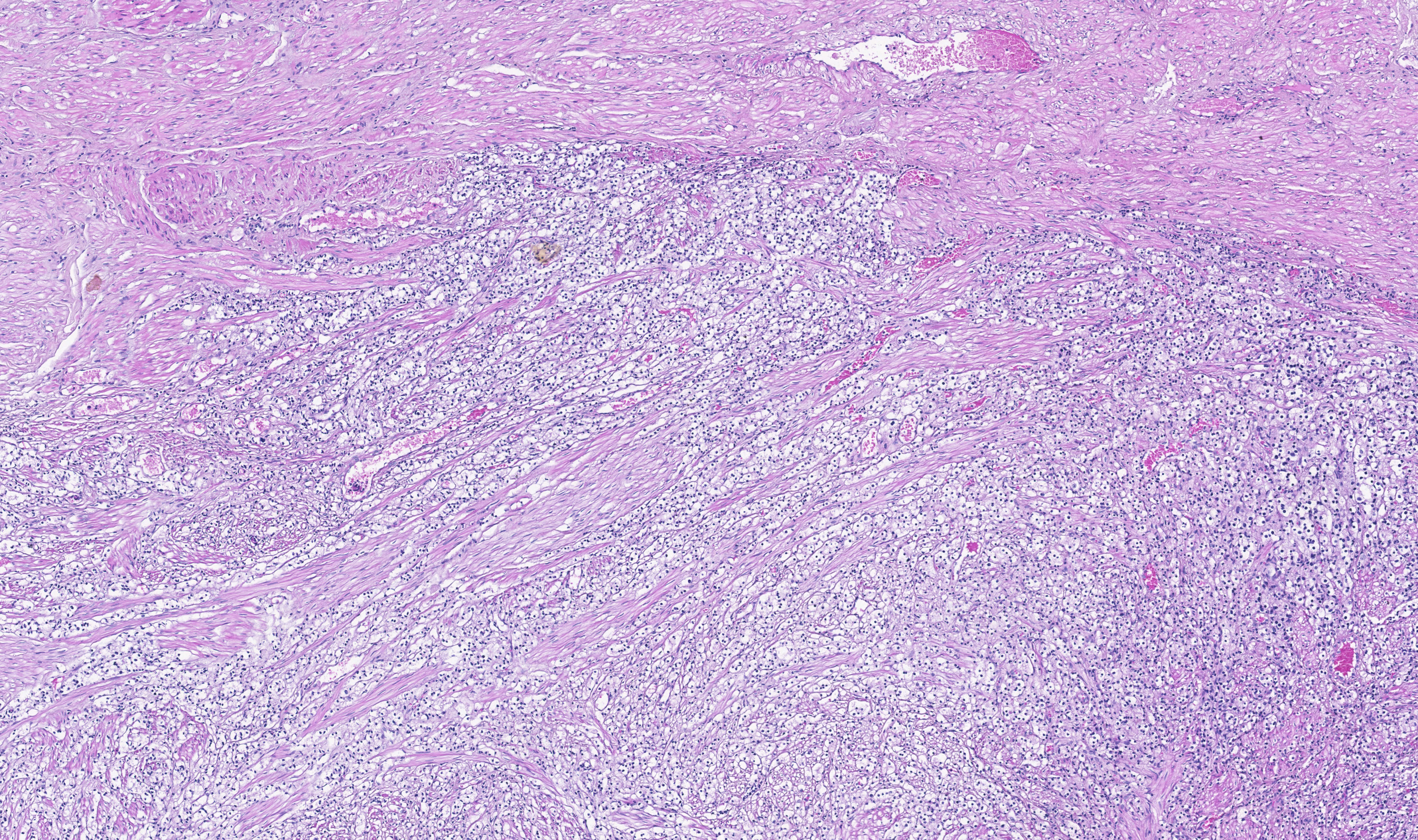

Supplement: Supplementary file 1 [file OncolRes-34-72421-s001.zip › Figure_S8/Figure_S8Q.tif]

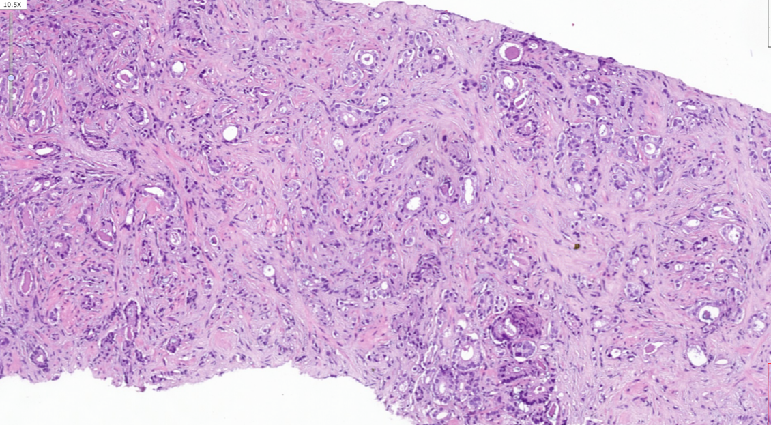

Supplement: Supplementary file 1 [file OncolRes-34-72421-s001.zip › Figure_S8/Figure_S8R.tif]

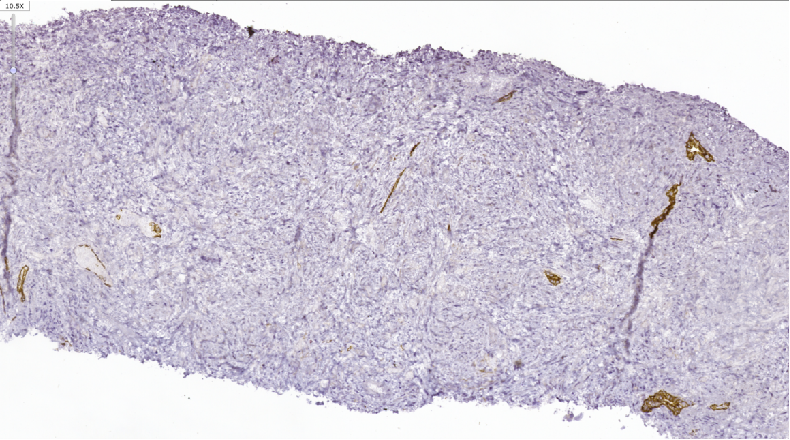

Supplement: Supplementary file 1 [file OncolRes-34-72421-s001.zip › Figure_S8/Figure_S8S.tif]

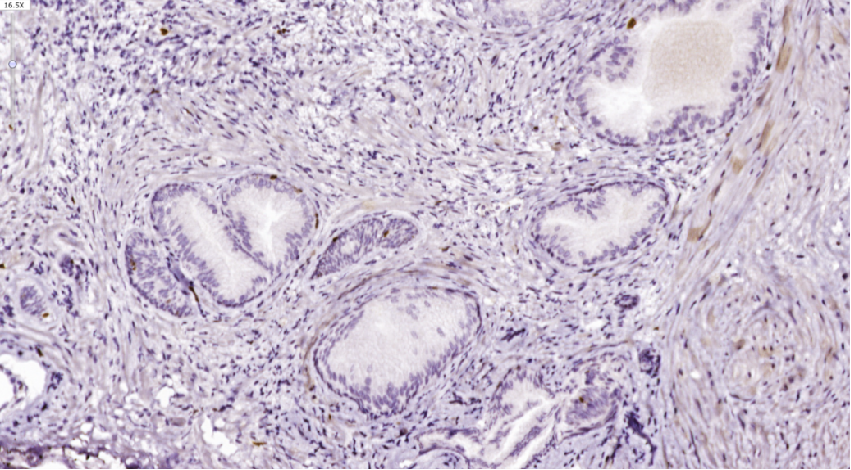

Supplement: Supplementary file 1 [file OncolRes-34-72421-s001.zip › Figure_S8/Figure_S8T.tif]
