# Supplementary figures and images for: Dysregulated Cell Signaling Pathways in Prostate Tumoral Plasticity—Checkpoints (part 1 of 2)
Source: Oncol Res. 2026 May 21;34(6):16. doi: 10.32604/or.2026.072421 (PMC13227601; doi:10.32604/or.2026.072421)

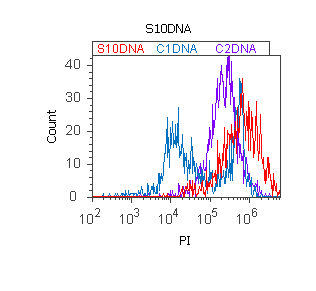

Supplement: Supplementary file 1 [file OncolRes-34-72421-s001.zip › Figure_S1/Figure_S1_S10B1.tiff]

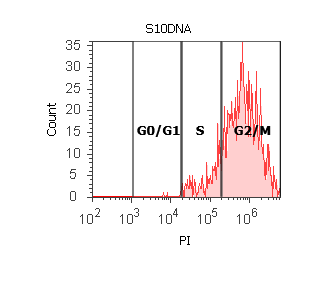

Supplement: Supplementary file 1 [file OncolRes-34-72421-s001.zip › Figure_S1/Figure_S1_S10B2.tiff]

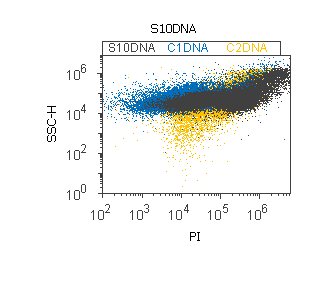

Supplement: Supplementary file 1 [file OncolRes-34-72421-s001.zip › Figure_S1/Figure_S1_S10B3.tiff]

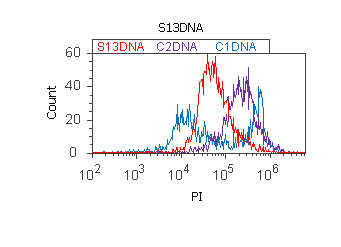

Supplement: Supplementary file 1 [file OncolRes-34-72421-s001.zip › Figure_S1/Figure_S1_S13C1.tiff]

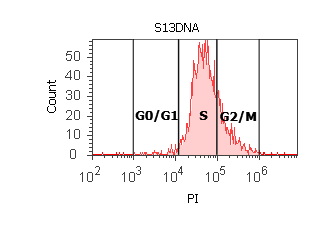

Supplement: Supplementary file 1 [file OncolRes-34-72421-s001.zip › Figure_S1/Figure_S1_S13C2.tiff]

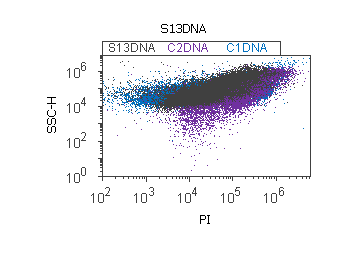

Supplement: Supplementary file 1 [file OncolRes-34-72421-s001.zip › Figure_S1/Figure_S1_S13C3.tiff]

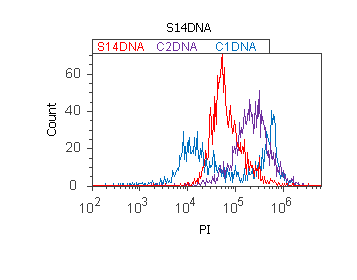

Supplement: Supplementary file 1 [file OncolRes-34-72421-s001.zip › Figure_S1/Figure_S1_S14C1.tiff]

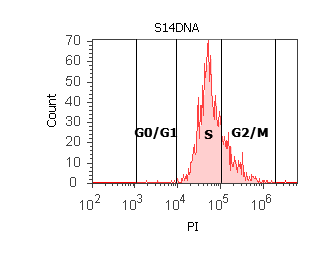

Supplement: Supplementary file 1 [file OncolRes-34-72421-s001.zip › Figure_S1/Figure_S1_S14C2.tiff]

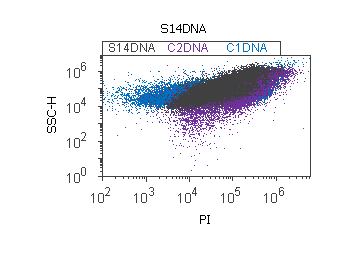

Supplement: Supplementary file 1 [file OncolRes-34-72421-s001.zip › Figure_S1/Figure_S1_S14C3.tiff]

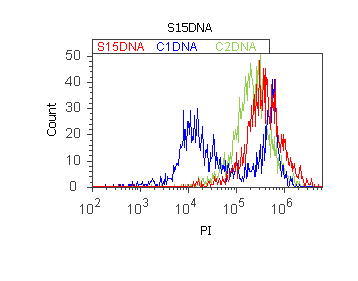

Supplement: Supplementary file 1 [file OncolRes-34-72421-s001.zip › Figure_S1/Figure_S1_S15A1.tiff]

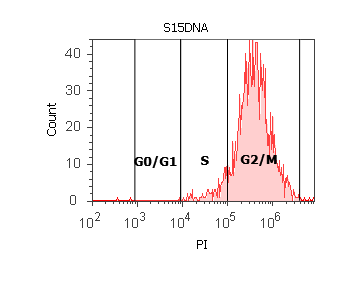

Supplement: Supplementary file 1 [file OncolRes-34-72421-s001.zip › Figure_S1/Figure_S1_S15A2.tiff]

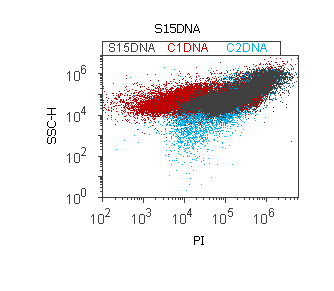

Supplement: Supplementary file 1 [file OncolRes-34-72421-s001.zip › Figure_S1/Figure_S1_S15A3.tiff]

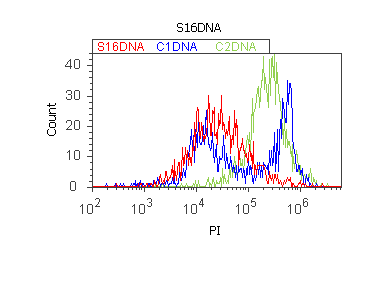

Supplement: Supplementary file 1 [file OncolRes-34-72421-s001.zip › Figure_S1/Figure_S1_S16C1.tiff]

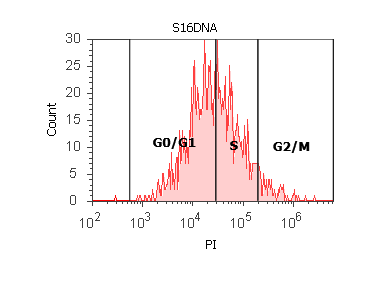

Supplement: Supplementary file 1 [file OncolRes-34-72421-s001.zip › Figure_S1/Figure_S1_S16C2.tiff]

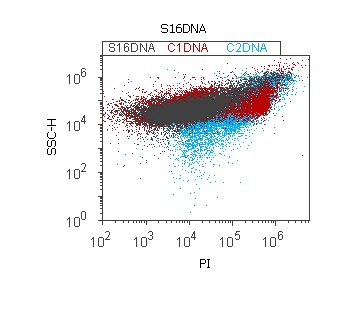

Supplement: Supplementary file 1 [file OncolRes-34-72421-s001.zip › Figure_S1/Figure_S1_S16C3.tiff]

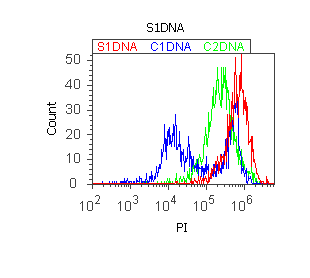

Supplement: Supplementary file 1 [file OncolRes-34-72421-s001.zip › Figure_S1/Figure_S1_S1A1.tiff]

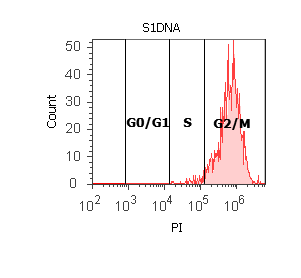

Supplement: Supplementary file 1 [file OncolRes-34-72421-s001.zip › Figure_S1/Figure_S1_S1A2.tiff]

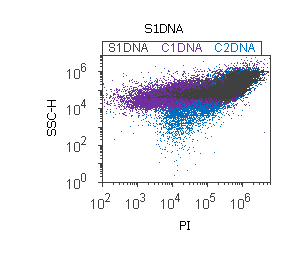

Supplement: Supplementary file 1 [file OncolRes-34-72421-s001.zip › Figure_S1/Figure_S1_S1A3.tiff]

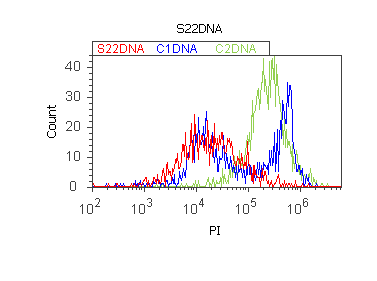

Supplement: Supplementary file 1 [file OncolRes-34-72421-s001.zip › Figure_S1/Figure_S1_S22B1.tiff]

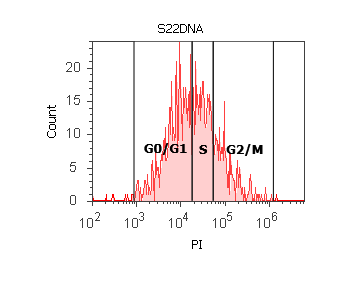

Supplement: Supplementary file 1 [file OncolRes-34-72421-s001.zip › Figure_S1/Figure_S1_S22B2.tiff]

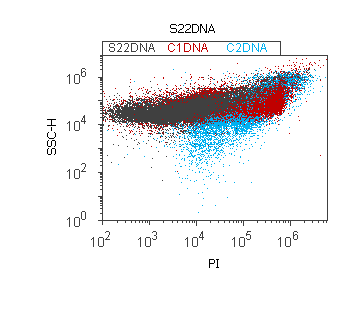

Supplement: Supplementary file 1 [file OncolRes-34-72421-s001.zip › Figure_S1/Figure_S1_S22B3.tiff]

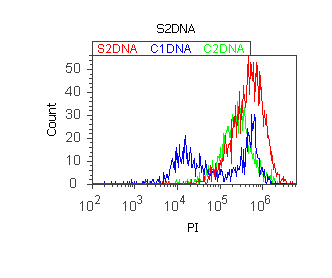

Supplement: Supplementary file 1 [file OncolRes-34-72421-s001.zip › Figure_S1/Figure_S1_S2A1.tiff]

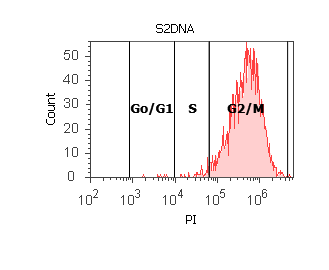

Supplement: Supplementary file 1 [file OncolRes-34-72421-s001.zip › Figure_S1/Figure_S1_S2A2.tiff]

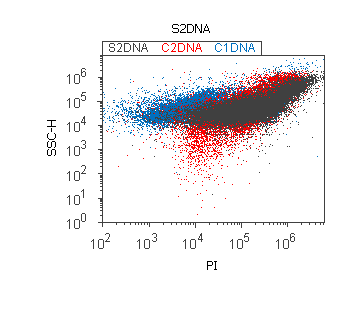

Supplement: Supplementary file 1 [file OncolRes-34-72421-s001.zip › Figure_S1/Figure_S1_S2A3.tiff]

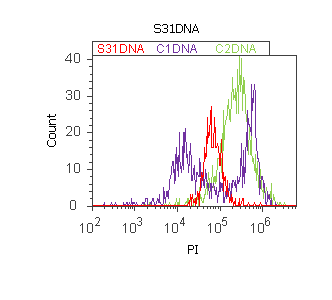

Supplement: Supplementary file 1 [file OncolRes-34-72421-s001.zip › Figure_S1/Figure_S1_S31C1.tiff]

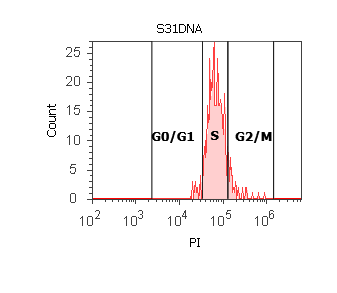

Supplement: Supplementary file 1 [file OncolRes-34-72421-s001.zip › Figure_S1/Figure_S1_S31C2.tiff]

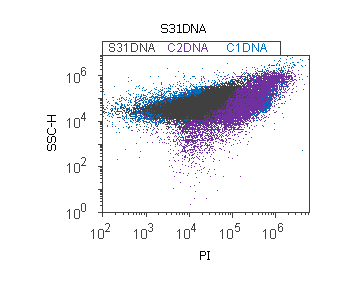

Supplement: Supplementary file 1 [file OncolRes-34-72421-s001.zip › Figure_S1/Figure_S1_S31C3.tiff]

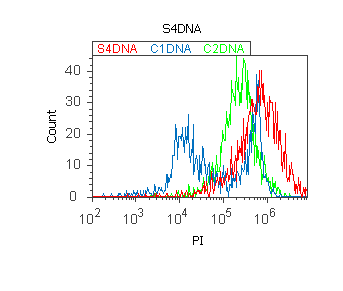

Supplement: Supplementary file 1 [file OncolRes-34-72421-s001.zip › Figure_S1/Figure_S1_S4A1.tiff]

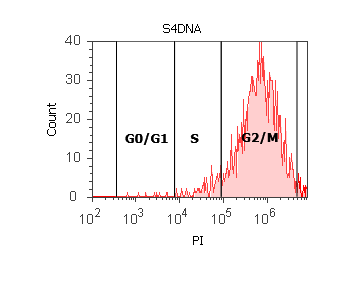

Supplement: Supplementary file 1 [file OncolRes-34-72421-s001.zip › Figure_S1/Figure_S1_S4A2.tiff]

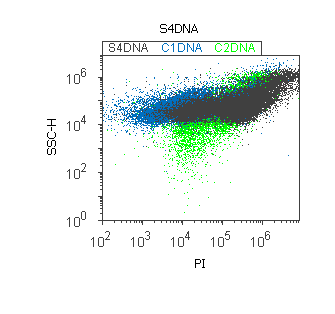

Supplement: Supplementary file 1 [file OncolRes-34-72421-s001.zip › Figure_S1/Figure_S1_S4A3.tiff]

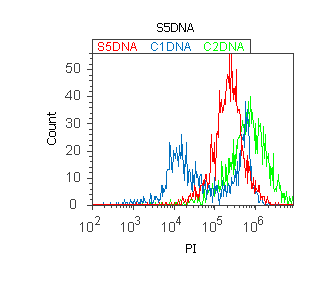

Supplement: Supplementary file 1 [file OncolRes-34-72421-s001.zip › Figure_S1/Figure_S1_S5B1.tiff]

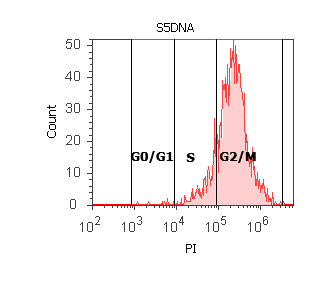

Supplement: Supplementary file 1 [file OncolRes-34-72421-s001.zip › Figure_S1/Figure_S1_S5B2.tiff]

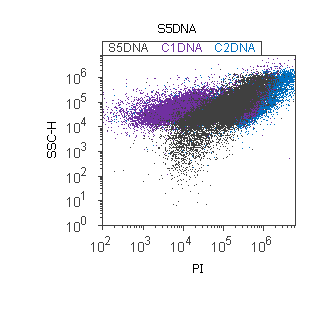

Supplement: Supplementary file 1 [file OncolRes-34-72421-s001.zip › Figure_S1/Figure_S1_S5B3.tiff]

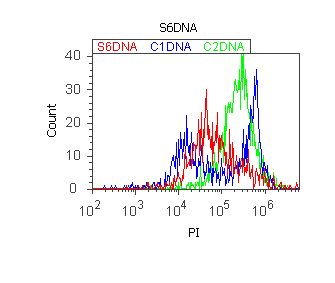

Supplement: Supplementary file 1 [file OncolRes-34-72421-s001.zip › Figure_S1/Figure_S1_S6B1.tiff]

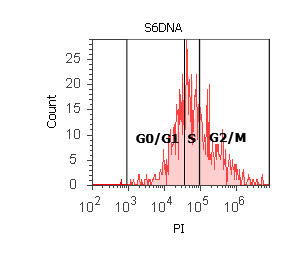

Supplement: Supplementary file 1 [file OncolRes-34-72421-s001.zip › Figure_S1/Figure_S1_S6B2.tiff]

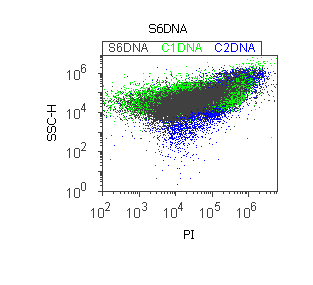

Supplement: Supplementary file 1 [file OncolRes-34-72421-s001.zip › Figure_S1/Figure_S1_S6B3.tiff]

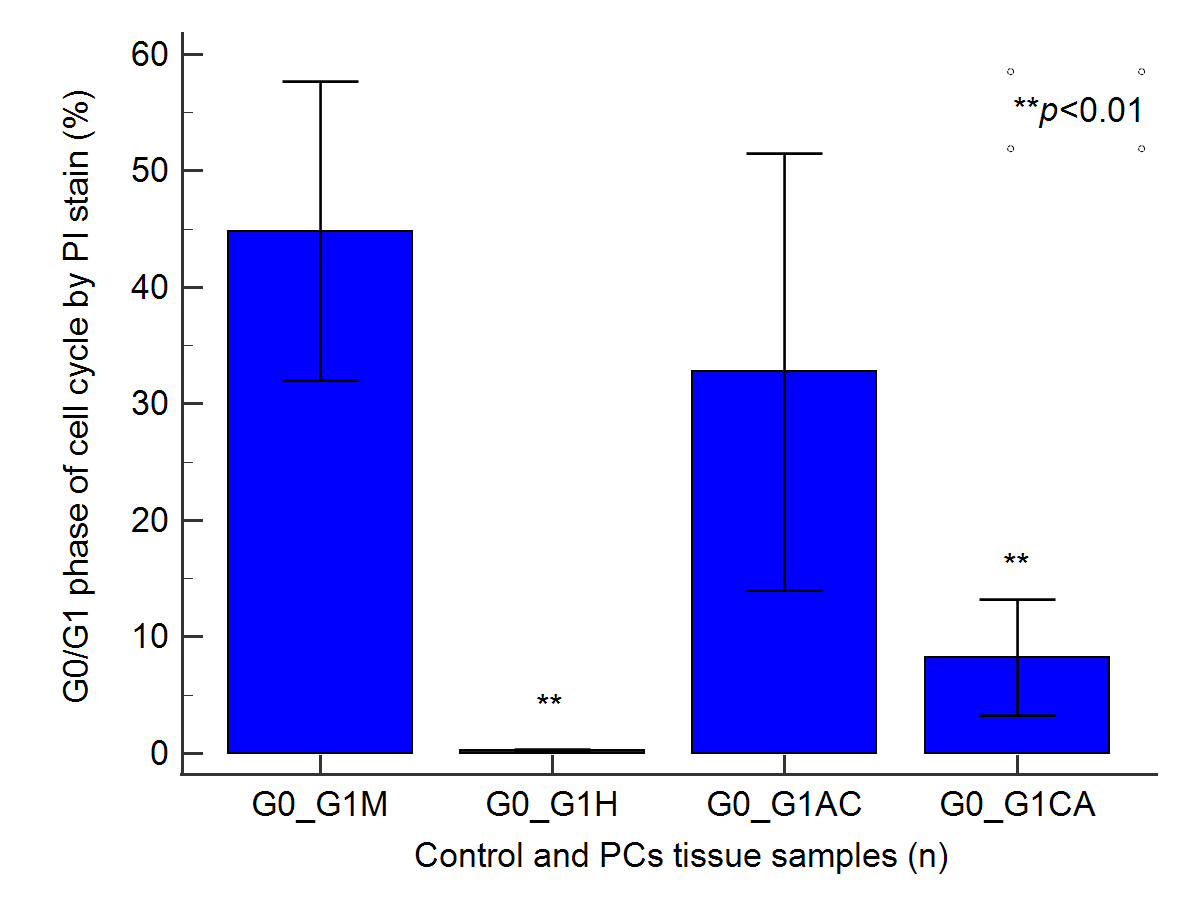

Supplement: Supplementary file 1 [file OncolRes-34-72421-s001.zip › Figure_S2/Figure_S2A.tif]

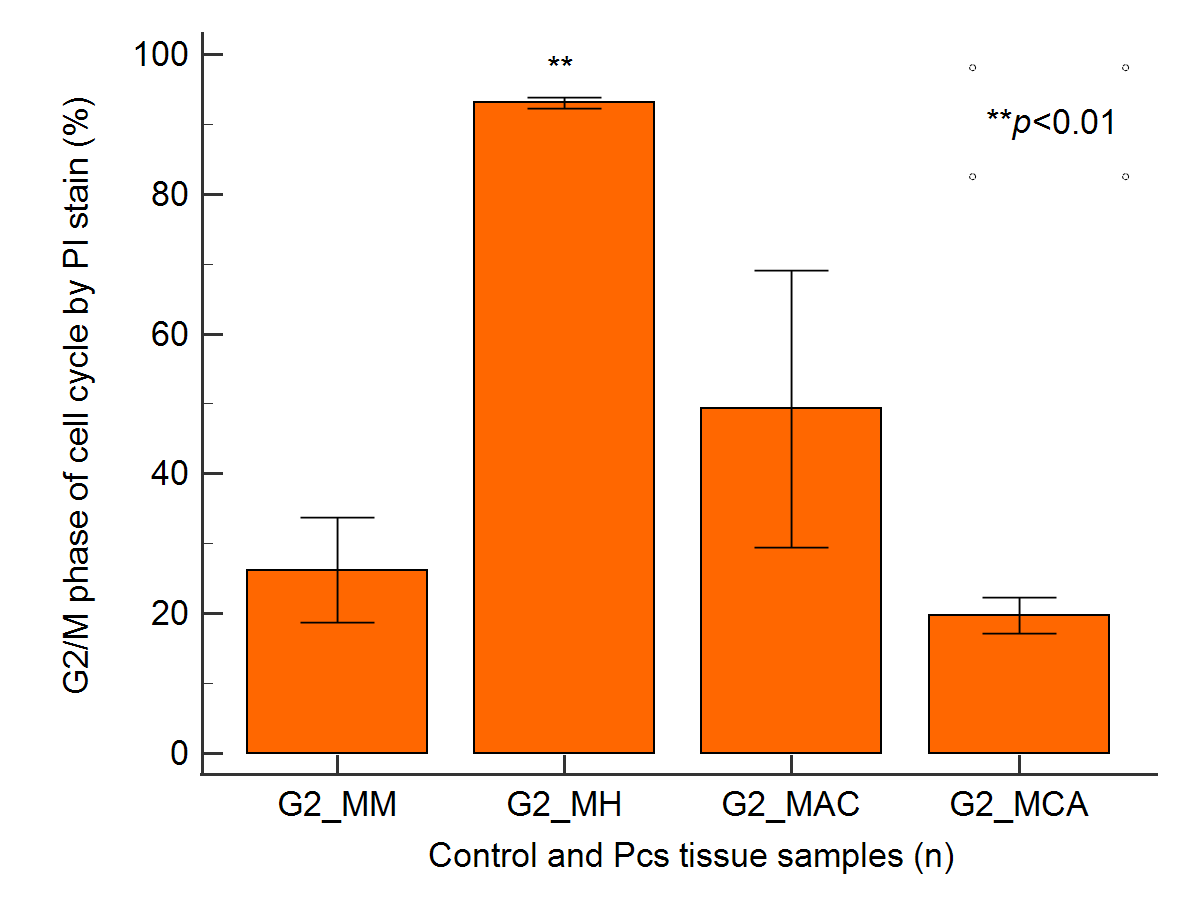

Supplement: Supplementary file 1 [file OncolRes-34-72421-s001.zip › Figure_S2/Figure_S2B.tif]

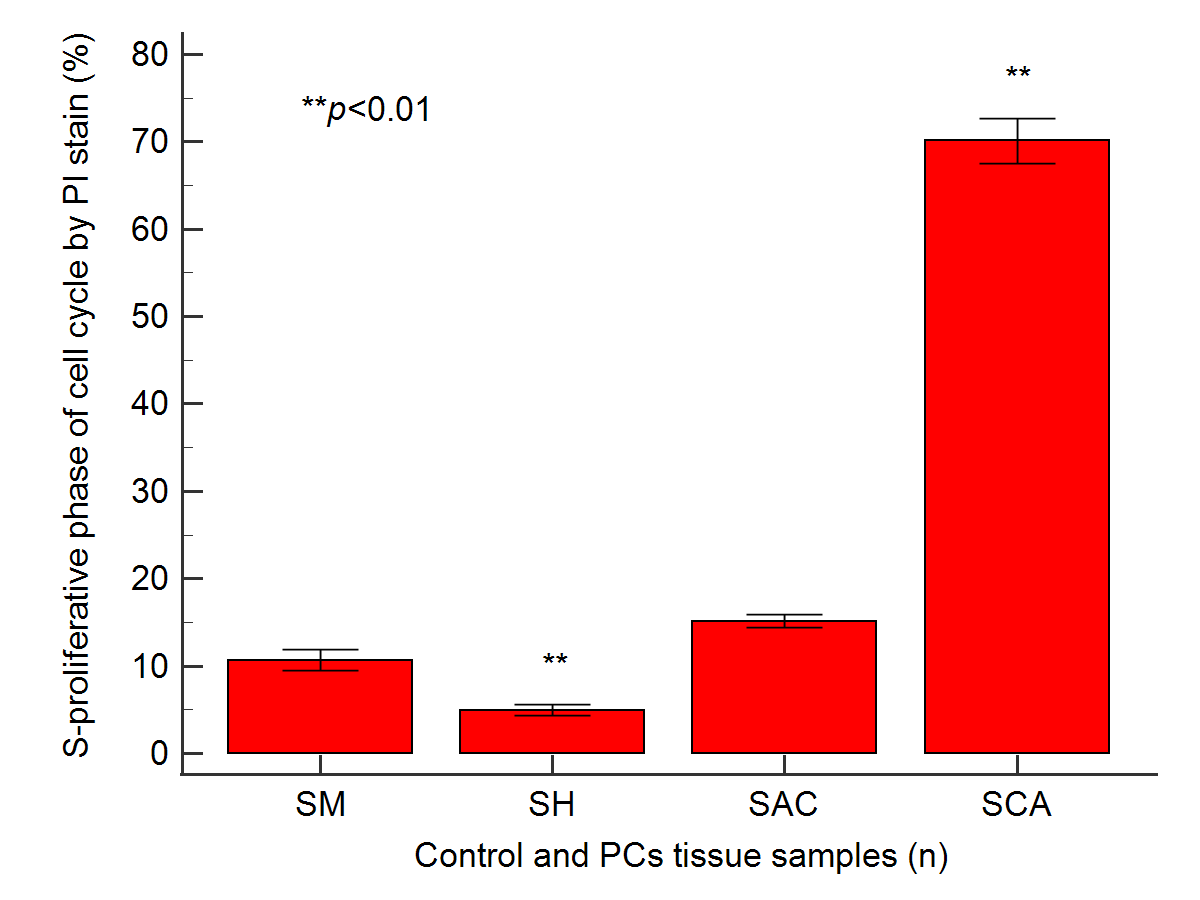

Supplement: Supplementary file 1 [file OncolRes-34-72421-s001.zip › Figure_S2/Figure_S2C.tif]

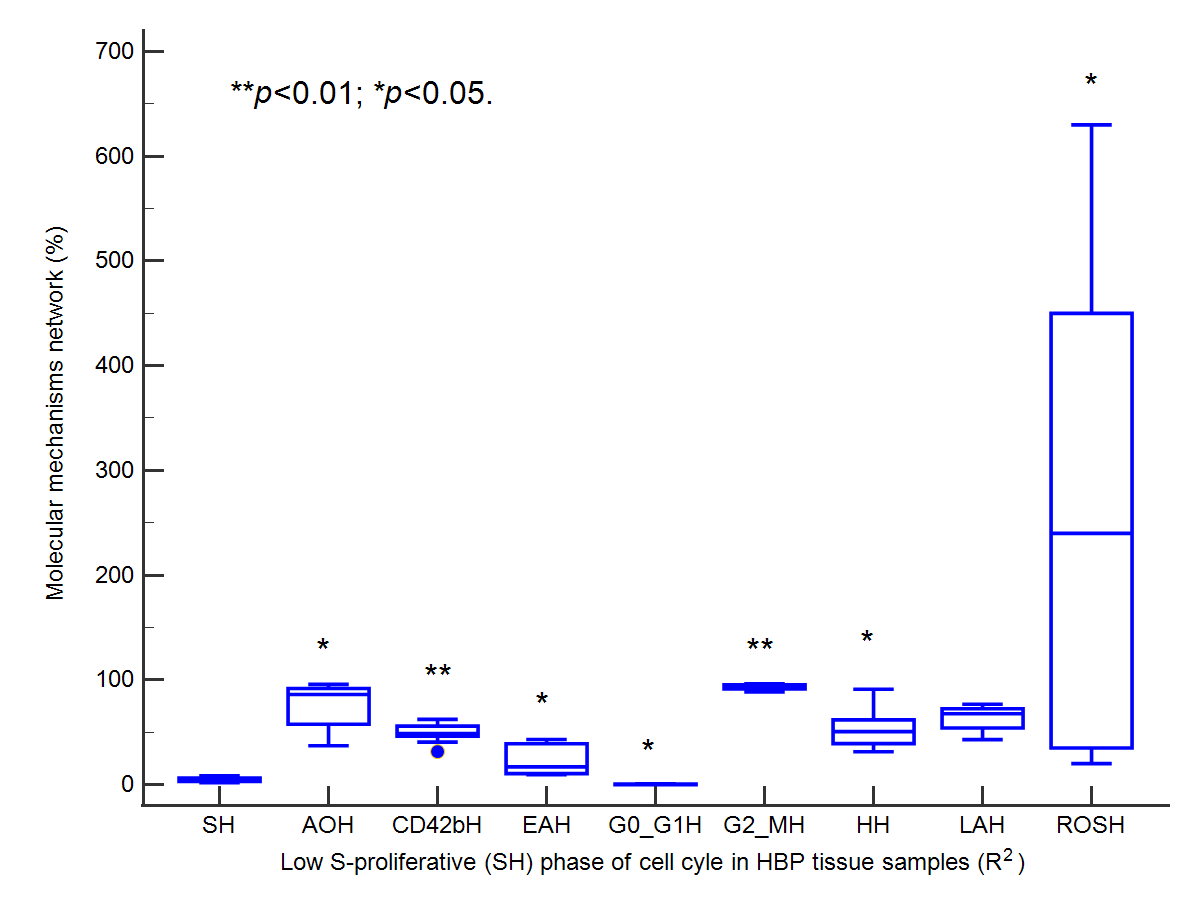

Supplement: Supplementary file 1 [file OncolRes-34-72421-s001.zip › Figure_S2/Figure_S2D.tif]

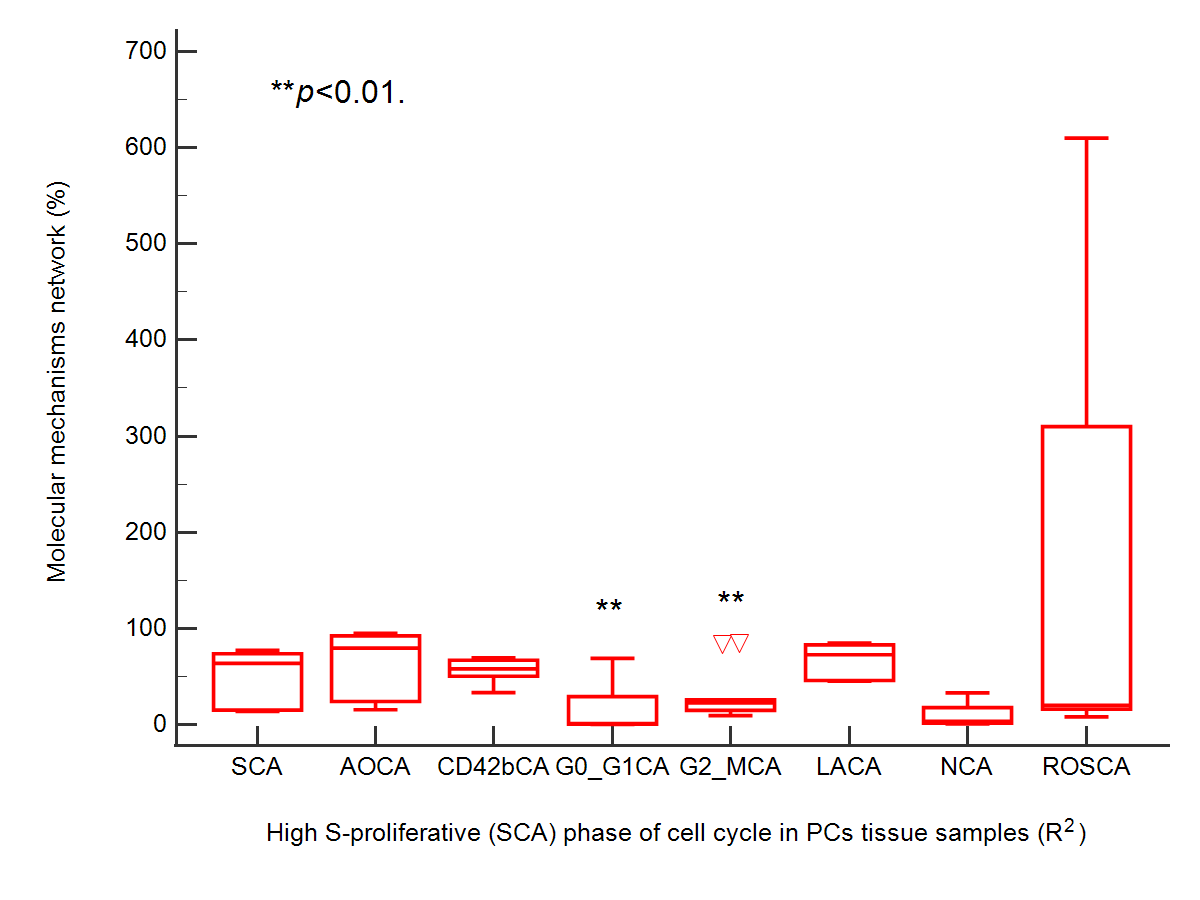

Supplement: Supplementary file 1 [file OncolRes-34-72421-s001.zip › Figure_S2/Figure_S2E.tif]

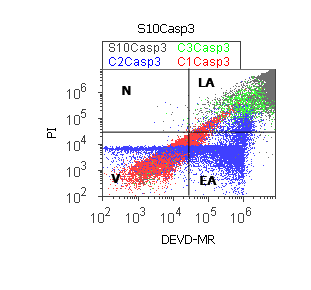

Supplement: Supplementary file 1 [file OncolRes-34-72421-s001.zip › Figure_S3/Figure_S3_S10B1.tiff]

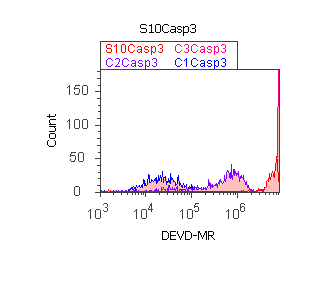

Supplement: Supplementary file 1 [file OncolRes-34-72421-s001.zip › Figure_S3/Figure_S3_S10B2.tiff]

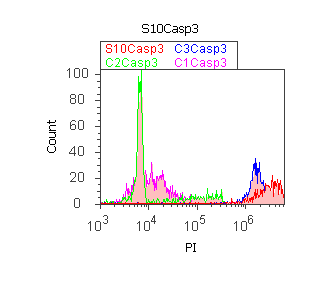

Supplement: Supplementary file 1 [file OncolRes-34-72421-s001.zip › Figure_S3/Figure_S3_S10B3.tiff]

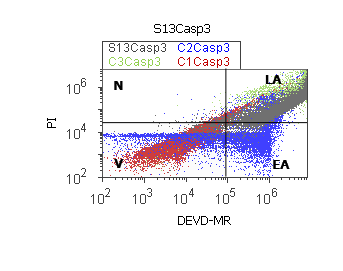

Supplement: Supplementary file 1 [file OncolRes-34-72421-s001.zip › Figure_S3/Figure_S3_S13C1.tiff]

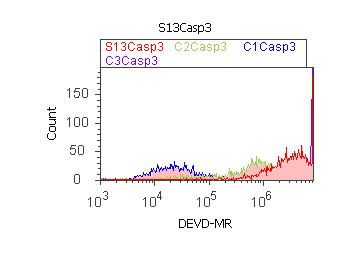

Supplement: Supplementary file 1 [file OncolRes-34-72421-s001.zip › Figure_S3/Figure_S3_S13C2.tiff]

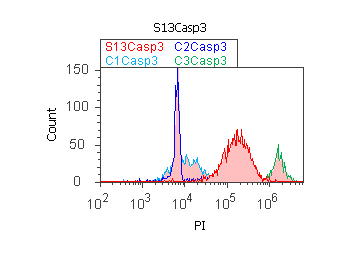

Supplement: Supplementary file 1 [file OncolRes-34-72421-s001.zip › Figure_S3/Figure_S3_S13C3.tiff]

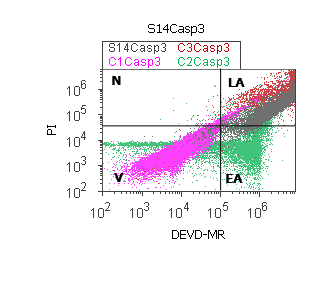

Supplement: Supplementary file 1 [file OncolRes-34-72421-s001.zip › Figure_S3/Figure_S3_S14C1.tiff]

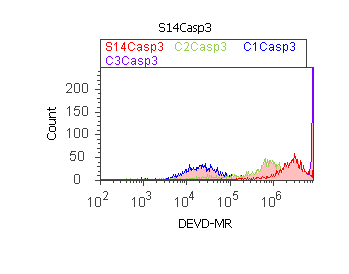

Supplement: Supplementary file 1 [file OncolRes-34-72421-s001.zip › Figure_S3/Figure_S3_S14C2.tiff]

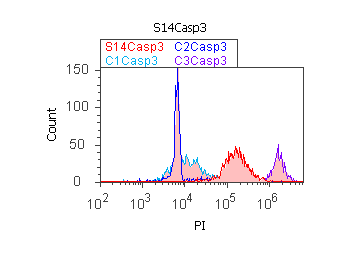

Supplement: Supplementary file 1 [file OncolRes-34-72421-s001.zip › Figure_S3/Figure_S3_S14C3.tiff]

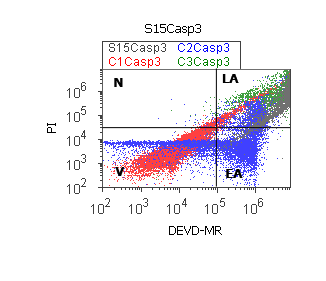

Supplement: Supplementary file 1 [file OncolRes-34-72421-s001.zip › Figure_S3/Figure_S3_S15A1.tiff]

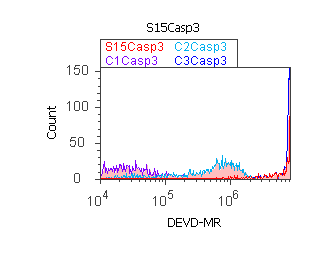

Supplement: Supplementary file 1 [file OncolRes-34-72421-s001.zip › Figure_S3/Figure_S3_S15A2.tiff]

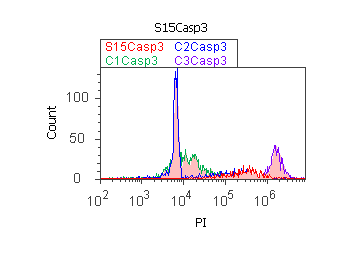

Supplement: Supplementary file 1 [file OncolRes-34-72421-s001.zip › Figure_S3/Figure_S3_S15A3.tiff]

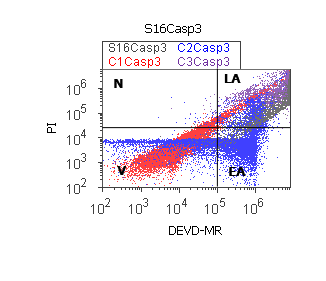

Supplement: Supplementary file 1 [file OncolRes-34-72421-s001.zip › Figure_S3/Figure_S3_S16C1.tiff]

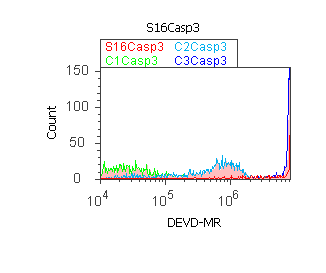

Supplement: Supplementary file 1 [file OncolRes-34-72421-s001.zip › Figure_S3/Figure_S3_S16C2.tiff]

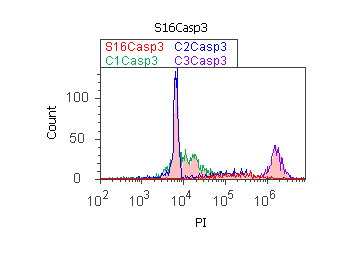

Supplement: Supplementary file 1 [file OncolRes-34-72421-s001.zip › Figure_S3/Figure_S3_S16C3.tiff]

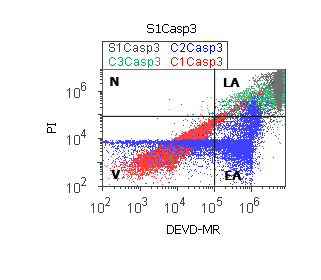

Supplement: Supplementary file 1 [file OncolRes-34-72421-s001.zip › Figure_S3/Figure_S3_S1A1.tiff]

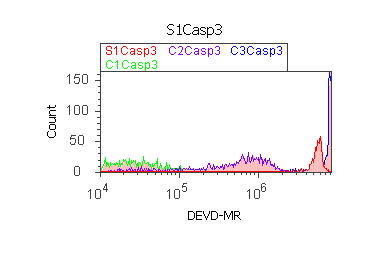

Supplement: Supplementary file 1 [file OncolRes-34-72421-s001.zip › Figure_S3/Figure_S3_S1A2.tiff]

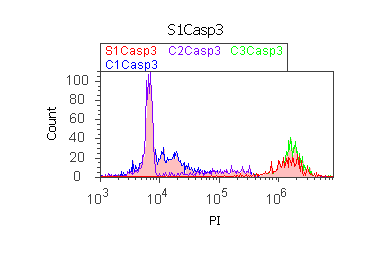

Supplement: Supplementary file 1 [file OncolRes-34-72421-s001.zip › Figure_S3/Figure_S3_S1A3.tiff]

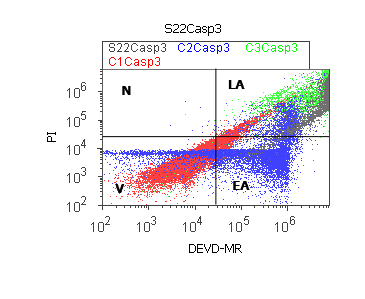

Supplement: Supplementary file 1 [file OncolRes-34-72421-s001.zip › Figure_S3/Figure_S3_S22B1.tiff]

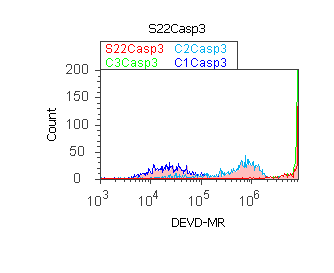

Supplement: Supplementary file 1 [file OncolRes-34-72421-s001.zip › Figure_S3/Figure_S3_S22B2.tiff]

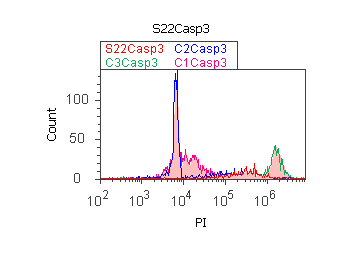

Supplement: Supplementary file 1 [file OncolRes-34-72421-s001.zip › Figure_S3/Figure_S3_S22B3.tiff]

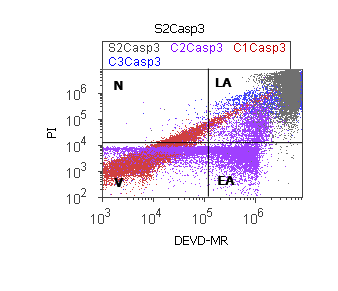

Supplement: Supplementary file 1 [file OncolRes-34-72421-s001.zip › Figure_S3/Figure_S3_S2A1.tiff]

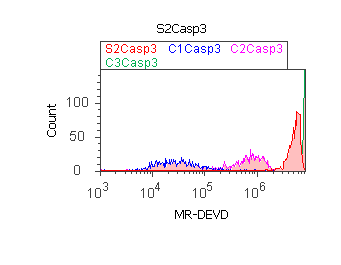

Supplement: Supplementary file 1 [file OncolRes-34-72421-s001.zip › Figure_S3/Figure_S3_S2A2.tiff]

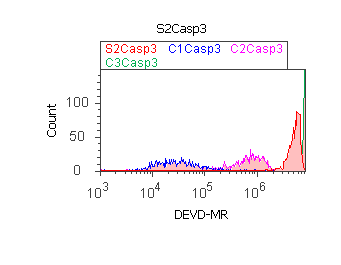

Supplement: Supplementary file 1 [file OncolRes-34-72421-s001.zip › Figure_S3/Figure_S3_S2A3.tiff]

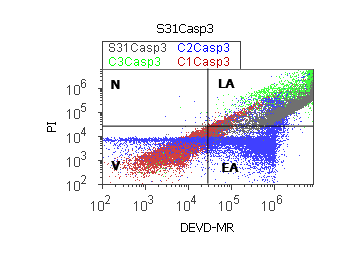

Supplement: Supplementary file 1 [file OncolRes-34-72421-s001.zip › Figure_S3/Figure_S3_S31C1.tiff]

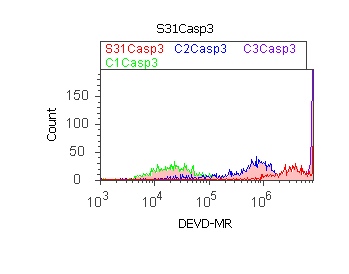

Supplement: Supplementary file 1 [file OncolRes-34-72421-s001.zip › Figure_S3/Figure_S3_S31C2.tiff]

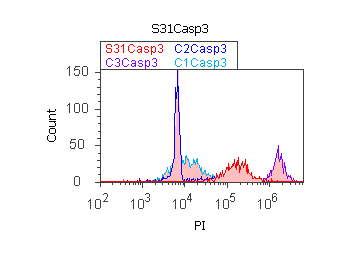

Supplement: Supplementary file 1 [file OncolRes-34-72421-s001.zip › Figure_S3/Figure_S3_S31C3.tiff]

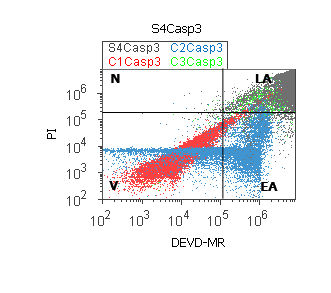

Supplement: Supplementary file 1 [file OncolRes-34-72421-s001.zip › Figure_S3/Figure_S3_S4A1.tiff]

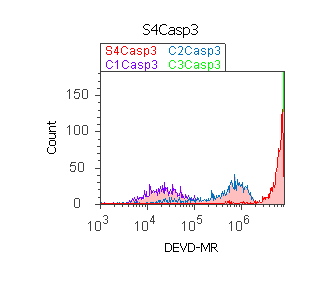

Supplement: Supplementary file 1 [file OncolRes-34-72421-s001.zip › Figure_S3/Figure_S3_S4A2.tiff]

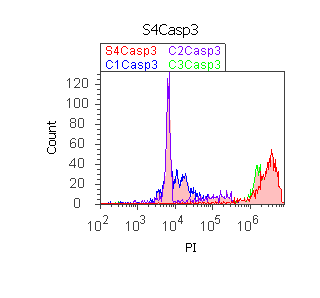

Supplement: Supplementary file 1 [file OncolRes-34-72421-s001.zip › Figure_S3/Figure_S3_S4A3.tiff]

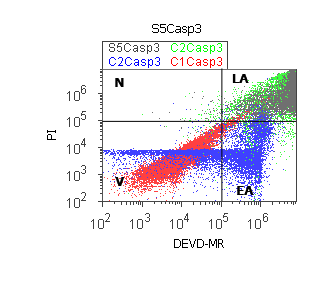

Supplement: Supplementary file 1 [file OncolRes-34-72421-s001.zip › Figure_S3/Figure_S3_S5B1.tiff]

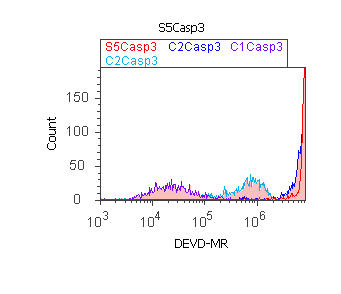

Supplement: Supplementary file 1 [file OncolRes-34-72421-s001.zip › Figure_S3/Figure_S3_S5B2.tiff]

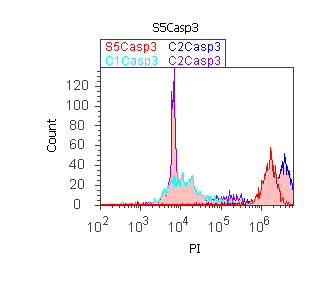

Supplement: Supplementary file 1 [file OncolRes-34-72421-s001.zip › Figure_S3/Figure_S3_S5B3.tiff]

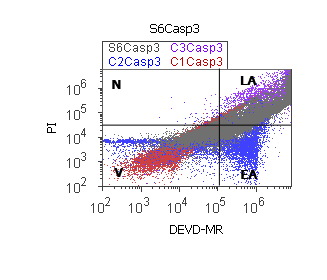

Supplement: Supplementary file 1 [file OncolRes-34-72421-s001.zip › Figure_S3/Figure_S3_S6B1.tiff]

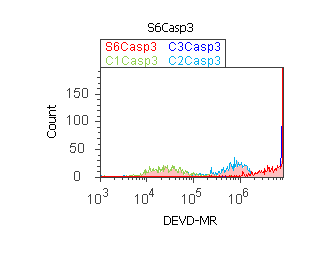

Supplement: Supplementary file 1 [file OncolRes-34-72421-s001.zip › Figure_S3/Figure_S3_S6B2.tiff]

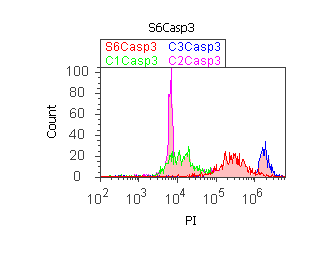

Supplement: Supplementary file 1 [file OncolRes-34-72421-s001.zip › Figure_S3/Figure_S3_S6B3.tiff]

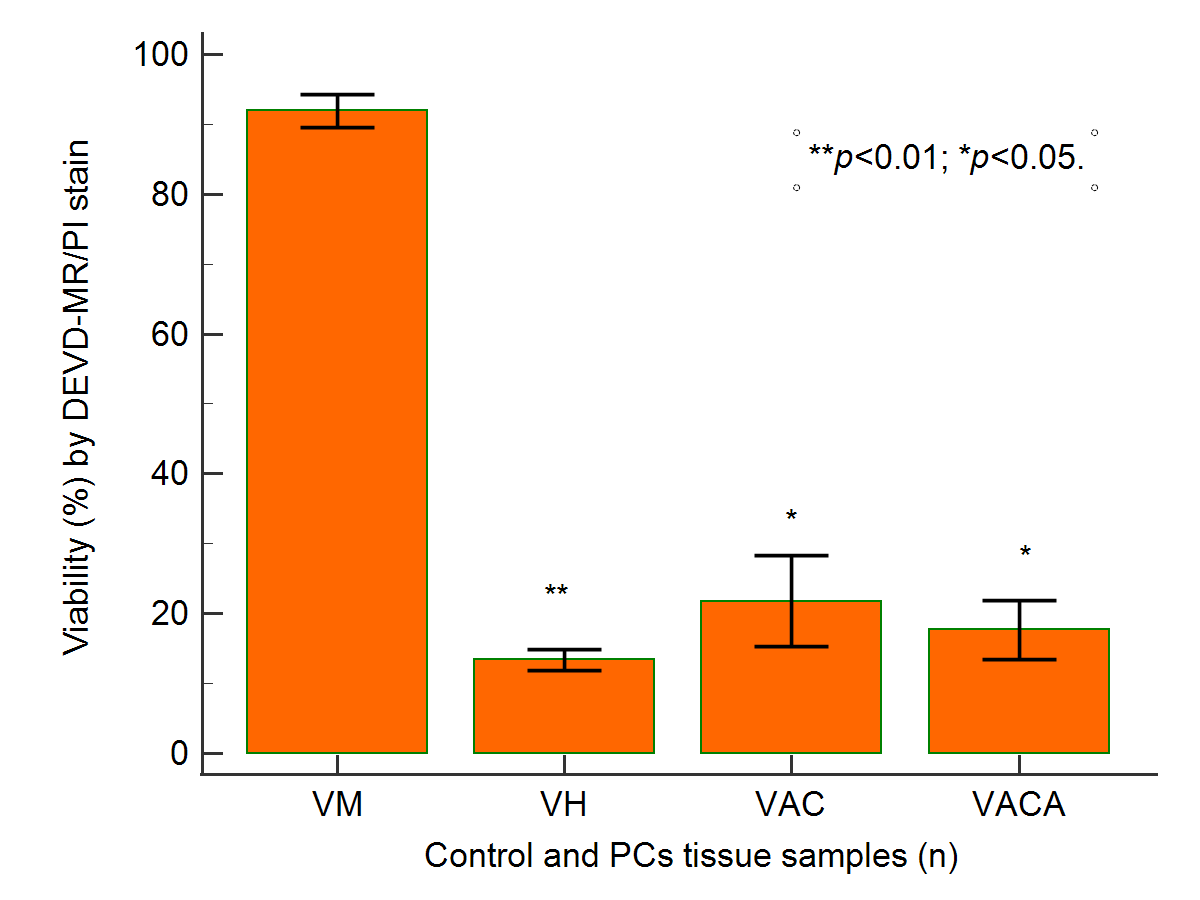

Supplement: Supplementary file 1 [file OncolRes-34-72421-s001.zip › Figure_S4/Figure_S4A.tif]

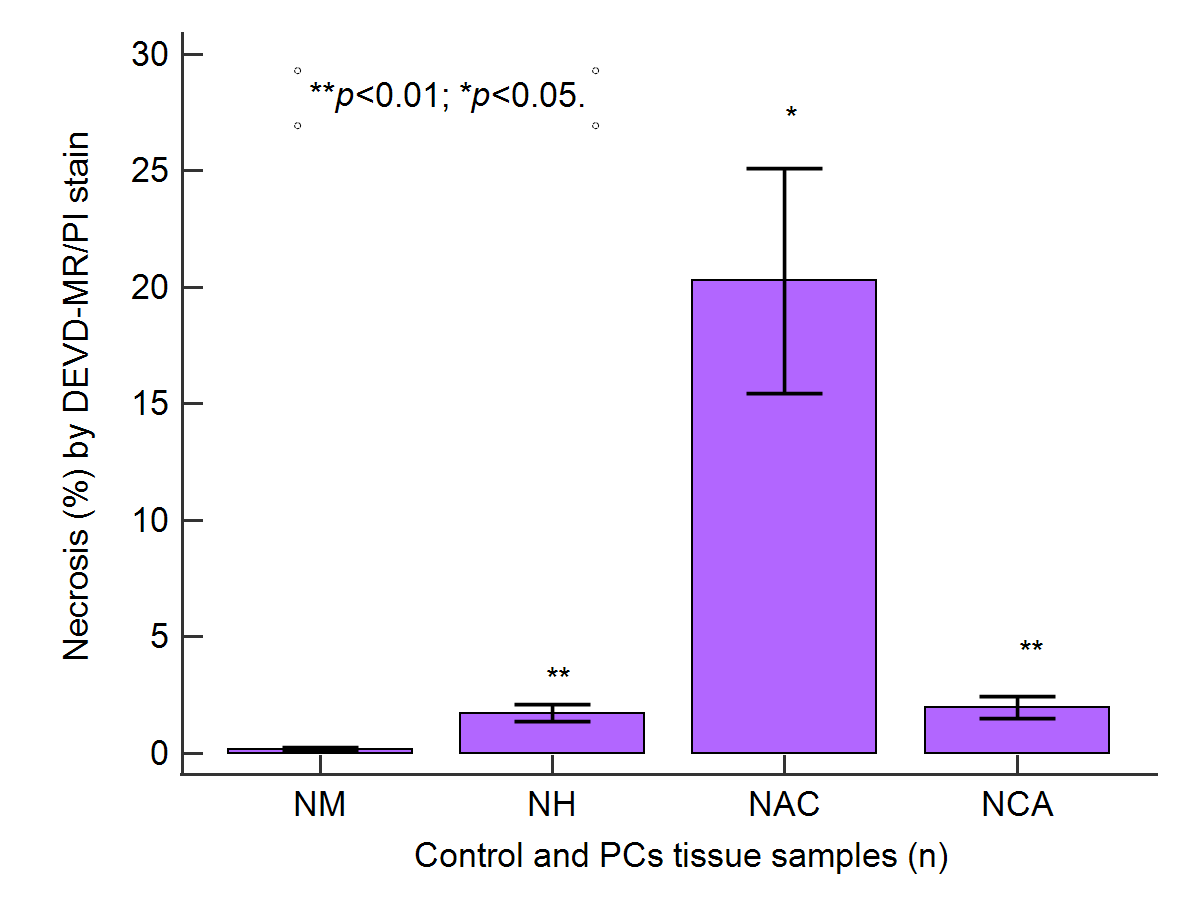

Supplement: Supplementary file 1 [file OncolRes-34-72421-s001.zip › Figure_S4/Figure_S4B.tif]

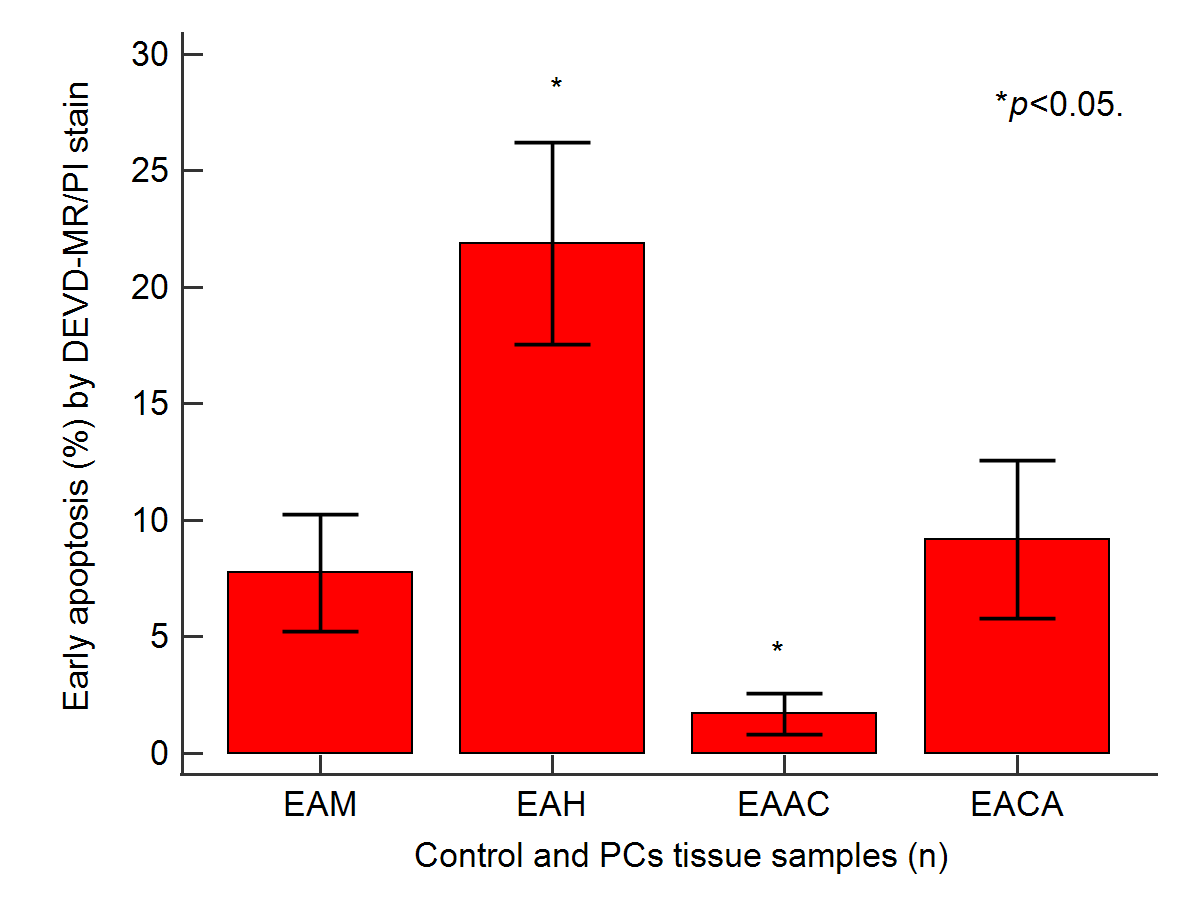

Supplement: Supplementary file 1 [file OncolRes-34-72421-s001.zip › Figure_S4/Figure_S4C.tif]

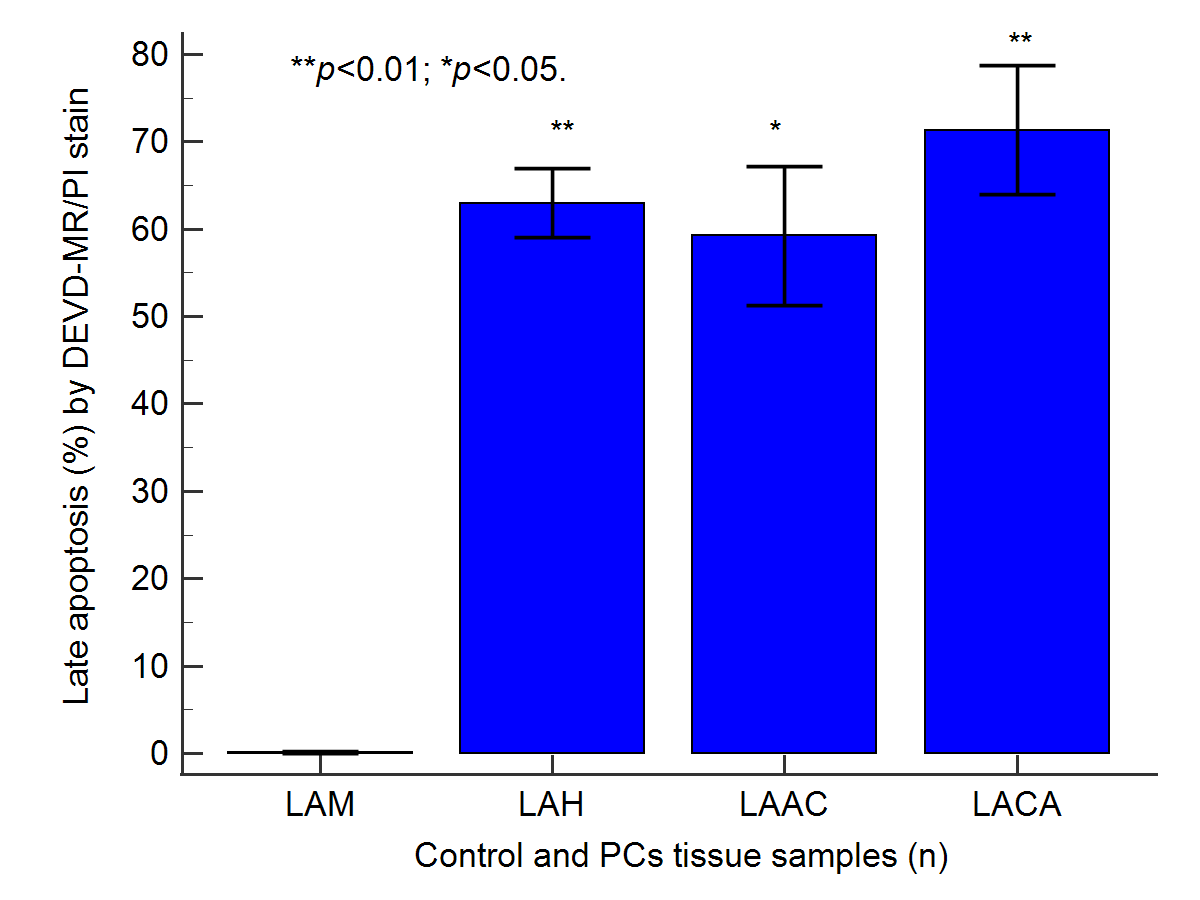

Supplement: Supplementary file 1 [file OncolRes-34-72421-s001.zip › Figure_S4/Figure_S4D.tif]

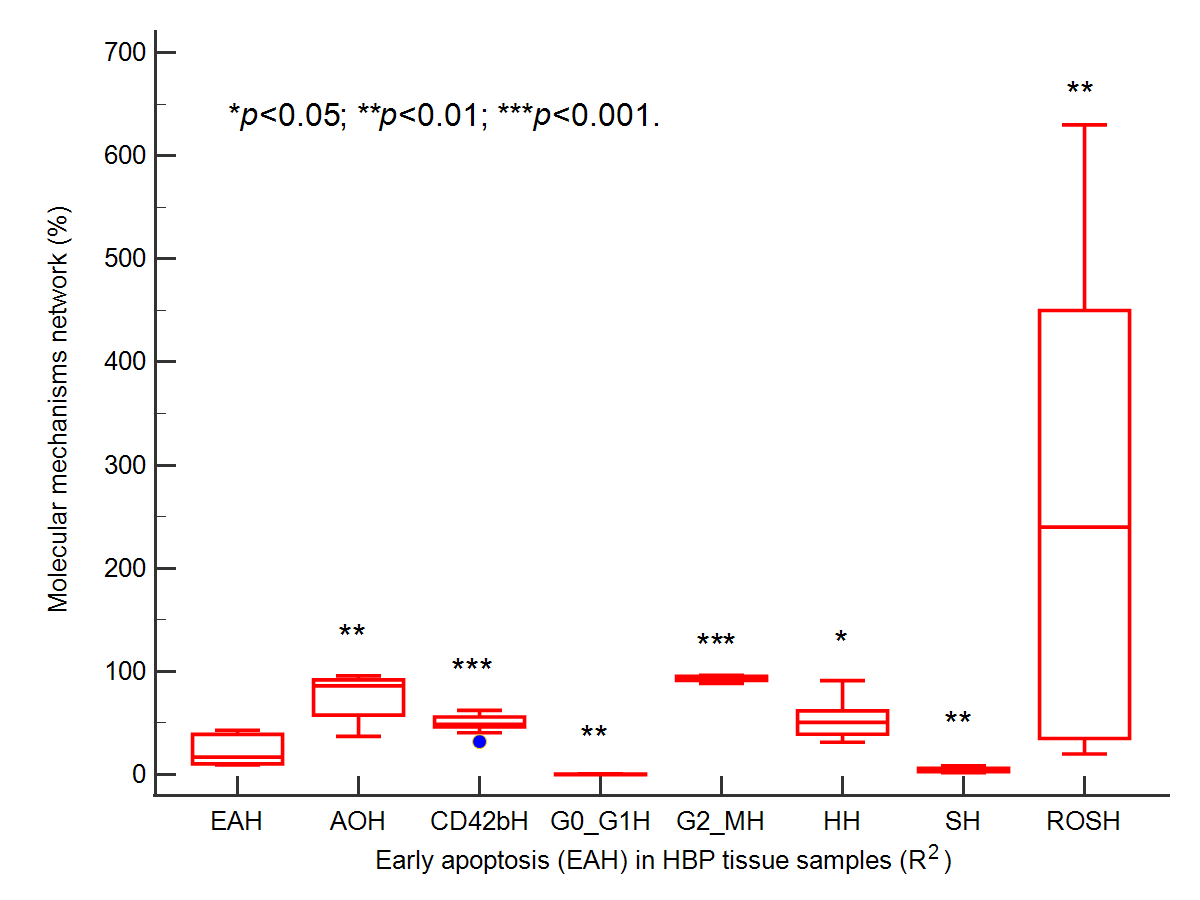

Supplement: Supplementary file 1 [file OncolRes-34-72421-s001.zip › Figure_S4/Figure_S4E.tif]

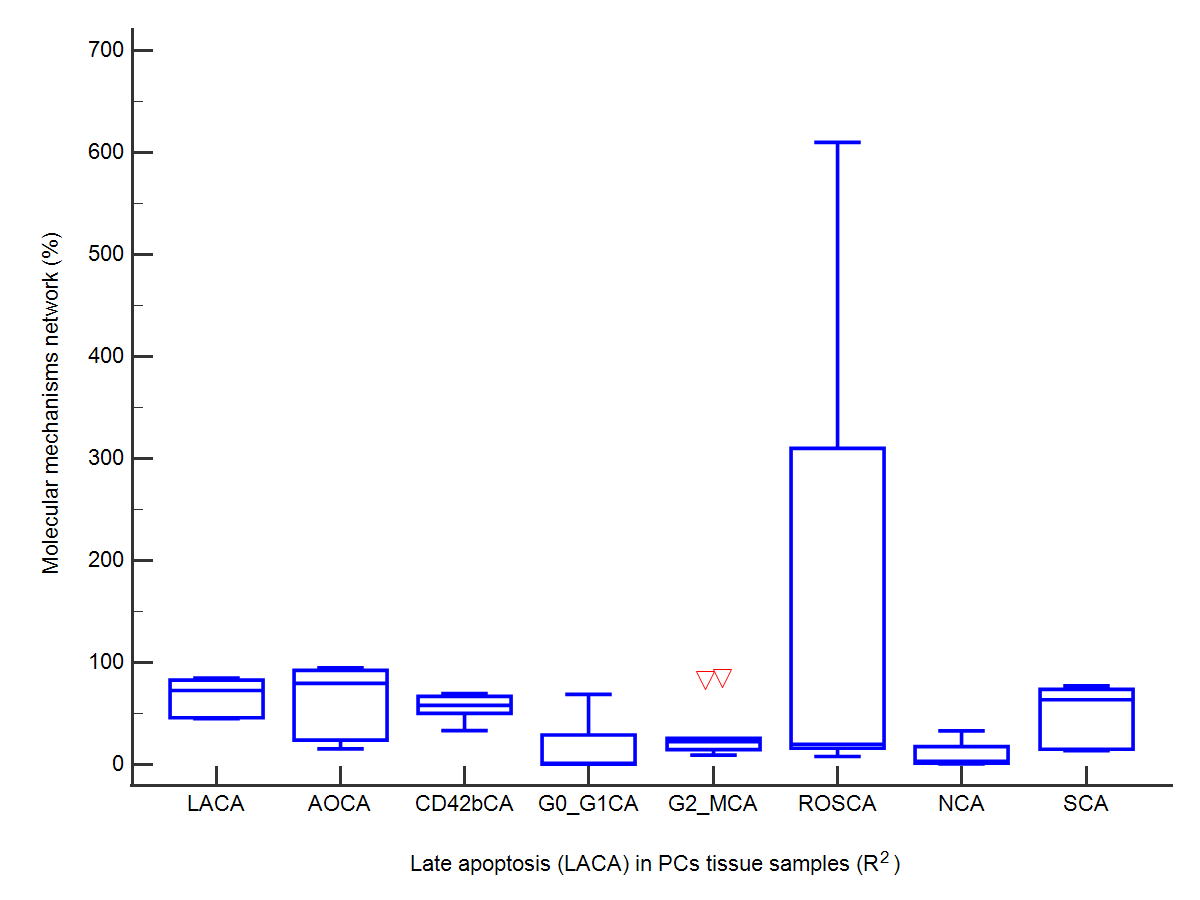

Supplement: Supplementary file 1 [file OncolRes-34-72421-s001.zip › Figure_S4/Figure_S4F.tif]

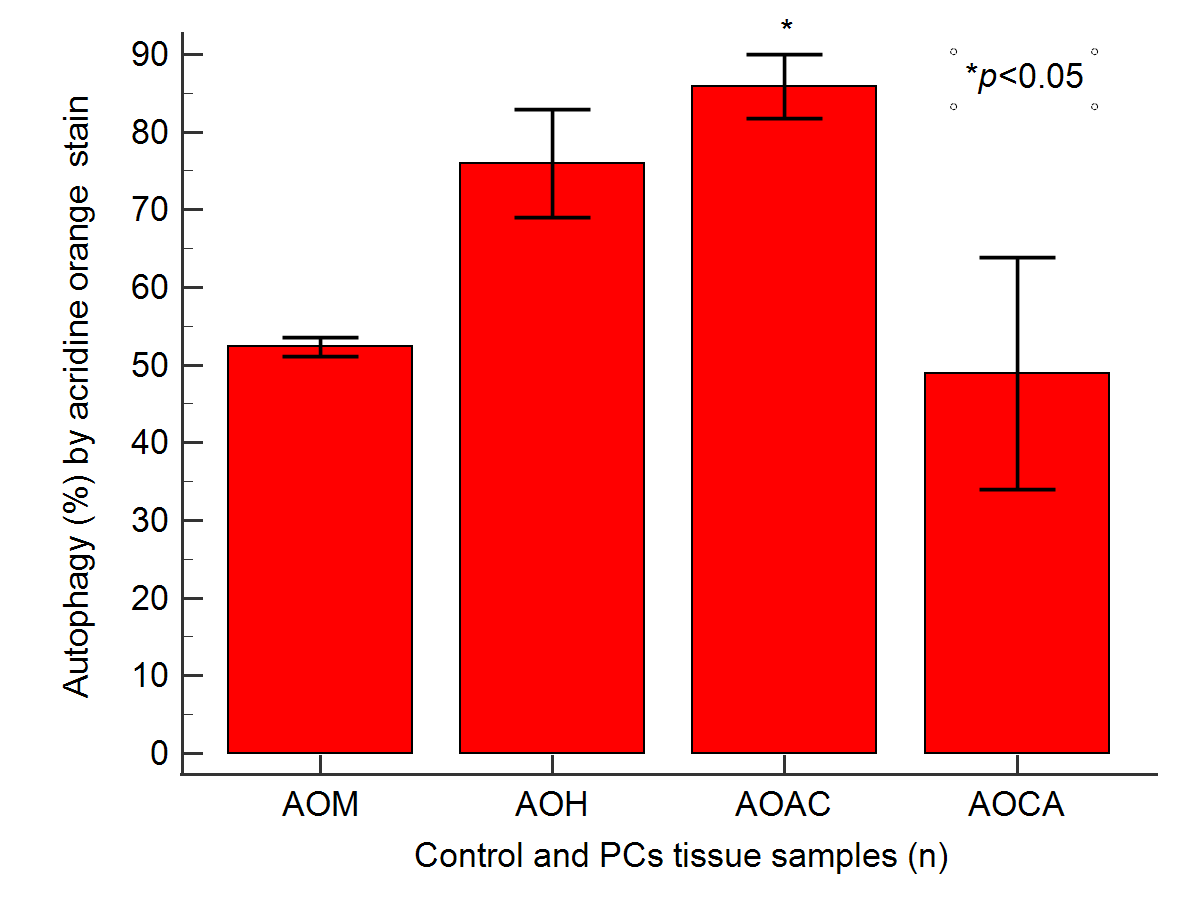

Supplement: Supplementary file 1 [file OncolRes-34-72421-s001.zip › Figure_S5/Figure_S5D.tif]

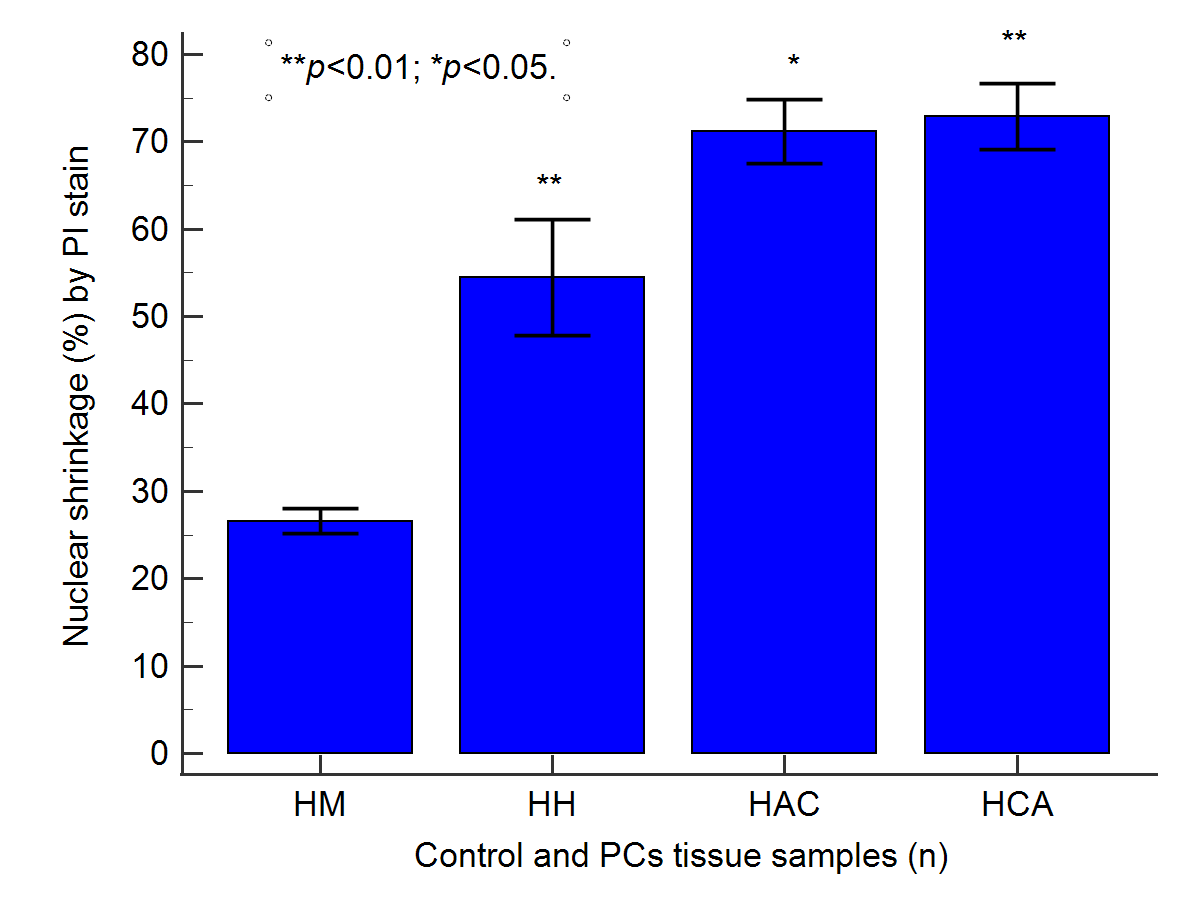

Supplement: Supplementary file 1 [file OncolRes-34-72421-s001.zip › Figure_S5/Figure_S5E.tif]

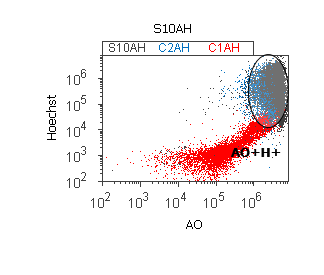

Supplement: Supplementary file 1 [file OncolRes-34-72421-s001.zip › Figure_S5/Figure_S5_S10B1.tiff]

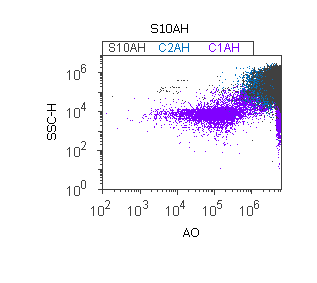

Supplement: Supplementary file 1 [file OncolRes-34-72421-s001.zip › Figure_S5/Figure_S5_S10B2.tiff]

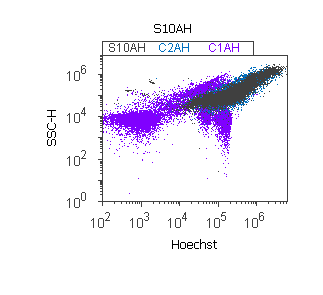

Supplement: Supplementary file 1 [file OncolRes-34-72421-s001.zip › Figure_S5/Figure_S5_S10B3.tiff]

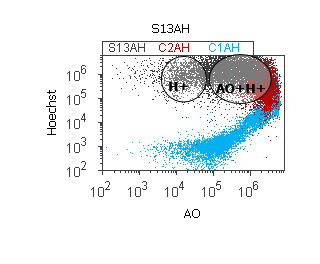

Supplement: Supplementary file 1 [file OncolRes-34-72421-s001.zip › Figure_S5/Figure_S5_S13C1.tiff]

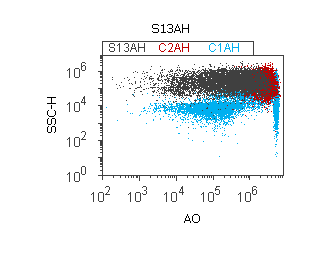

Supplement: Supplementary file 1 [file OncolRes-34-72421-s001.zip › Figure_S5/Figure_S5_S13C2.tiff]

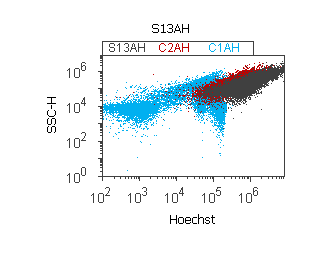

Supplement: Supplementary file 1 [file OncolRes-34-72421-s001.zip › Figure_S5/Figure_S5_S13C3.tiff]

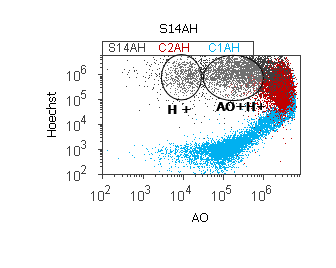

Supplement: Supplementary file 1 [file OncolRes-34-72421-s001.zip › Figure_S5/Figure_S5_S14C1.tiff]

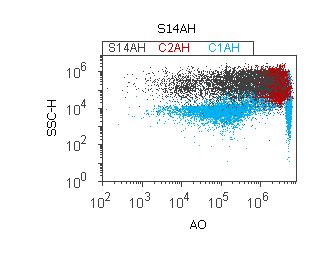

Supplement: Supplementary file 1 [file OncolRes-34-72421-s001.zip › Figure_S5/Figure_S5_S14C2.tiff]

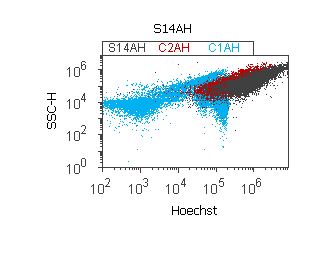

Supplement: Supplementary file 1 [file OncolRes-34-72421-s001.zip › Figure_S5/Figure_S5_S14C3.tiff]

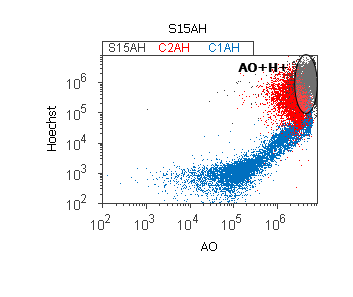

Supplement: Supplementary file 1 [file OncolRes-34-72421-s001.zip › Figure_S5/Figure_S5_S15A1.tiff]

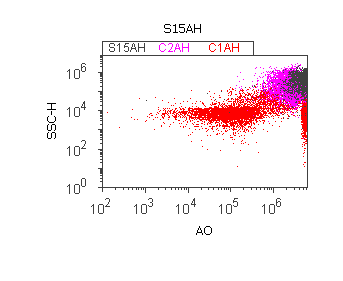

Supplement: Supplementary file 1 [file OncolRes-34-72421-s001.zip › Figure_S5/Figure_S5_S15A2.tiff]

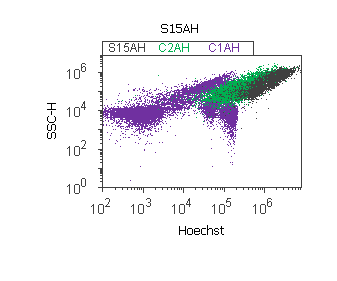

Supplement: Supplementary file 1 [file OncolRes-34-72421-s001.zip › Figure_S5/Figure_S5_S15A3.tiff]

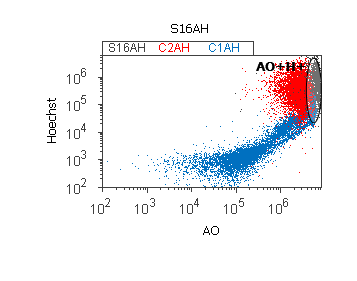

Supplement: Supplementary file 1 [file OncolRes-34-72421-s001.zip › Figure_S5/Figure_S5_S16C1.tiff]

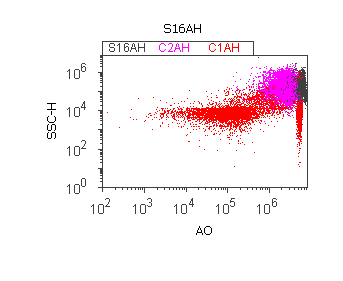

Supplement: Supplementary file 1 [file OncolRes-34-72421-s001.zip › Figure_S5/Figure_S5_S16C2.tiff]

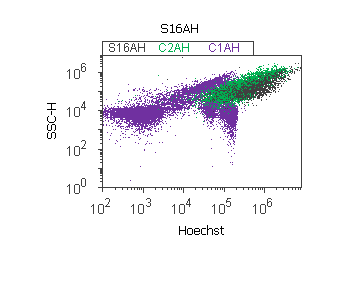

Supplement: Supplementary file 1 [file OncolRes-34-72421-s001.zip › Figure_S5/Figure_S5_S16C3.tiff]
